# Earth Abundant Oxidation Catalysts for Removal of Contaminants of Emerging Concern from Wastewater: Homogeneous Catalytic Screening of Monomeric Complexes

**DOI:** 10.3390/molecules28186466

**Published:** 2023-09-06

**Authors:** Leslie Garcia, Makynna R. Koper, Somrita Mondal, Joshua T. Priddle, William A. Truong, Elisabeth M. A. Allbritton, Ashtyn G. McAdoo, Desiray J. Cannon-Smith, Neil L. Funwie, Tuyet Hoang, Inseo Kim, David J. Hubin, Jeanette A. Krause, Allen G. Oliver, Timothy J. Prior, Timothy J. Hubin

**Affiliations:** 1 Department of Chemistry and Physics, Southwestern Oklahoma State University, Weatherford, OK 73096, USA; 2 Department of Chemistry, University of Cincinnati, Cincinnati, OH 45220, USA; 3 Department of Chemistry and Biochemistry, University of Notre Dame, Notre Dame, IN 46556, USA; 4 Department of Chemistry, School of Natural Sciences, University of Hull, Kingston Upon Hull HU6 7RX, UK

**Keywords:** dye bleaching, oxidation catalyst, cross-bridged tetraazamacrocycle

## Abstract

Twenty novel Mn, Fe, and Cu complexes of ethylene cross-bridged tetraazamacrocycles with potentially copolymerizable allyl and benzyl pendant arms were synthesized and characterized. Multiple X-ray crystal structures demonstrate the cis-folded pseudo-octahedral geometry forced by the rigidifying ethylene cross-bridge and show that two cis coordination cites are available for interaction with substrate and oxidant. The Cu complexes were used to determine kinetic stability under harsh acidic and high-temperature conditions, which revealed that the cyclam-based ligands provide superior stabilization with half-lives of many minutes or even hours in 5 M HCl at 50–90 °C. Cyclic voltammetry studies of the Fe and Mn complexes reveal reversible redox processes indicating stabilization of Fe^2+^/Fe^3+^ and Mn^2+^/Mn^3+^/Mn^4+^ oxidation states, indicating the likelihood of catalytic oxidation for these complexes. Finally, dye-bleaching experiments with methylene blue, methyl orange, and rhodamine B demonstrate efficient catalytic decolorization and allow selection of the most successful monomeric catalysts for copolymerization to produce future heterogeneous water purification materials.

## 1. Introduction

Organic dyes have extensive applications in several industries such as food, pharmaceuticals, textiles, and cosmetics and thus are often the subject of academic scientific research focused on these same areas [1,2,3,4]. However, these dyes are potentially toxic in nature. Consequently, dyes in wastewater can cause severe damage to the aquatic environment as well as living organisms including humans. In order to overcome this environmental harm, dye degradation studies have been a topic of scientific interest over the past several decades [5,6,7]. Different methods have been implemented for dye degradation, e.g., oxidation of organic dyes using transition metal ions via the “Fenton” mechanism [8] and photocatalysis using semiconductor metal oxides and sulfides [9,10]. However, these conventional methods may suffer from several diverse drawbacks, depending on each specific system. Examples of issues hampering dye degradation systems include stability over a narrow pH range, low surface area, high cost, and limited applicability in visible light [8,11].

Transition metal complexes with cross-bridged macrocyclic ligands have been broadly explored for both research purposes and commercial purposes. Cross-bridged tetraazamacrocyclic transition metal complexes have been a prevalent candidate for substrate- and mechanism-flexible oxidation catalysis over the past two decades [12,13,14,15,16,17,18,19,20,21,22]. The ethylene cross-bridged tetraazamacrocycle transition metal complexes have been well-explored in biomedical imaging [23,24,25,26,27,28,29,30,31] and for anti-cancer therapy [32,33], owing to their extreme kinetic stability. However, studies pertaining to their dye-bleaching application have been limited.

**Scheme 1:**
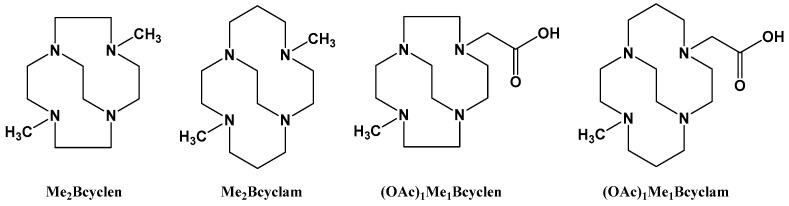
Previously studied ethylene cross-bridged tetraazamacrocyclic ligands.

The Busch catalyst, a Mn complex of the di-methylated ethylene cross-bridged cyclam **Me_2_Bcyclam** (Scheme 1) [14], is a patented catalyst used for activation of peroxide or oxygen in water. In our previous study, we reported the functionalization of the original Busch catalyst with methyl and -OAc groups at the non-cross-bridged nitrogen atoms (Scheme 1) and their application in the degradation of three model dyes, namely, methyl orange, Rhodamine B, and methylene blue [34].

**Scheme 2:**
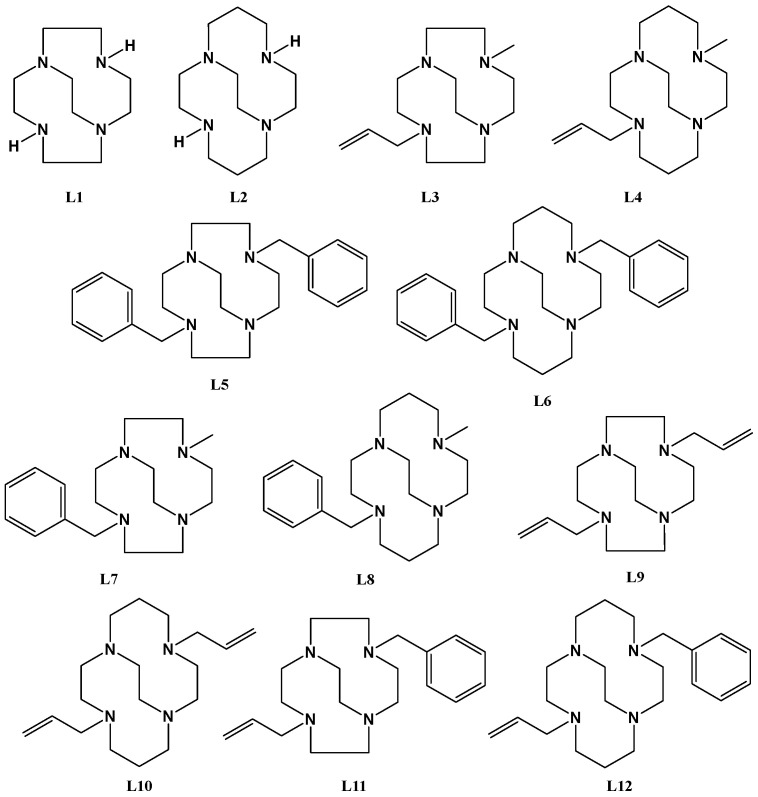
**L1**–**L12** structures used in this work.

Building on our previous studies, our current study reports on the functionalization of the tetraazamacrocyclic ligand with different combinations of allyl, benzyl, and methyl groups at the non-cross-bridged nitrogen atoms (Scheme 2) and their respective Fe, Mn, and Cu complexes. The synthetic routes are facile and cost effective with significant synthetic yield. The ligands and their corresponding complexes were characterized using ^1^H and ^13^C NMR (Figures S1–S12), LCMS, elemental analysis, X-ray crystallography, and cyclic voltammetry. Further, we have performed acid decomplexation studies on the copper complexes in order to determine their kinetic stability (Figure S13).

In addition, we have expanded our efforts in exploring the ability of the metal complexes (typically Fe and Mn) to degrade the model dyes identified above. Most of the catalysts displayed significant activity in the degradation of all three dyes at 1 mM dye concentration. Thus, these metal complex catalysts can be practically applied for cheap and convenient bleaching of organic dyes, which can overcome the disadvantage of existing traditional photocatalysts.

Heterogeneous catalysis includes some liquid- or gas-phase reaction on a solid surface. Currently, heterogeneous catalysis is widely explored both in academic research and industry owing to its advantage of recyclability and easy separation of product [35]. In such cases, catalytic activity depends on many factors, such as particle size, surface area, stoichiometry, and topology of the designed catalysts. Different methods, e.g., non-covalent immobilization of catalysts within porous zeolites, clays, or embedding into silica or carbon; using a metal-organic framework (MOF) structure; and copolymerization through formation of covalent bonds, have been used to make catalysts heterogeneous [36,37,38,39]. The method of grafting or immobilization has drawbacks such as poor selectivity and catalytic activity. Therefore, direct heterogenization through covalent bond formation is a potential method to synthesize robust heterogeneous catalysts [36]. Rigidity of the building blocks associated with strong covalent bonds is highly desirable for stability of heterogeneous polymeric frameworks [40].

Attaching allyl and benzyl functional groups furthers the goal to explore the polymerization of our catalysts through covalent bond formation. These ligands were chosen as potential co-monomers that could be used to transform the homogeneous catalysts discussed in this paper into polymeric, heterogeneous catalysts. Thus, this study may lead to the successful design of a series of potential heterogenous catalysts for degradation of organic pollutants leading to wastewater purification in a simple, convenient, and cost-effective way. The polymerization, characterization, and efficacy of these heterogeneous materials are the subject of ongoing work and will be disclosed in later publications. Below, we comment on each planned polymerization reaction type to explain why benzyl and allyl pendant arms have been utilized for this future work.

The Friedel–Crafts reaction is a historically important method of activating and substituting aromatic rings through the action of a metal catalyst [41]. When a di-functional alkyl halide, such as dichloromethane, is used, a Friedel–Crafts polymerization can be used to link monomeric aromatics, such as macrocyclic ligands that contain a benzyl group [42]. Specifically, the benzyl pendant arms may be polymerized by Friedel–Crafts polymerization following the work of Yoon, where salphen coordination complexes were polymerized through their aromatic rings [36]. Application of this method of polymerization to our benzyl ligands is ongoing in our lab. 

Alkene functional groups are the prototypical polymerizable entity, with the ability to polymerize as catalyzed by heat, acid, base, and other prompts [43]. Allyl pendant arms have a terminal alkene functional group for this purpose within our experimental design. The allyl-pendant-armed ligands and complexes can copolymerize following the work of Zuo for ethylene glycol di-methacrylate (EGDMA) polymerization [44]. This copolymerization has been quite successful in our hands and will be the subject of future manuscripts.

## 2. Results and Discussion

### 2.1. Synthesis

Ligands **L1** [45], **L2** [45], **L5** [45], **L6** [45], **L7** [33], and **L8 [32]** were synthesized by literature procedures. Metal complexes Mn**L1**Cl_2_, Fe**L1**Cl_2_, Mn**L2**Cl_2_, Fe**L2**Cl_2_, Mn**L5**Cl_2_, Fe**L5**Cl_2_, Mn**L6**Cl_2_, Fe**L6**Cl_2_ [13], and Fe**L8**Cl_2_ [46] were prepared as previously published.

**Scheme 3:**
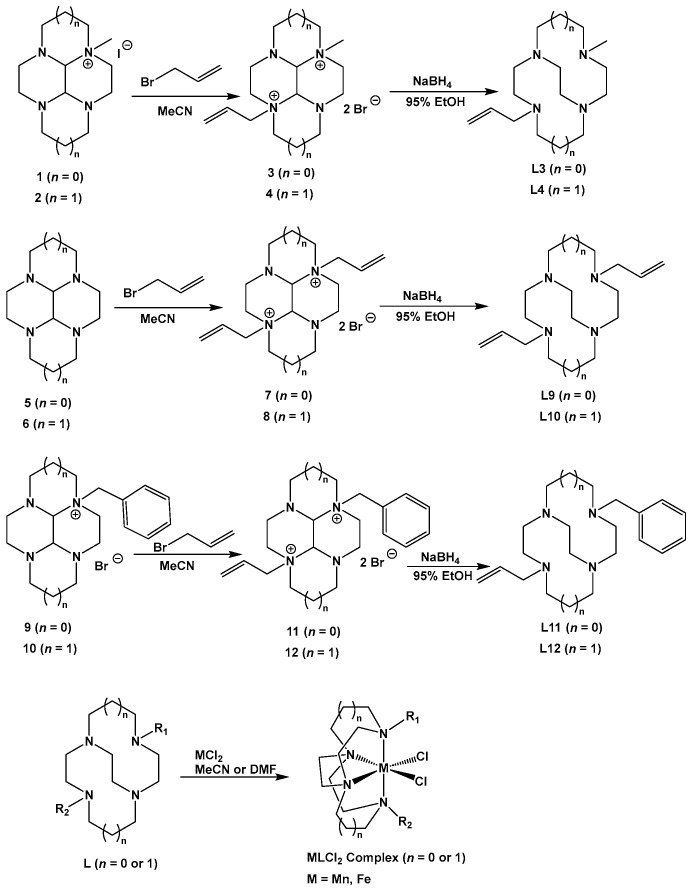
Combined synthetic scheme for the synthesis of new ligands and all metal complexes.

The homogeneous ligands and catalysts were produced according to Scheme 3, following synthetic methods that are now common for synthesizing cross-bridged tetraazamacrocycles. Generally, the tetracyclic glyoxal-tetraazamacrocycle condensate (Scheme 3, structures **5** or **6**) can be mono- or di-alkylated by haloalkyl electrophiles. Mono-alkylation is often controlled by low numbers of equivalents of the alkylating agent and non-polar solvent (THF), which causes the mono-quaternary salt to precipitate and avoid further reaction. The mono-methylated compounds **1** and **2** were prepared according to the literature [47,48], as were mono-benzylated compounds **9** and **10** [48]. Mono-alkylated compounds were then reacted with excess allyl bromide in more polar solvents (CH_3_CN), at longer reaction times, and in some cases at higher temperatures, to produce di-quaternary mono-allylated products **3**, **4**, **11**, and **12**. Reductive ring cleavage with NaBH_4_ produced the cross-bridged ligands **L3**, **L4**, **L11**, and **L12**. Di-allyl ligands **L9** and **L10** were made from **5** and **6** using excess allyl bromide to produce **7** and **8**, which were also reduced to the corresponding cross-bridged tetraazamacrocycle ligand.

Complexation of all ligands was carried out using anhydrous CuCl_2_, FeCl_2_, or MnCl_2_ in an inert atmosphere glovebox in CH_3_CN or DMF. Cross-bridged tetraazamacrocycles are proton sponges, so anhydrous conditions and aprotic solvents are generally required to successfully make their Fe and Mn complexes [49]. Yields were often in the 50% range, as the products in most cases precipitated directly from the reaction. Identity and purities were established by electrospray mass spectroscopy and elemental analysis. Copper complexes were synthesized in order to probe the kinetic stability imparted by these ligands. This was accomplished by the published method using HCl at high concentrations (1 M or 5 M) and high temperatures up to 90 °C to decomplex the ligand. Comparisons to other known ethylene cross-bridged tetraazamacrocycle copper complexes can then place a new ligand in the hierarchy of kinetic stabilization ability.

### 2.2. X-ray Crystal Structures

Crystal data for all structures are found in Table 1, while selected bond lengths and angles are compiled in Table 2.

[Mn**L1**Cl_2_] crystallizes as well-formed colorless blocks in the non-centric space group *Cc* with a single molecule in the asymmetric unit. This space group was confirmed by the Flack parameter; the crystal structure determination was particularly good, with R = 1.33% and wR(F^2^) = 3.20% for all observed data.

**Figure 1:**
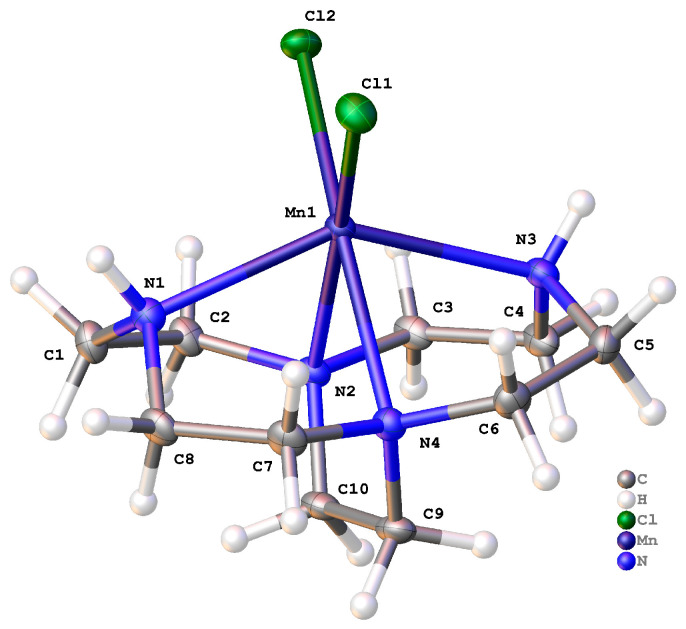
Asymmetric unit of [Mn**L1**Cl_2_] with atoms drawn as 50% probability ellipsoids.

The four nitrogen atoms of the ligand all coordinate to the central Mn^2+^ ion (Figure 1). The coordination is completed by two chloride ions to give a rather distorted octahedral geometry at the metal. There is some variation in the Mn-N bond lengths; N1-Mn1 and N3-Mn1 are 2.2761(14) and 2.2935(13) Å, respectively, while those nitrogen atoms joined by the bridge form longer bonds: N2-Mn1 = 2.3255(14) Å and N4-Mn1 = 2.3401(13) Å. The N-Mn-N bond angles for adjacent nitrogen atoms lie in the range of 72.80(5) to 77.18(5)°, but the Mn ion lies somewhat outside the pocket of the ligand such that the angle of N1-Mn1-N3 is 142.60(5)°.

In the solid state, there are hydrogen bonds (N1-H1∙∙∙Cl2 and N3-H3∙∙∙Cl1) between adjacent molecules, which form hydrogen-bonded tapes that run parallel to the crystallographic *c* axis.

[Mn**L3**Cl_2_] crystallizes in the chiral space group *P*2_1_2_1_2_1_ with a single molecule in the asymmetric unit. This space group was identified from the systematic absences and the structure refined as a racemic twin with twin fractions 0.68 and 0.32(2). There was a very good fit to the observed data, with R = 4.45% and wR(F^2^) = 7.51% for all data.

**Figure 2:**
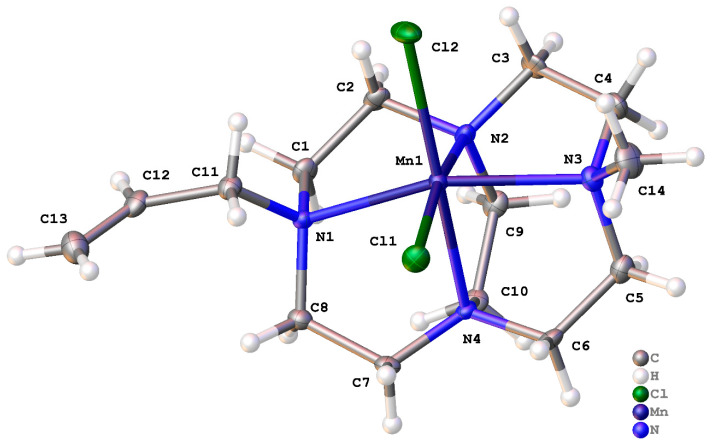
Asymmetric unit of [Mn**L3**Cl_2_] with atoms drawn as 50% probability ellipsoids.

The coordination about the Mn^2+^ ion is very similar to that in [Mn**L1**Cl_2_]; the central ion is coordinated by four nitrogen atoms from the ligand and two cis chlorides in a heavily distorted octahedral arrangement (Figure 2). However, there is a change in the relative lengths of the Mn-N bonds. In contrast to [Mn**L1**Cl_2_], here, Mn1-N1 is 2.392(3) Å and Mn1-N3 is 2.313(3) Å, but Mn1-N2 and Mn1-N4 are 2.302(3) and 2.314(3) Å, respectively. It appears that addition of substituents to the non-bridged nitrogen atoms extends the Mn-N bond lengths, presumably on steric grounds. It is notable that the methylene group (C11) and the methyl group (C14) are aligned so that H···Cl distances are approximately 3.1 Å, which may signify an interaction. The bond lengths of C11-C12 and C12-C13 in the allyl group are 1.507(5) and 1.316(5) Å, respectively, confirming the allyl group has indeed been added intact. C-H···Cl interactions are formed from the two bound chlorides to a pair of hydrogen atoms attached to one side of the ethylene bridge of an adjacent molecule related by a translation of ***a***. This interaction is repeated to generate tapes of molecules that run parallel to the crystallographic ***a*** axis. Between these tapes, there are further C-H···Cl interactions that assemble the molecules into a dense hydrogen-bonded array.

[Fe**L3**Cl_2_] adopts the same structure as [Mn**L3**Cl_2_] in space group *P*2_1_2_1_2_1_. Each of the two structures is close to being centrosymmetric but is not. The systematic absences do not suggest any other space group, but some classes of data are systematically weak but not absent. It is possible to derive a centrosymmetric model in *Pnma* in which the whole molecule is disordered over a mirror plane. The fit is much poorer and there are a large number of systematic absence violations that preclude a centrosymmetric model. The final fit in *P*2_1_2_1_2_1_ is good, with R = 4.01% and wR(F^2^) = 9.30% for all data. The coordination about the metal is very similar to the Mn analogue, with Fe1-N1 and F1-N3 distances being 2.320(3) and 2.257(3) Å, respectively. The bonds lengths from nitrogen atoms at the ethylene bridge are 2.235(4) and 2.233(4) Å. The Fe1-N1 and Fe1-N3 bonds subtend an angle of 146.15(12)° at the iron center.

We have previously reported the structure of [Fe**L5**(OAc)_2_] [50]. The coordination about the metal is very similar, except that the two chlorides are replaced by a pair of strictly mono-dentate acetate ligands. The iron to nitrogen bond lengths are 2.2091(10) and 2.2228(11) Å for the nitrogens at the ethylene bridge. The Fe-N bond length for the nitrogen atoms that have the benzyl substituents (N1 and N3) are 2.3024(10) and 2.3024(9) Å (*sic*), respectively. The Fe is slightly better contained within the pocket of the macrocycle so that the N1-Fe-N3 bond angle is 148.84(4)°.

**Figure 3:**
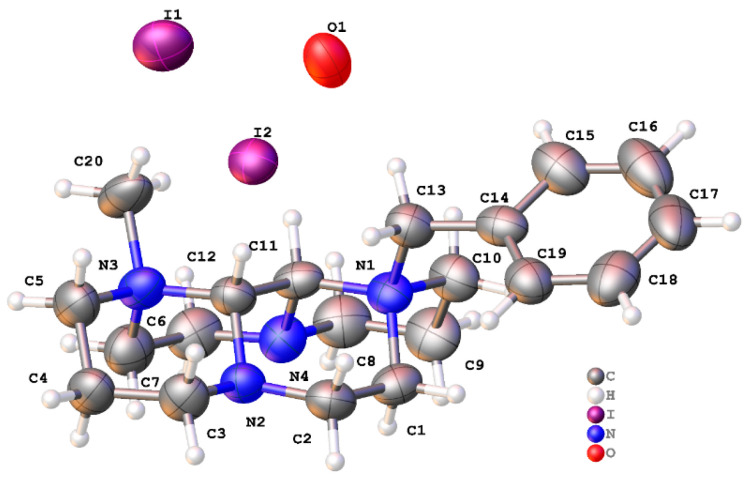
Asymmetric unit of **L8** precursor with minor disorder not shown. Atoms are represented as 50% probability ellipsoids.

A crystal structure of the known mono-methylated, mono-benzylated cyclam-glyoxal di-iodide [32] was obtained during the course of this work and is shown in Figure 3. The X-ray scattering was rather weak even though the data were collected using synchrotron radiation. However, the structure solves well in the centrosymmetric space group *P*2_1_/*n* with a single molecule in the asymmetric unit. The positive charge of the two tetraalkylammonium centers is balanced by two iodide ions. There is good evidence for disorder in the iodide positions and the single water molecule. In the solid state, the substituted cyclam ions are arranged in layers parallel to the (1 0 −1) plane and oriented so that they generate channels parallel to the crystallographic ***a*** direction. The water and iodide are found in these channels, between layers of ions.

**Figure 4:**
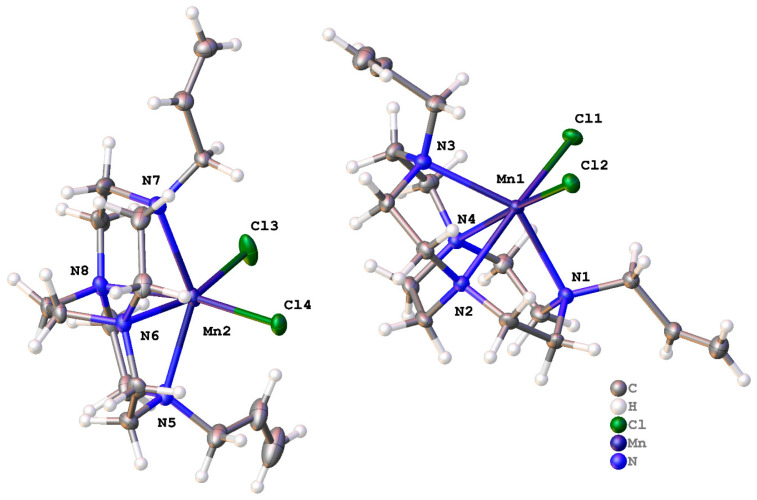
Asymmetric unit of [Mn**L9**Cl_2_] with minor disorder not shown. Atoms are represented as 50% probability ellipsoids.

[Mn**L9**Cl_2_] crystallizes in the centrosymmetric space group *P*-1 with two unique molecules in the asymmetric unit (Z′ = 2). Refinement of the model was straightforward, and minor disorder in one of the pendant allyl groups was dealt with using normal methods. The final fit was very good: R = 4.99% and wR(F^2^) = 6.45% for all data. Similar to the earlier examples, the metal ion is found in a distorted octahedral coordination geometry with a pair of cis chloride ions, as shown in Figure 4.

There are small differences in the orientation of the ligands around the metal ions in the two unique complexes, but the differences are not substantial. The familiar distorted octahedral coordination is observed, and, as in other cases, the nitrogen atoms joined by the ethylene bridge form shorter bonds to the metal than those bearing allyl substituents, as shown in Table 2, although the bonds about Mn2 are longer than those about Mn1.

The two unique complexes in the asymmetric unit form a hydrogen-bonded dimer through C-H···Cl interactions between bound chlorides of one complex and hydrogens from the ethylene bridge of the other. One of these hydrogen bonds is bifurcated such that the chloride is a hydrogen bond acceptor from two C-H groups. There is a further single trifurcated C-H···Cl hydrogen bond between pairs of these dimers, which assembles them into chains that run parallel to the (110) direction. Between these chains, additional C-H···Cl interactions generate a hydrogen-bonded three-dimensional network.

**Figure 5:**
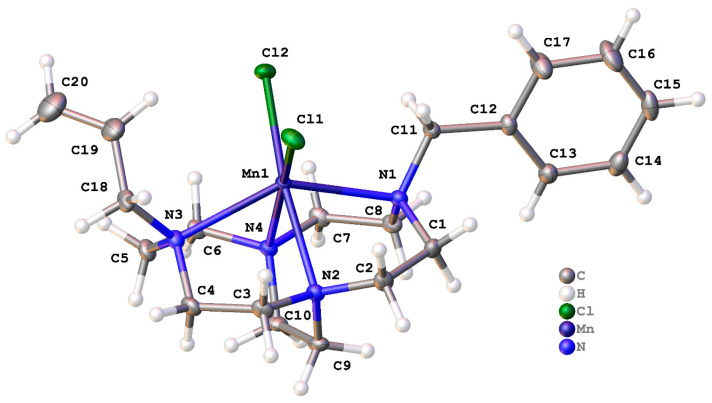
Asymmetric unit of [Mn**L11**Cl_2_] with atoms represented as 50% probability ellipsoids.

The crystal structure of [Mn**L11**Cl_2_] is centrosymmetric (space group *P*2_1_/*c*) but in other respects is similar to the other complexes described above. Once again, the fit of the model to observed data is excellent: R = 2.94% and wR(F^2^) = 6.16% for all data. The asymmetric unit contains a single Mn^2+^ ion with four nitrogen atoms from the ligand bent away from two cis chloride ions, as shown in Figure 5. Here, the nitrogen atoms of the ethylene bridge (N2 and N4) are substantially closer to the metal than N1 and N3. Mn-N2 and Mn-N4 bonds are approximately 2.30 Å, but those for N1 and N3 are elongated with values at approximately 2.40 Å (Table 2).

In the solid state, extensive hydrogen bonding between bound chloride and C-H groups produces hydrogen-bonded sheets in the *xy* plane. There are van der Waals interactions between layers, and close approach of C-H to the benzyl substituent may give rise to further weak C-H···π interactions.

**Figure 6:**
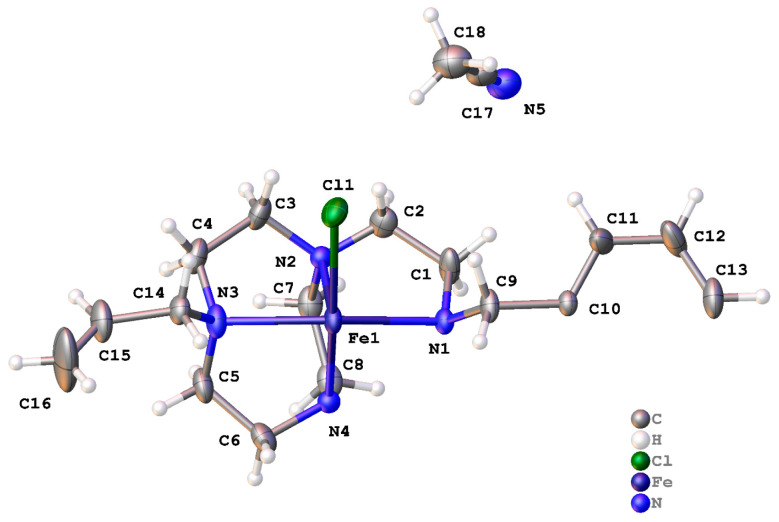
Asymmetric unit of [Fe**L11**Cl_2_] ˑ 2MeCN with atoms represented as 50% probability ellipsoids.

**Figure 7:**
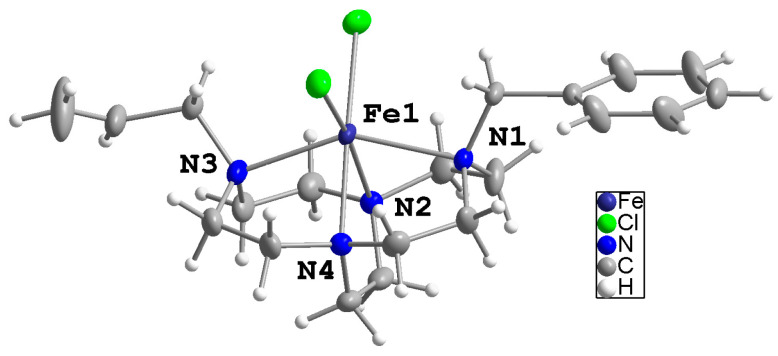
Complete [Fe**L11**Cl_2_] complex with atoms represented as 50% probability ellipsoids.

[Fe**L11**Cl_2_] crystallizes with acetonitrile within the structure to give the formula of [Fe**L11**Cl_2_] ˑ 2MeCN. The space group is *Pnma* (centrosymmetric), and the asymmetric unit contains one half complex that is disordered over a mirror plane. This added significant complication to the modeling of the observed electron density, but a satisfactory fit was possible using standard methods of disorder modeling: R(F) = 3.72% and wR(F^2^) = 6.93% (all data). The asymmetric unit is shown in Figure 6, and a representation of the whole complex appears in Figure 7. The basic coordination about the Fe^2+^ ion is similar to that in the equivalent Mn^2+^ example, but the M-N bonds are shorter, although the trend of nitrogen atoms on the ethylene bridge having shorter bond lengths is preserved, as shown in Table 2.

Focusing on the metrics about the metal centers of the **L1** and **L11** ligands (Table 2), it is apparent that the addition of substituents to the ethylene-bridged cyclen changes the coordination behavior of the ligand. [Mn**L1**Cl_2_] contains the unsubstituted ethylene-bridged cyclen, and the nitrogen atoms (N2 and N4) of the bridge are further from the metal than the two NH donors (N1 and N3). The bite angle of the two bridge nitrogen atoms is the smallest of these complexes described here. Adding substituents to N1 and N3 reverses the trend in bond lengths; in all of the other complexes, the mean bond lengths for M-N1 and M-N3 are longer than those for M-N2 and M-N4.

A trend in the bond lengths and angles is observed for the Mn^2+^ and Fe^2+^ complexes of ligands **L3** and **L11**. For **L3**, the Fe-N bond lengths are ~2.5% shorter than for Mn-N. Examination of equivalent M^2+^ structures in the CCDC [51] reproduces this trend, namely M-N bonds of the bridge are ~3.3% longer for Fe^2+^, while for the other nitrogen atoms the bond lengths are 2.0% longer. Further, the ethylene bridge bite angle (N2-M-N4) is significantly smaller for Mn, while the N1-M-N3 angle is larger for Fe. The data in the CCDC confirm this as well; the N2-M-N4 and N1-M-N3 bite angles are 3.8% and 2.9% smaller for Mn^2+^, respectively. These changes signify that the Fe^2+^ resides deeper into the cavity of the macrocycle than Mn, presumably as a consequence of the smaller size of Fe^2+^. Similar trends are observed for **L11**; Fe-N bond lengths are substantially shorter than those of Mn-N. The bite angle of N2-M-N4 is larger for Fe (77.38(12)° vs. 74.09(4)° for Mn) and the N1-Fe-N3 angle is 148.85(7)° for Fe (143.56(4)° for Mn).

### 2.3. Acid Decomplexation Studies

Cu**L**Cl_2_ or [Cu**L**Cl][PF_6_] complexes of the novel ligands studied here were synthesized whenever possible (pure **L11** and **L12** complexes could not be synthesized and were excluded from this study). Decomplexation of such copper(II) complexes of cross-bridged tetraazamacrocycles under harsh conditions of strong acid and high temperature have become a standard method for examining the kinetic stability imparted by the ligand on metal complexes with these ligands [21,22,23,31,49,52,53,54]. Due to the proton sponge nature of these highly basic tetraamines, potentiometric titrations of metal complexation reactions in aqueous solution are generally not possible, as it is impossible to fully deprotonate the ligands in water. It is therefore necessary to conduct the kinetic evaluation under harsh acidic conditions (Table 3). Cu^2+^ is generally used, as the complexes are highly colored with only one d-d absorption band, which can easily be monitored using electronic spectroscopy, allowing the pseudo-first-order kinetic analysis and half-life calculations (see Experimental Section for details). Manganese(II) and iron(II) complexes themselves lack strong d-d absorption bands, making it impossible to perform the same experiments on them. Therefore, the copper(II) complex, with the constant being the Cu^2+^ ion itself, has been adopted as the standard used in order to compare the effect of each different cross-bridged ligand on stability. This is an imperfect system, but it gives relative ligand effects. It is not meant to imply that the Mn or Fe complexes would be as stable but to give the relative effects of the differing ligand structures on kinetic stability.

Cross-bridged cyclen copper complexes are not particularly stabilized by the topological complexity and rigidity imparted by the ethylene cross-bridge [56]. The small cavity does not allow for optimal complementarity between the small ligand and the Cu^2+^ cation [57]. Even though the cross-bridge does rigidify the ligand/complex, without good complementarity, the rigidity effects do not result in large kinetic stability gains. The small cyclen macrocycle is rather rigid itself, with only ethylene chains and all five-membered chelate rings in the complex. Adding the cross-bridge simply distorts the pseudo-octahedral or square pyramidal structure, leading to minimal stabilization, if any at all, over the unbridged complexes [56]. Even so, it is interesting to probe the pendant arm effects on the stability of the copper complexes. Data were obtained, or were already available, for all cyclen complexes at 30 °C in 1 M HCl, allowing for direct comparison.

The simplest ethylene cross-bridged cyclen H_2_Bcyclen (**L1**) has a very short half-life of less than one minute, even in 1 M HCl acid conditions and at 30 °C [21]. The stability of the Cu^2+^ complexes increases in the following ligand order upon addition of substituents, making all tertiary nitrogen donors (Table 3) H_2_Bcyclen (**L1**) < Bn_1_Me_1_Bcyclen (**L7**)~Allyl_1_Me_1_Bcyclen (**L3**) < **Me_2_Bcyclen** < Allyl_2_Bcyclen (**L9**) < Bn_2_Bcyclen (**L5**). The trend(s) here are not completely transparent, but some comments can be made. It appears that a single Me group combined with one larger substituent is not optimal for stability, as **L7** and **L3** are only modestly stabilized. Two Me substituents (**Me_2_Bcyclen**) give more stability, although the effect is not large. Finally, two larger allyl or Bn substituents on the ligand (**L9** and **L5**, respectively) impart even greater stability, with **L5** exhibiting the highest stability (4.2 h vs. 51.7 min).

Ethylene cross-bridged cyclam complexes are stabilized to a much greater extent by the topological complexity and rigidity of the ethylene cross-bridge. The complementarity match, (mostly a size effect) of the larger cyclam cavity for the Cu^2+^ ion allows stabilization through the additional constraint factors [56]. As such, the Cu-Bcyclam complexes are usually studied at higher acid concentration (5 M HCl) and at elevated temperature (50 °C, 70 °C, and 90 °C). For example, Cu(**Me_2_Bcyclam**)Cl^+^ has a half-life of 79 min even at 90 °C in 5 M HCl, conditions that quickly destroy the vast majority of transition metal complexes. Data were obtained, or were already available, for all cyclam complexes at 50 °C in 5 M HCl, allowing for direct comparison.

The stability of the Cu^2+^ cyclam complexes increases in the following ligand order (Table 3): Allyl_2_Bcyclam (**L10**) < Allyl_1_Me_1_Bcyclam (**L4**)~Bn_2_Bcyclam (**L6**) < H_2_Bcyclam (**L2**) < Bn_1_Me_1_Bcyclam (**L8**) < **Me_2_Bcyclam**. Interestingly, the bulkier substituents (Bn, allyl) actually appear to destabilize the copper complexes with the cyclam ring size, rather than stabilizing them as seen for the cyclen ligands. In fact, two of the three most stable complexes, with **L2** and **Me_2_EBC**, have either only hydrogen or methyl substituents. Of the most stable cyclam complexes, **L8**, Bn_1_Me_1_Bcyclam contains a single large Bn group. Though only a hypothesis, the poor size match of the cyclen systems may gain stability as large allyl and Bn groups block access by H^+^ to the otherwise exposed (due to the severely distorted octahedral geometry) Cu^2+^-N bonds that must protonate in order for metal decomplexation to occur. On the other hand, the much better complementarity of the cyclam systems leads to less distortion of the octahedral geometries. Here, the smaller substituents (H, Me) distort the Cu^2+^-N bonds to a lesser extent, while the larger allyl and Bn substituents may disrupt the complementarity to a greater extent, leading to faster decomplexation. Regardless, what is observed is that the ethylene cross-bridged cyclam complexes, in particular, are remarkably kinetically stable under harsh acidic conditions, which would suggest oxidation catalysts (in the case of Fe and Mn) that should survive to function for significant lengths of time during the proposed water purification processes.

### 2.4. Cyclic Voltammetry

Cyclic voltammetry studies on 1.000 mM solutions of the Fe and Mn complexes in MeCN were carried out in order to correlate available reversible redox processes with their reactivity (Figures S14–S21). The results of these studies are presented in Table 4. Previous work on the Fe and Mn complexes of cross-bridged tetraazamacrocycles is included and has generally shown that higher oxidation catalyst reactivity is related to higher oxidation potentials for reaching the higher oxidation states of these metals. Copper complexes were not included in this cyclic voltammetry study, as they are not active oxidation catalysts.

The cyclic voltammetry data are useful in evaluating the oxidizing ability of the higher-valent oxidation states of these catalysts with the reversibility generally desirable for catalytic species. These ligands support Fe^2+/3+^ ions and Mn^2+/3+/4+^ ions with good reversibility in acetonitrile (see ESI for CV curves). A few complexes, those of **L1**, **L2**, **L5**, and **L6**, were only soluble in DMF, thus their data may have slight solvent effects and lack the Mn^3+/4+^ couple due to the limits of DMF solvent becoming oxidized itself. One trend that is quite consistent is that the smaller cyclen complex in a cyclen/cyclam pair having the same pendant arms is always easier to oxidize. This is likely a simple cavity size effect, with the smaller cyclen ring selecting for the smaller M^3+^ or M^4+^ cation and the larger cyclam ring selecting for the larger, lower-valent metal ion. Only the unsubstituted **L1** and **L2** show an inversion of this ring-size selectivity, which may be the result of less rigidity of these ligands due to having two secondary nitrogen donors, while all other ligands have all four nitrogen donors as more constrained tertiary nitrogen atoms.

Substituent effects are observed, although they are relatively small. For the best comparison, only those complexes examined in the same solvent (MeCN) are compared directly. For Fe complexes, replacing one methyl group with one benzyl group results in an increase in ∆E_1/2_ of ~0.03–0.06 V. For example, Fe(**Me_2_Bcyclam**)Cl_2_ and Fe**L8**Cl_2_ have E_1/2_ values of +0.110 V and +0.176 V, respectively. The change is a bit larger for Mn complexes, where ∆E_1/2_ of ~0.07–0.12 V is seen. For example, Mn(**Me_2_Bcyclen**)Cl_2_ and Me**L7**Cl_2_ have E_1/2_ Mn^3+/4+^ values of +1.232 V and +1.355 V, respectively. Replacing successive methyl groups with allyl groups for Fe complexes results in an increase in ∆E_1/2_ of ~0.04–0.06 V per allyl group. Fe(**Me_2_Bcyclam**)Cl_2_, Fe**L4**Cl_2_, and Fe**L10**Cl_2_ have E_1/2_ values of +0.110 V, +0.165 V, and +0.194 V, respectively. The change is again larger for Mn complexes, where ∆E_1/2_ of ~0.06–0.12 V is seen. For example, Mn(**Me_2_Bcyclen**)Cl_2_, Mn**L3**Cl_2_, and Mn**L9**Cl_2_ have E_1/2_ Mn^3+/4+^ values of +1.232 V, +1.338 V, and 1.385 V, respectively. These last two series show that the substituent effects are cumulative.

Interestingly, the highest oxidation potentials are found in the mixed pendant arm ligands **L11** and **L12**. The maximum E_1/2_ value for an Fe^2+/3+^ couple in a cyclen-based complex was found for Fe**L11**Cl_2_, with +0.130 V. The maximum E_1/2_ value for an Fe^2+/3+^ couple in a cyclam-based complex was found for Fe**L12**Cl_2_, with +0.196 V. The maximum E_1/2_ value for an Mn^3+/4+^ couple in a cyclen-based complex was found for Mn**L11**Cl_2_, with +1.390 V. This value is actually higher than the oxidation potential for the well-known Mn(**EBC**)Cl_2_ catalyst (+1.343 V), showing that substituent choices can serve to replace ring-size effects in order to modify oxidation potential. The maximum E_1/2_ value for an Mn^3+/4+^ couple in a cyclam-based complex was found for Mn**L12**Cl_2_, with +1.433 V, a full 0.090 V higher than for the Mn(**Me_2_Bcyclam**)Cl_2_ catalyst (+1.343 V). Di-allyl ligands **L9** and **L10** similarly raise the respective oxidation potentials of their Fe and Mn complexes, though slightly less effectively than the Allyl_1_Bn_1_ ligands **L11** and **L12** (see Table 4). The main goal of adding these particular pendant arms was to investigate copolymerization reactions that could incorporate these catalysts into water-insoluble polymers for eventual use as heterogeneous water purification materials. However, increasing their oxidation potentials might be predicted to lead to more reactivity as oxidation catalysts, which is examined below.

Future electrochemical work will need to build off what has been learned: 1. the larger cyclam ligands support higher oxidations states in general, leading to (see below) higher oxidation catalysis reactivity and 2. pendant arm effects are minimal but maximized when both an allyl and benzyl pendant arm are present in the same complex. As we continues towards the goal of heterogeneous catalysts, we will need to discern if oxidation potentials are changed when the complexes are fixed into copolymeric materials, as has been observed previously [58,59]. Fortunately, many polymers are soluble in solvents such as DMF and THF in which cyclic voltammetry can be carried out [60]. We will endeavor to track the changes in cyclic voltammetry and how they track with oxidation efficiency for polymeric compounds in future work.

### 2.5. Dye-Bleaching Studies

**Scheme 4:**
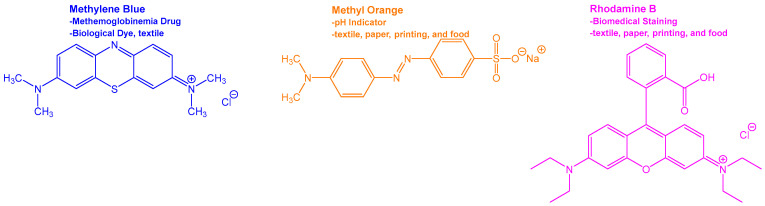
Dyes used in the dye-bleaching studies.

The goal of our overall project is to develop catalysts capable of efficient removal of organic pollutants (contaminants of emerging concern, CEC) from wastewater via oxidation catalysis. Eventually, we hope to develop catalysts such as those disclosed here into heterogeneous catalytic materials via copolymerization using the allyl and benzyl pendant arms as sites of copolymerization to incorporate the Fe and Mn cross-bridge macrocycle complex cores into water-insoluble polymers for easy separation, or perhaps as cartridge-filling material for contaminated water to be passed through with purified water exiting the cartridge. In this work, we focus on screening a large number (24) of new complexes to determine their homogeneous oxidation catalytic ability prior to selecting lead compounds for copolymerization. We have selected three extensively used dyes [1,7,9,10] (Scheme 4), methylene blue (MB), methyl orange (MO), and Rhodamine B (RhB), as model organic pollutants for our degradation study using our metal–ligand oxidation catalysts (metal = Fe and Mn with **L1**–**L12**) (Figures S22–S33). The two cationic dyes (MB and RhB) and the anionic dye (MO) have diverse functional groups including NMe_2_^+^, -SO_3_^−^, -COOH, and =NEt_2_^+^. These oxidation catalysts were able to degrade all three of the dyes in the presence of hydrogen peroxide (H_2_O_2_) as the terminal oxidant. In a survey of many potential catalysts to identify lead compounds, it is not feasible to perform the dye-bleaching experiments for all complexes at a wide range of pHs due to time and resource constraints. We chose neutral pH 7 to conduct this screening study, as we knew the complexes and dyes were stable at this pH and that most natural and municipal water sources have pHs in the pH 6–8 range [61].

Table 5 illustrates the average TOF values of control substances and oxidation catalysts for the degradation of methylene blue (MB), methyl orange (MO), and Rhodamine B (RhB). The eight complexes in red stand out among the rest as having at least one significantly higher-than-average TOF. Ranked order (1 being best) among these top eight catalysts are given in parentheses following each TOF value. The fifth column provides an overall ranked order based on an average rank order for the three dyes. For discussion purposes, the highest redox potential observed, and the kinetic stability half-life of the copper analogue (when available), are included in the table. Dye-bleaching plots, showing A_t_/A_o_ vs. time for all catalysts, can be found in the ESI, along with narrative observations for each ligand’s Fe/Mn pair of complexes. Here, we will present a broader discussion of general trends and observations about what factors lead to better dye bleaching.

**Observation 1**: Manganese catalysts generally outperform iron catalysts, which is the case for ligands **L3**–**L4**, **L6**–**L10**, and **L12**. Ligands **L5** and **L11** gave similar activity for both metals. Only iron catalysts with **L1** and **L2** gave clearly superior results. It should be noted that **L1** and **L2** have two secondary nitrogen donors, which sterically allows for dimerization to occur [13], forming LFe-O-FeL and LMn-(O)_2_-Mn structures. Dimerization is sterically prevented by all alkyl substitutions of these hydrogen atoms, even methyl groups. It is likely that the Fe-O-Fe dimers are forming in the presence of H_2_O_2_, as has been observed [13], and that these dimers are the active catalysts, as they have one open coordination site per Fe to interact with the oxidant and substrate. Perhaps the coordinatively saturated Mn dimers, lacking an open coordination site, are unable to act as catalysts. All other ligands, having alkyl groups in place of these hydrogens, are prevented from dimerizing [13], which allows the monomeric complexes to persist in solution and act as the oxidation catalysts, where Mn is more efficient than Fe, as has been proposed for the well-known Mn**Me_2_Bcyclam**Cl_2_ catalyst [12].

**Observation 2**: Cyclam-based ligands tend to produce more effective catalysts than cyclen-based ligands. Six of the eight catalysts identified as superior (red in Table 5) have cyclam ligands. Part of the reason may simply be stability, as we demonstrated that cyclam ligands were clearly superior to cyclen ligands in kinetic stability under harsh acidic conditions. For reference, the last column of Table 5 shows the stability data for the copper complexes with these ligands. Clearly, there is some loose correlation between stability and catalytic efficiency. However, these dye-bleaching experiments were performed at pH = 7, so it is likely that acid-promoted ligand dissociation is not a significant concern here. From structural information gleaned from a vast number of crystal structures of ethylene cross-bridge tetraazamacrocycle complexes reported in the Cambridge Structural Database (CSD) [51], the cyclam ligands provide a much better complementarity match (size, geometry, and electronics matching between ligand and metal) for first row transition metal ions. Therefore, the less distorted, and likely better orbital-overlapped, M-N bonds may simply lead to more reactive, or more robust, catalysts under these oxidizing conditions. Mn and Fe complexes in water with oxidizers such as H_2_O_2_ generally form insoluble oxides, a thermodynamic sink that leads to inactivation of many aqueous catalysts. Such a mechanism is avoided here due to the cross-bridged ligand stabilization of the complexes for both cyclam and cyclen complexes.

**Observation 3:** As demonstrated in the cyclic voltammetry experiments, higher oxidation potentials for the Fe^3+^ and Mn^4+^ species appear to lead to more efficient catalysis. Most obviously, the Fe complexes cannot achieve the +4 state and the associated high oxidation potential that this would require and are as such clearly inferior catalysts as a group. Conversely, all of the Mn complexes that were examined in MeCN can reversibly obtain the Mn^4+^ oxidation state and generally are more efficient catalysts. It is telling that the two top catalysts (ranked by “average rank ordering” each dye TOF among the top eight identified catalysts) are Mn^4+^-obtaining catalysts with observed oxidation potentials above 1.4 V: [Mn**L12**Cl_2_] at +1.433 V and [Mn**L8**Cl_2_] at 1.404 V. The other “top” Mn catalysts all achieve the Mn^4+^ oxidation state at or above 1.338 V. Clearly, catalysts that can achieve higher oxidation potentials are more active in the dye-bleaching tests we carried out. Interestingly, [Mn**L10**Cl_2_] ranks only “6”, even though its oxidation potential is 1.422 V. Thus, there are more factors at play here, even though oxidation potential appears to be a primary selector for high activity.

**Observation 4**: The three highest efficiency Mn catalysts all have a single benzyl pendant arm and either an allyl or methyl second pendant arm. This combination seems to provide the kinetic stability and oxidation potential for an efficient Mn catalyst. Perhaps the benzyl’s hydrophobicity may also be important in allowing good interaction with the organic substrate dyes, all of which contain multiple aromatic groups (Scheme 4). We targeted benzyl pendant arms as possible copolymerization groups using Friedel–Crafts polymerization [36] reactions and have begun to work towards producing such copolymers containing these monomeric catalysts as potential heterogeneous water purification materials.

Future work will need to build off the spectroscopic tracking of dye-bleaching work and what has been learned, namely, that Mn cyclam complexes with high oxidation potentials are the most efficient catalyst. We will need to begin to incorporate other substrates besides dyes, which are straightforward to track spectroscopically but not the only type of contaminant of emerging concern [62]. Antimicrobials and antibiotics are increasingly concerning to health officials as contaminants in waste water [63,64] and will thus be future substrates for our catalysts to be tested against. We will incorporate mass spectra tracking of aliquots over time to help determine rate as well as to gain information on potential mechanisms and products formed [65,66,67].

## 3. Experimental

### 3.1. General

All reagents and solvents were purchased from commercial sources and used as received. All solvents were of reagent grade and anhydrous. Electrospray mass spectra were recorded on a Shimadzu LCMS 2020 Electrospray Mass Spectrometer (Shimadzu, Kyoto, Japan). NMR spectra were collected on a Varian Bruker AVANCE II 300 MHz NMR Spectrometer instrument (Varian Bruker, Billerica, MA, USA). Electrochemical experiments were completed on a BAS100B Electrochemical Analyzer (BASi, West Lafayette, IN, USA) at a scan rate of 200 mV/s under nitrogen atmosphere. A button Pt electrode, a Pt-wire electrode, and a Ag-wire electrode were used as working electrode, counter electrode, and pseudo-reference electrode, respectively. Acetonitrile solutions of the complexes (1 mM) with tetrabutylammonium hexafluorophosphate (0.1 M) were used as a supporting electrolyte. SHE using ferrocene (+0.400 V versus SHE) or acetylferrocene (+0.680 V versus SHE) was used as an internal standard for the measurements.

### 3.2. Synthesis

Ligands **L1** [45], **L2** [45], **L5** [45], **L6** [45], **L7** [33], and **L8 [32]** were synthesized by literature procedures. Mn**L1**Cl_2_, Fe**L1**Cl_2_, Mn**L2**Cl_2_, Fe**L2**Cl_2_, Mn**L5**Cl_2_, Fe**L5**Cl_2_, Mn**L6**Cl_2_, Fe**L6**Cl_2_ [13], and Fe**L8**Cl_2_ [46] were prepared as previously published. Copper complexes were used only for kinetic decomplexation studies, for which the copper complexes of **L1**, **L2**, **L5**, and **L6** have already been published (see references in Table 3). [Cu**L11**Cl][PF_6_] and [Cu**L12**Cl][PF_6_] complexation never produced complexes with acceptable elemental analyses and are therefore not included in this study.

#### 3.2.1. Synthesis of Novel Ligands and Precursors

Synthesis of **3**. A total of 15.606 g (0.0464 mol, 1 eq) of the mono-methylated cyclen-glyoxal condensate **1** [47,48] was suspended in 400 mL of CH_3_CN in a 1 L RB flask. Then, 34.70 mL of allyl bromide (56.16 g, 0.464 mol, 10 eq) was added and the reaction was stoppered and stirred at room temperature for seven days. The product was filtered and washed (several times) with CH_3_CN and ether and subsequently dried under vacuum, yielding 17.879 g (94%) of **3**. Elemental Analysis (%): calc C_14_H_26_N_4_Br_2_ ˑ 0.9 H_2_O (426.41 g/mol): C 39.43, H 6.57, N 13.14; Found C 39.66, H 6.20, N 13.16. MS (ES) *m*/*z* = 125 (L-2Br^−^)^2+^.

Synthesis of **4**. A total of 9.935 g (0.0273 mol, 1 eq) of the mono-methylated cyclam-glyoxal condensate **2** [48] was suspended in 625 mL of CH_3_CN in a 1 L RB flask. Then, 39.27 mL of allyl bromide (49.62 g, 0.410 mol, 15 eq) was added and the reaction was refluxed for 14 days under a nitrogen atmosphere using a water condenser and heating mantle. After the reaction was cooled to room temperature, the white solid product was filtered off using a glass frit then washed with CH_3_CN and ether and dried under vacuum, yielding 11.058 g (92%) of **4**. Elemental Analysis (%): calc C_16_H_30_N_4_Br_2_ ˑ 1.3 H_2_O (461.67 g/mol): C 41.63, H 7.12, N 12.14; Found C 41.90, H 7.49, N 12.16. MS (ES) *m*/*z* = 139 (L-2Br^−^)^2+^.

Synthesis of **L3**. A total of 17.879 g (0.0436 mol, 1 eq) of **3** was stirred into 1500 mL of 95% EtOH in a 2 L RB flask. Then, 16.494 g (0.436 mol, 10 eq) of NaBH_4_ was slowly added to the reaction at room temperature and stirred under a nitrogen atmosphere for five days. Next, 6 M HCl was added slowly to the RB flask until the solution reached a pH of 2. Ethanol was evaporated off using a rotary evaporator. Next, the product was dissolved in 200 mL of 30% KOH solution and extracted with 5 × 175 mL fractions of benzene using a separatory funnel. The benzene layer was collected and dried with sodium sulfate overnight. The sodium sulfate was filtered off and the remaining solvent evaporated to give a pale yellow oily product, which was dried under vacuum to yield 8.808 g (80%) of **L3**. Elemental Analysis (%): calc C_14_H_28_N_4_ ˑ 0.4 H_2_O (259.61 g/mol): C 64.77, H 11.18, N 21.58; Found C 64.82, H 11.48, N 21.63. MS (ES) *m*/*z* = 253 (LH)^+^.

Synthesis of **L4**. A total of 11.653 g (0.0266 mol, 1 eq) of **4** was stirred into 860 mL of 95% EtOH in a 2 L RB flask. Then, 10.059 g (0.266 mol, 10 eq) of NaBH_4_ was slowly added to the reaction at room temperature and stirred under nitrogen atmosphere for five days. Next, 6 M HCl was added slowly to the RB flask until the solution reached a pH of 2. Ethanol was evaporated off using a rotary evaporator. Next, the product was dissolved in 200 mL of 30% KOH solution and extracted with 3 × 175 mL fractions of benzene using a separatory funnel. The benzene layer was collected and dried with sodium sulfate overnight. The sodium sulfate was filtered off and the remaining solvent evaporated to give a pale yellow oily product, which was dried under vacuum to yield 4.132 g (55%) of **L5**. Elemental Analysis (%): calc C_16_H_32_N_4_ ˑ 0.6 H_2_O (291.26 g/mol): C 65.98, H 11.49, N 19.24; Found C 65.87, H 11.35, N 19.30. MS (ES) *m*/*z* = 281 (LH)^+^.

Synthesis of **7**. A total of 10.200 g (0.0592 mol, 1 eq) of **5** [48] was added to a 500 mL RB flask and dissolved in 100 mL of MeCN. Then, 71.384 g (50.99 mL, 0.592 mol, 10 eq) of allyl bromide was added, and the reaction was stoppered and stirred for five days. The white solid product was collected by filtration, washed with MeCN and ether, and dried under vacuum to yield 20.200 g (78%) of **7**. Elemental Analysis (%): calc C_16_H_28_N_4_Br ˑ 0.5 H_2_O (445.24 g/mol): C 43.16, H 6.57, N 12.58; Found C 43.37, H 6.72, N 12.61. MS (ES) *m*/*z* = 139 (L-2Br)^2+^.

Synthesis of **8**. A total of 7.214 g (0.0360 mol, 1 eq) of **6** [48] was added to a 500 mL RB flask and dissolved in 100 mL of MeCN. Then, 39.201 g (28.00 mL, 0.360 mol, 10 eq) of allyl bromide was added, and the reaction was stoppered and stirred for seven days. The white solid product was collected by filtration, washed with MeCN and ether, and dried under vacuum to yield 13.528 g (81%) of **7**. Elemental Analysis (%): calc C_16_H_28_N_4_Br ˑ 1.0 CH_3_CN. 0.75 H_2_O (518.85 g/mol): C 46.30, H 7.09, N 13.50; Found C 46.52, H 7.18, N 13.11. MS (ES) *m*/*z* = 152 (L-2Br)^2+^.

Synthesis of **L9**. A total of 20.201 g (0.0463 mol, 1 eq) of **7** was suspended in 1500 mL of 95% ethanol in a 2 L RB flask. Then, 17.515 g of sodium borohydride was slowly added to it over 10 min, and the reaction was stirred under nitrogen for seven days. Next, 6 M HCl was added to the reaction until the solution reached a pH of 2. The ethanol was evaporated, and the white residue was then dissolved into 50 mL of DI H_2_O. Following this, 150 mL of 30% KOH solution was added, and the pH of the solution reached 14. Extraction was carried out with 4 × 100 mL fractions of benzene. The benzene layers were collected and dried over Na_2_SO_4_ overnight. After filtration of sodium sulfate, benzene was evaporated to give the pale yellow oily product, which was dried under vacuum to yield 8.555 g (66%) of **L9**. Elemental Analysis (%): calc C_16_H_30_N_4_ ˑ 0.4 H_2_O (285.65 g/mol): C 67.28, H 10.87, N 19.61; Found C 67.03, H 10.69, N 19.90. MS (ES) *m*/*z* = 279 (LH)^+^.

Synthesis of **L10**. A total of 13.50 g (0.0291 mol, 1 eq) of **8** was suspended in 1500 mL of 95% EtOH in a 2 L RB flask. Then, 10.999 g (0.291 mol, 10 eq) of sodium borohydride was added, and the reaction mixture was stirred under nitrogen atmosphere for five days. Next, 6 M HCl solution was added until the pH reached 2. The ethanol was evaporated, and the residual white solid was dissolved in 50 mL DI water followed by addition of 250 mL of 30% KOH solution. Extraction was carried out with 4 × 100 mL fractions of benzene. The benzene layers were collected and dried over Na_2_SO_4_ overnight. After filtration of sodium sulfate, benzene was evaporated to give the pale yellow oily product, which was dried under vacuum to yield 5.124 g (57%) of **L10**. Elemental Analysis (%): calc C_18_H_34_N_4_ (285.65 g/mol): C 70.54, H 11.18, N 18.28; Found C 70.50, H 11.64, N 18.38. MS (ES) *m*/*z* = 307 (LH)^+^.

Synthesis of **11**. A total of 22.065 g (0.0604 mol, 1 eq) of **9** [48] was suspended in 300 mL of acetonitrile. Then, 73.072 g (0.604 mol, 10 eq) of allyl bromide was added, and the reaction was stoppered and stirred for three days. The white solid product was collected by filtration, washed with MeCN and ether, and dried under vacuum to yield 24.301 g (83%) of **9**. Elemental Analysis (%): calc C_20_H_30_N_4_Br_2_ ˑ 2.0 H_2_O (522.33 g/mol): C 45.99, H 6.56, N 10.73; Found C 45.85, H 6.39, N 10.72. MS (ES) *m*/*z* = 407 (L-Br)^+^; m/z =163 (L-2Br)^2+^.

Synthesis of **12**. A total of 4.379 g (0.0111 mol, 1 eq) of **10** [48] was suspended in 50 mL of acetonitrile. Then, 13.429 g (0.111 mol, 10 eq) of allyl bromide was added, and the reaction was stoppered and stirred for three days. The white solid product was collected by filtration, washed with MeCN and ether, and dried under vacuum to yield 1.835 g (32%) of **12**. Elemental Analysis (%): calc C_22_H_34_N_4_Br_2_ ˑ 1.3 H_2_O (537.77 g/mol): C 45.14, H 6.86, N 10.42; Found C 49.35, H 6.94, N 10.11. MS (ES) *m*/*z* = 433 (L-Br)^+^.

Synthesis of **L11**. A total of 14.591 g (0.0300 mol, 1 eq) of **11** was suspended in 1000 mL of 95% ethanol in an RB flask. Then, 11.351 g (0.300 mol, 10 eq) of sodium borohydride was slowly added to it over 10 min, and the reaction was stirred under nitrogen for nine days. Next, 6 M HCl was added to the reaction until the solution reached a pH of 2. The ethanol was evaporated, and the white residue was then dissolved into 50 mL of DI H_2_O. Following this, 200 mL of 30% KOH solution was added, and the pH of the solution reached 14. Extraction was carried out with 5 × 175 mL fractions of benzene. The benzene layers were collected and dried over Na_2_SO_4_ overnight. After filtration of sodium sulfate, benzene was evaporated to give the pale yellow oily product, which was dried under vacuum to yield 6.432 g (65%) of **L11**. Elemental Analysis (%): calc C_20_H_32_N_4_ ˑ 0.1 C_6_H_6_. 1.6 H_2_O (365.13 g/mol): C 67.76, H 9.88, N 15.34; Found C 67.61, H 9.69, N 14.99. MS (ES) *m*/*z* = 329 (LH)^+^.

Synthesis of **L12**. A total of 9.243 g (0.0180 mol, 1 eq) of **12** was suspended in 1000 mL of 95% ethanol in a 2 L RB flask. Then, 6.798 g (0.180 mol, 10 eq) of sodium borohydride was slowly added to it over 10 min, and the reaction was stirred under nitrogen for eight days. Next, 6 M HCl was added to the reaction until the solution reached a pH of 2. The ethanol was evaporated, and the white residue was then dissolved into 50 mL of DI H_2_O. Following this, 200 mL of 30% KOH solution was added, and the pH of the solution reached 14. Extraction was carried out with 5 × 175 mL fractions of benzene. The benzene layers were collected and dried over Na_2_SO_4_ overnight. After filtration of sodium sulfate, benzene was evaporated to give the pale yellow oily product, which was dried under vacuum to yield 6.044 g (94%) of **L12**. Elemental Analysis (%): calc C_22_H_36_N_4_ ˑ 1.2 H_2_O (378.17 g/mol): C 69.87, H 10.23, N 14.82; Found C 69.79, H 9.84, N 15.11. MS (ES) *m*/*z* = 357 (LH)^+^.

#### 3.2.2. Synthesis of Novel Metal Complexes

##### General Procedure for Complexation of CuCl_2_, MnCl_2_, or FeCl_2_

In an inert atmosphere glove box, 0.001 mol of ligand and 0.001 mol of anhydrous MCl_2_ were stirred in 15 mL anhydrous aprotic solvent if M = Fe/Mn (either MeCN, DMF), or MeOH of M = Cu, for 24 h. Products, blue (Cu), white (Mn), or light brown (Fe), may precipitate and be filtered from the reaction solution (Fraction A) and be washed with MeCN and allowed to air dry in the glovebox. Alternatively, products may remain soluble in the reaction mixture (Fraction B). For “Fraction B” complexes, 200 mL of anhydrous ether were added to the filtrate, which resulted in the precipitation of the product, which was collected on a fine frit, washed with ether, allowed to dry while it was open to the glovebox atmosphere, and then scraped from the frit with a spatula into a vial for storage. For Cu, “Fraction C” complexes were necessary in some cases to achieve pure products: the reaction mixture was removed from the glovebox, filtered to remove trace solids, and evaporated to dryness. The residue was then dissolved in a minimum of methanol, and a methanol solution of 5 eq of NH_4_PF_6_ was added to precipitate the blue solid products, which were filtered off, washed with methanol and ether, and dried under vacuum.

[Cu**L3**Cl_2_]: MeCN. Fraction B. Yield: 0.213 g (55%). Elemental Analysis (%): calc [Cu(C_14_H_28_N_4_)Cl_2_] ˑ 1.5 H_2_O (386.85 g/mol): C 40.63, H 7.55, N 13.54; Found C 40.86, H 7.21, N 13.70. MS (ES) *m*/*z* = 352 (CuLCl)^+^.

Mn**L3**Cl_2_: MeCN. Fraction B. Yield: 0.172 g (45%). Elemental Analysis (%): calc [Mn(C_14_H_28_N_4_)Cl_2_] ˑ 0.8 H_2_O (392.66 g/mol): C 42.82, H 7.60, N 14.27; Found C 42.82, H 7.44, N 14.39. MS (ES) *m*/*z* = 342 (MnLCl)^+^.

Fe**L3**Cl_2_: MeCN. Fraction B. Yield: 0.157 g (41%). Elemental Analysis (%): calc [Fe(C_14_H_28_N_4_)Cl_2_] ˑ 0.3 H_2_O (384.55 g/mol): C 43.73, H 7.50, N 14.57; Found C 43.63, H 7.58, N 14.55. MS (ES) *m*/*z* = 343 (FeLCl)^+^.

[Cu**L4**Cl][PF_6_]: MeCN. Fraction C. Yield: 0.286 g (49%). Elemental Analysis (%): calc [Cu(C_16_H_32_N_4_)Cl_0.5_][PF_6_]_1.5_ (579.18 g/mol): C 33.18, H 5.57, N 9.67; Found C 33.15, H 5.62, N 9.65. MS (ES) *m*/*z* = 379 (CuLCl)^+^.

Mn**L4**Cl_2_: MeCN. Fraction B. Yield: 0.185 g (45%). Elemental Analysis (%): calc [Mn(C_16_H_32_N_4_)Cl_2_] ˑ 1.0 H_2_O (424.31 g/mol): C 45.29, H 8.08, N 13.20; Found C 45.18, H 8.00, N 13.37. MS (ES) *m*/*z* = 370 (MnLCl)^+^.

Fe**L4**Cl_2_: MeCN. Fraction B. Yield: 0.163 g (40%). Elemental Analysis (%): calc [Fe(C_16_H_32_N_4_)Cl_2_] ˑ 1.5 H_2_O (424.31 g/mol): C 44.26, H 8.12, N 12.90; Found C 43.87, H 7.66, N 13.27. MS (ES) *m*/*z* = 371 (FeLCl)^+^.

[Cu**L7**Cl][PF_6_]: MeOH. Fraction B. Yield: 0.289 g (79%). Elemental Analysis (%): calc [Cu(C_18_H_30_N_4_)Cl] [PF_6_] ˑ 2.5 H_2_O (481.95 g/mol): C 44.86, H 7.32, N 11.63; Found C 44.51, H 7.26, N 11.89. MS (ES) *m*/*z* = 400 (CuLCl)^+^.

Mn**L7**Cl_2_: DMF. Fraction A. Yield: 0.295 g (69%). Elemental Analysis (%): calc [Mn(C_18_H_30_N_4_)Cl_2_] (428.30 g/mol): C 50.48, H 7.06, N 13.08; Found C 50.38, H 7.24, N 12.92. MS (ES) *m*/*z* = 393 (MnLCl)^+^.

Fe**L7**Cl_2_: DMF. Fraction A. Yield: 0.308 g (72%). Elemental Analysis (%): calc [Mn(C_18_H_30_N_4_)Cl_2_] ˑ 1.0 DMF. 1.0 H_2_O (511.31 g/mol): C 49.33, H 7.49, N 13.70; Found C 49.41, H 7.66, N 13.53. MS (ES) *m*/*z* = 394 (FeLCl)^+^.

[Cu**L8**Cl]PF_6_: MeOH. Fraction C. Yield: 0.523g (84%). Elemental Analysis (%): calc [Cu(C_20_H_34_N_4_)Cl][PF_6_] ˑ 0.3 NH_4_PF_6_ (623.38 g/mol): C 38.54, H 5.69, N 9.66; Found C 38.22, H 5.75, N 9.72. MS (ES) *m*/*z* = 428 (CuLCl)^+^.

[Cu**L9**Cl]PF_6_: MeOH. Fraction C. Yield: 0.169 g (31%). Elemental Analysis (%): calc [Cu(C_16_H_30_N_4_)Cl][PF_6_] ˑ 1.0 H_2_O (540.41 g/mol): C 35.56, H 5.97, N 10.37; Found C 35.67, H 5.84, N 10.57. MS (ES) *m*/*z* = 376 (CuLCl)^+^.

Mn**L9**Cl_2_: MeCN. Fraction B. Yield: 0.238 g (59%). Elemental Analysis (%): calc [Mn(C_16_H_30_N_4_)Cl_2_] ˑ 0.5 H_2_O (413.29 g/mol): C 46.50, H 7.56, N 13.56; Found C 46.73, H 7.69, N 13.85. MS (ES) *m*/*z* = 369 (MnLCl)^+^.

Fe**L9**Cl_2_: MeCN. Fraction B. Yield: 0.188 g (46%). Elemental Analysis (%): calc [Fe(C_16_H_30_N_4_)Cl_2_] ˑ 0.5 H_2_O (414.19 g/mol): C 46.40, H 7.54, N 13.53; Found C 46.33, H 7.63, N 13.58. MS (ES) *m*/*z* = 370 (FeLCl)^+^.

[Cu**L10**Cl][PF_6_]: MeOH. Fraction C. Yield: 0.226 g (41%). Elemental Analysis (%): calc [Cu(C_18_H_34_N_4_)Cl][PF_6_] ˑ 1.5 H_2_O (540.41 g/mol): C 37.44, H 6.46, N 9.70; Found C 37.52, H 6.11, N 9.43. MS (ES) *m*/*z* = 404 (CuLCl)^+^.

Mn**L10**Cl_2_: MeCN. Fraction B. Yield: 0.217 g (50%). Elemental Analysis (%): calc [Mn(C_18_H_34_N_4_)Cl_2_] ˑ 1.0 H_2_O (450.35 g/mol): C 48.01, H 8.06, N 12.44; Found C 48.30, H 8.31, N 12.45. MS (ES) *m*/*z* = 397 (MnLCl)^+^.

Fe**L10**Cl_2_: MeCN. Fraction B. Yield: 0.195 g (45%). Elemental Analysis (%): calc [Fe(C_18_H_34_N_4_)Cl_2_] ˑ 1.5 H_2_O (460.26 g/mol): C 46.97, H 8.10, N 12.17; Found C 46.93, H 7.98, N 12.06. MS (ES) *m*/*z* = 398 (FeLCl)^+^.

Mn**L11**Cl_2_: MeCN. Fraction B. Yield: 0.253 g (56%). Elemental Analysis (%): calc [Mn(C_20_H_32_N_4_)Cl_2_] ˑ 0.5 H_2_O (424.31 g/mol): C 51.84, H 7.18, N 12.09; Found C 51.59, H 7.19, N 11.94. MS (ES) *m*/*z* = 419 (MnLCl)^+^.

Fe**L11**Cl_2_: MeCN. Fraction B. Yield: 0.386 g (85%). Elemental Analysis (%): calc [Fe(C_20_H_32_N_4_)Cl_2_] ˑ 3.5 H_2_O (518.30 g/mol): C 46.35, H 7.58, N 10.81; Found C 46.64, H 7.24, N 10.50. MS (ES) *m*/*z* = 420 (FeLCl)^+^.

Mn**L12**Cl_2_: MeCN. Fraction A. Yield: 0.147 g (30%). Elemental Analysis (%): calc [Mn(C_22_H_36_N_4_)Cl_2_] ˑ 0.3 H_2_O (487.80 g/mol): C 54.17, H 7.56, N 11.49; Found C 54.02, H 7.55, N 11.50. MS (ES) *m*/*z* = 446 (MnLCl)^+^.

Fe**L12**Cl_2_: MeCN. Fraction A. Yield: 0.162 g (34%). Elemental Analysis (%): calc [Fe(C_22_H_36_N_4_)Cl_2_] ˑ 0.6 H_2_O (494.11 g/mol): C 53.48, H 7.59, N 11.34; Found C 53.21, H 7.55, N 11.35. MS (ES) *m*/*z* = 447 (FeLCl)^+^.

### 3.3. Characterization

#### 3.3.1. Acid Decomplexation Studies

This experiment is a typical technique for comparing the kinetic stability of new cross-bridged ligands [21,22,23,31,49,52,53,54]. The d-d absorption band (generally near 600 nm) of the respective copper complexes (1 mM concentration) was studied on a Shimadzu UV-3600 spectrophotometer (Shimadzu, Kyoto, Japan) in the presence of acidic solutions of varying concentration. The decomposition behaviors of the different copper complexes in acidic conditions were monitored at increasing temperatures until they decomposed (in a matter of hours/days). As the absorption decreased over time, appearance of an isosbestic point on each spectrum confirmed the presence of a single decomposition product, likely CuCl_2(aq)_. The decomposition of copper complexes followed pseudo-first-order kinetics. The half-lives of the respective complexes were calculated from the plot of ln absorbance (absorbance at maxima of the spectra) vs. time.

#### 3.3.2. X-ray Crystallography Studies

The crystal structure data for all of the metal complexes were collected using a Bruker diffractometer employing Mo Kα radiation (λ = 0.71073 Å), a TRIUMPH curved-graphite monochromator, and either an APEX-II or PHOTON-II detector. Data were corrected for the effects of absorption using a multi-scan method within SADABS. The sample was held at 120 K in an Oxford Cryosystems nitrogen gas cryostream.

**L8** precursor: Data were collected through the SCrALS (Service Crystallography at the Advanced Light Source) program at Beamline 11.3.1 at the Advanced Light Source (ALS), Lawrence Berkeley National Laboratory. The instrument employed is based on a commercial Bruker diffractometer with a PHOTON 100 detector and an operating wavelength of 0.7749 Å. The data were corrected for the effects of beam decay and absorption using SADABS. The sample was held at 150 K in an Oxford Cryosystems nitrogen gas cryostream.

CCDC-2268469-2268475 contain the supplementary crystallographic data (Tables S1–S52) for this paper. These data can be obtained free of charge from The Cambridge Crystallographic Data Centre via www.ccdc.cam.ac.uk/data_request/cif.

### 3.4. Dye-Bleaching Studies

All of the dye-decolorization studies were performed in water at pH 7, following the procedure of Yin et al. [68]. The three dyes, methylene blue (MB), methylene orange (MO), and Rhodamine B (RhB), had concentrations of 3.35 mM, 3.83 mM, and 2.60 mM, respectively, whereas the metal complex stock solutions had a 1.00 mM concentration. For each experiment, 10.00 mL of dye solution was added to 10.00 mL of metal complex stock solution. pH was kept neutral with the usage of either 0.100 M HCl or 0.100 M NaOH. Further, water was added to each set to adjust the volume up to 25 mL. Accordingly, the final concentrations of the metal complex and three dyes, namely, MB, MO, and RhB, were calculated to be 0.40 mM, 1.34 mM, 1.53 mM, and 1.04 mM, respectively. For these studies, 0.25 mL of 30% H_2_O_2_ was added to each set to trigger the dye-bleaching process. To monitor the progress of the reaction, 1 mL reaction mixture aliquots were taken out at certain time intervals. After removal, each aliquot set was diluted to 100 mL with water in order to quench the bleaching reaction. Next, absorbance spectra were recorded, and the absorbance maxima values at 664 nm, 465 nm and 553 nm were monitored for reaction mixture containing MB, MO, and, RhB, respectively. Time zero was defined as the time of H_2_O_2_ addition. Further, the absorbance was monitored at 5, 10, 20, 30, 60, 120, 180, 240, 300 min, or more (until the dye reached ~10% of its actual absorbance). For each set, at least two trials were performed, and all the absorbance values reported are an average value of the trials.

## 4. Conclusions

Potentially copolymerizable allyl and benzyl pendant-armed ethylene cross-bridged tetraazamacrocycles were synthesized, and twenty of their novel Mn, Fe, and Cu complexes were produced and characterized. Two cis coordination cites are available for interaction with substrate and oxidant due to the cis-folded pseudo-octahedral geometry forced by the rigidifying ethylene cross-bridge, as demonstrated by multiple X-ray crystal structures. The cyclam-based ligands, in particular, provide superior stabilization with half-lives of the Cu complexes of many minutes or even hours in 5 M HCl at 50–90 °C, as determined by kinetic stability experiments under harsh acidic and high-temperature conditions. Reversible redox processes indicated stabilization of Fe^2+^/Fe^3+^ and Mn^2+^/Mn^3+^/Mn^4+^ oxidation states, supporting the likelihood of catalytic oxidation for these complexes. Finally, Mn cross-bridged cyclam complexes with at least one benzyl pendant arm performed best in dye-bleaching experiments with methylene blue, methyl orange, and rhodamine B. Future work will include selection of the most successful monomeric catalysts for copolymerization to produce future heterogeneous water purification materials.

## Figures and Tables

**Table 1:** Crystal data for X-ray crystal structures.

Identification code	[Mn**L1**Cl_2_]	[Mn**L3**Cl_2_]	[Fe**L3**Cl_2_]	**L8**-precursor	[Mn**L9**Cl_2_]	[Mn**L11**Cl_2_]	[Fe**L11**Cl_2_]. 2MeCN
Empirical formula	C_10_H_22_Cl_2_MnN_4_	C_14_H_28_Cl_2_MnN_4_	C_14_H_28_Cl_2_FeN_4_	C_20_H_34_I_2_N_4_O_2_	C_16_H_30_Cl_2_MnN_4_	C_20_H_32_Cl_2_MnN_4_	C_22_H_35_Cl_2_FeN_5_
Formula weight	324.15	378.24	379.15	616.31	404.28	454.33	496.30
Temperature/K	120	120	120	150	120	120	120
Crystal system	monoclinic	orthorhombic	orthorhombic	monoclinic	triclinic	monoclinic	orthorhombic
Space group	*Cc*	*P*2_1_2_1_2_1_	*P*2_1_2_1_2_1_	*P*2_1_/*n*	*P*-1	*P*2_1_/*c*	*Pnma*
a/Å	12.3081(13)	7.6390(4)	7.6198(5)	9.5389(10)	8.5540(11)	8.4238(4)	14.4316(7)
b/Å	10.1119(13)	12.0399(6)	11.9637(8)	16.9155(17)	13.2482(17)	13.5803(6)	8.9132(5)
c/Å	11.2029(12)	18.6843(10)	18.6306(14)	14.7768(14)	17.920(2)	18.6065(8)	19.5405(10)
α/°	90	90	90	90	107.492(2)	90	90
β/°	96.578(2)	90	90	91.479(4)	95.600(2)	98.507(2)	90
γ/°	90	90	90	90	97.994(2)	90	90
Volume/Å^3^	1385.1(3)	1718.45(15)	1698.4(2)	2383.5(4)	1897.0(4)	2105.12(16)	2513.5(2)
Z	4	4	4	4	4	4	4
ρ_calc_/gcm^−3^	1.554	1.462	1.483	1.717	1.416	1.434	1.311
μ/mm^−1^	1.324	1.078	1.202	3.350	0.982	0.894	0.831
F(000)	676.0	796.0	800.0	1216.0	852.0	956.0	1048.0
Crystal size/mm^3^	0.355 × 0.247 × 0.142	0.213 × 0.15 × 0.078	0.287 × 0.134 × 0.127	0.05 × 0.02 × 0.01	0.253 × 0.154 × 0.05	0.253 × 0.169 × 0.078	0.216 × 0.169 × 0.088
Radiation/Å	MoKα, 0.71073	MoKα, 0.71073	MoKα, 0.71073	Synchrotron, 0.7749	MoKα, 0.71073	MoKα, 0.71073	MoKα, 0.71073
2Θ range for data collection/°	5.228 to 56.576	4.024 to 56.618	4.046 to 56.798	3.992 to 52.228	2.41 to 56.584	3.728 to 56.632	3.508 to 56.726
Index ranges	−16 ≤ h ≤ 14 −13 ≤ k ≤ 13 −14 ≤ l ≤ 14	−10 ≤ h ≤ 10 −16 ≤ k ≤ 16 −24 ≤ l ≤ 24	−10 ≤ h ≤ 9 −15 ≤ k ≤ 15 −24 ≤ l ≤ 24	−10 ≤ h ≤ 10 −19 ≤ k ≤ 19 −16 ≤ l ≤ 16	−11 ≤ h ≤ 11 −17 ≤ k ≤ 17 −23 ≤ l ≤ 23	−11 ≤ h ≤ 11 −18 ≤ k ≤ 18 −24 ≤ l ≤ 24	−19 ≤ h ≤ 19 −11 ≤ k ≤ 11 −26 ≤ l ≤ 26
Reflections collected	13204	31559	41891	37933	29491	49812	46814
Independent reflections	3268	4259	4239	3652	9415	5246	3344
R_int_	0.0178	0.0520	0.0400	0.1904	0.0370	0.0340	0.0444
Data/restraints/parameters	3268/2/167	4259/0/192	4239/0/192	3652/0/269	9415/41/437	5246/0/244	3344/235/201
Goodness-of-fit on F^2^	1.042	1.180	1.064	1.037	1.005	1.089	1.031
Final R indexes [I ≥ 2σ (I)]	R_1_ = 0.0131 wR_2_ = 0.0319	R_1_ = 0.0400 wR_2_ = 0.0740	R_1_ = 0.0385 wR_2_ = 0.0920	R_1_ = 0.0617 wR_2_ = 0.1579	R_1_ = 0.0307 wR_2_ = 0.0591	R_1_ = 0.0264 wR_2_ = 0.0603	R_1_ = 0.0284 wR_2_ = 0.0652
Final R indexes [all data]	R_1_ = 0.0133 wR_2_ = 0.0320	R_1_ = 0.0445 wR_2_ = 0.0751	R_1_ = 0.0401 wR_2_ = 0.0931	R_1_ = 0.0820 wR_2_ = 0.1757	R_1_ = 0.0499 wR_2_ = 0.0645	R_1_ = 0.0294 wR_2_ = 0.0615	R_1_ = 0.0372 wR_2_ = 0.0693
Largest diff. peak/hole/e Å^−3^	0.18/−0.12	0.37/−0.52	0.93/−0.57	1.03/−0.74	0.33/−0.51	0.39/−0.22	0.30/−0.23
Flack parameter	0.077(10)	0.32(3) *(racemic twin)	0.47(3) *(racemic twin)	none	none	none	none

**Table 2:** Details of selected bond lengths and angles.

Structure	M-N1/Å M-N3/Å	M-N2/Å M-N4/Å	N1-M-N3/° N2-M-N4/°
[Mn(**L1**)Cl_2_]	2.2761(14) 2.2935(13)	2.3255(14) 2.3041(13)	142.60(5) 73.49(5)
[Mn(**L3**)Cl_2_]	2.392(3) 2.313(3)	2.302(3) 2.314(3)	142.48(10) 74.85(10)
[Fe(**L3**)Cl_2_]	2.320(3) 2.257(3)	2.235(4) 2.233(4)	146.15(12) 77.20(11)
[Fe(**L5**)(OAc)_2_] Ref: [50]	2.3024(10) 2.3024(9)	2.2091(10) 2.2228(11)	148.84(4) 77.91(4)
[Mn(**L9**)Cl_2_]	**Mn1:** 2.3378(13) 2.3242(14) **Mn2:** 2.3510(14) 2.3786(15)	**Mn1:** 2.2706(14) 2.2981(13) **Mn2:** 2.3138(14) 2.2873(14)	**Mn1:** 144.69(5) 74.86(5) **Mn2:** 143.88(5) 74.15(5)
[Mn(**L11**)Cl_2_]	2.4019(11) 2.3935(11)	2.3090(11) 2.2834(11)	143.56(4) 74.09(4)
[Fe(**L11**)Cl_2_] ˑ 2MeCN	2.2705(15) 2.262(2)	2.278(3) 2.169(3)	148.85(7) 77.38(12)

**Table 3:** Kinetic stability of [Cu**L**Cl_2_] and [Cu**L**Cl]PF_6_ complexes of ligands **L1**–**L10** under harsh acidic conditions.

Ligand	Pseudo-First-Order Half-Life for Decomplexation of 1.000 mM Copper Complexes					
	30 °C 1 M HCl	30 °C 5 M HCl	50 °C 5 M HCl	70 °C 5 M HCl	90 °C 5 M HCl	References
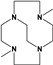 **Me_2_Bcyclen**	36 min		<1 min			[55]
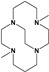 **Me_2_EBC**			7.3 day		79 min	[22,55]
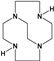 **H_2_Bcyclen (L1)**	<1 min					[21]
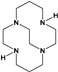 **H_2_Bcyclam (L2)**	18.5 d		5.40 h [This work]		11.8 min	[54]
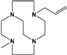 **Me_1_Allyl_1_Bcyclen (L3)**	25 min					This work
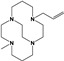 **Me_1_Allyl_1_Bcyclam (L4)**			2.33 h			This work
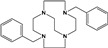 **Bn_2_Bcyclen (L5)**	4.2 h	7.8 min				[33,56]
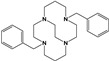 **Bn_2_Bcylam (L6)**			2.38 h	23.4 min		[33,56]
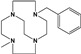 **Me_1_Bn_1_Bcyclen (L7)**	24.8 min					This work
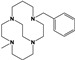 **Me_1_Bn_1_Bcyclam (L8)**			15.05 h	1.4 h		This work
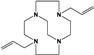 **Allyl_2_Bcyclen (L9)**	51.7 min					This work
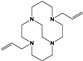 **Allyl_2_Bcyclam (L10)**			1.86 h	15 min		This work

**Table 4:** Redox potentials (V) vs. SHE (0.00 V) and ∆V (mV) for 1.000 mM solutions of the Fe and Mn complexes of **L1**–**L12** in MeCN.

Complex	E_1/2_ M^2+/3+^ [V] (∆V [mV])	E_1/2_ M^3+/4+^ [V] (∆V [mV])	Reference
Fe(**Me_2_Bcyclen**)Cl_2_	+0.036 (64)	-----	[12]
Mn(**Me_2_Bcyclen**)Cl_2_	+0.466 (70)	+1.232 (102)	[12]
Fe(**Me_2_Bcyclam**)Cl_2_	+0.110 (63)	-----	[12]
Mn(**Me_2_Bcyclam**)Cl_2_	+0.585 (61)	1.343 (65)	[12]
[Fe**L1**Cl_2_]	-0.055 (89)	-----	[13]
[Mn**L1**Cl_2_]	+0.389 (280)	not accessible in DMF	[13]
[Fe**L2**Cl_2_]	-0.113 (78)	-----	[13]
[Mn**L2**Cl_2_]	+0.239 (79)	not accessible in DMF	[13]
[Fe**L3**Cl_2_]	+0.094 (82)	-----	This work
[Mn**L3**Cl_2_]	+0.545 (71)	+1.338 (86)	This work
[Fe**L4**Cl2]	+0.165 (96)	-----	This work
[Mn**L4**Cl_2_]	+0.628 (59)	+1.374 (83)	This work
[Fe**L5**Cl_2_]	+0.071 (85)	-----	[13]
[Mn**L5**Cl_2_]	+0.400 (65)	not accessible in DMF	[13]
[Fe**L6**Cl_2_]	+0.157 (85)	-----	[13]
[Mn**L6**Cl_2_]	+0.577 (72)	not accessible in DMF	[13]
[Fe**L7**Cl_2_]	+0.065 (64)	-----	This work
[Mn**L7**Cl_2_]	+0.550 (76)	+1.355 (82)	This work
[Fe**L8**Cl_2_]	+0.176 (86)	-----	This work
[Mn**L8**Cl_2_]	+0.609 (93)	+1.404 (92)	This work
[Fe**L9**Cl_2_]	+0.114 (92)	-----	This work
[Mn**L9**Cl_2_]	+0.572 (68)	+1.385 (79)	This work
[Fe**L10**Cl_2_]	+0.194 (59)	-----	This work
[Mn**L10**Cl_2_]	+0.616 (72)	+1.422 (83)	This work
[Fe**L11**Cl_2_]	+0.130 (78)	-----	This work
[Mn**L11**Cl_2_]	+0.558 (72)	+1.390 (73)	This work
[Fe**L12**Cl_2_]	+0.196 (72)	-----	This work
[Mn**L12**Cl_2_]	+0.619 (85)	+1.433 (92)	This work

**Table 5:** Turnover frequency (TOF) values, and other relevant parameters, for control and all 24 metal–complex catalysts for degradation of MB, MO, and RhB.

Catalyst	Average TOF for MB (h^−1^) (Rank among Top 8)	Average TOF for MO (h^−1^) (Rank among Top 8)	Average TOF for RhB (h^−1^) (Rank among Top 8)	Catalyst Rank Based on Average Rank Order for Top 8 Catalysts for All Three Dye TOFs	Highest Redox Potential Observed (Fe^3+^, Mn^3+^, or Mn^4+^)	Half-Life of Cu^2+^ Complex Under Acid Conditions (Cyclen Complexes in 1 M HCl, 30 °C; Cyclam Complexes in 5 M HCl, 50 °C)
H_2_O_2_ only	0.018	0.034	0.002			
FeCl_2_ only	0.111	0.926	0.055			
MnCl_2_ only	0.003	0.518	0.047			
[Fe**L1**Cl_2_]	0.54	1.33	0.31		−0.055 V	<1 min
[Mn**L1**Cl_2_]	0.32	1.08	0.14		+0.389 V	<1 min
[Fe**L2**Cl_2_]	1.21 (7)	31.35 (3)	3.62 (1)	**3 (tie)** **(Avg = 3.67)**	−0.113 V	5.40 h
[Mn**L2**Cl_2_]	0.27	0.147	0.18		+0.239 V	5.40 h
[Fe**L3**Cl_2_]	0.33	1.46	0.64		+0.94 V	25 min
[Mn**L3**Cl_2_]	1.67 (5)	6.42 (8)	1.104 (7)	**6** **(Avg = 6.67)**	+1.338 V	25 min
[Fe**L4**Cl2]	0.35	1.26	0.63		+0.165 V	2.33 h
[Mn**L4**Cl_2_]	2.03 (4)	16.26 (4)	1.05 (8)	**4** **(Avg = 5.33)**	+1.374 V	2.33 h
[Fe**L5**Cl_2_]	0.13	4.08	0.21		+0.071 V	4.2 h
[Mn**L5**Cl_2_]	0.16	0.60	0.11		+0.400 V	4.2 h
[Fe**L6**Cl_2_]	0.45	3.41	0.62		+0.157 V	2.38 h
[Mn**L6**Cl_2_]	0.74 (8)	16.14 (5)	1.27 (5)	**5 (tie)** **(Avg = 6)**	+0.577 V	2.38 h
[Fe**L7**Cl_2_]	0.13	0.083	0.13		+0.065 V	24.8 min
[Mn**L7**Cl_2_]	2.64 (3)	8.32 (7)	2.34 (2)	**3 (tie)** **(Avg = 3.67)**	+1.355 V	24.8 min
[Fe**L8**Cl_2_]	0.57	2.82	1.54		+0.176 V	15.05 h
[Mn**L8**Cl_2_]	2.09 (3)	108.9 (1)	1.46 (4)	**2** **(Avg =2.67)**	+1.404 V	15.05 h
[Fe**L9**Cl_2_]	0.35	0.77	0.92		+0.114 V	51.7 min
[Mn**L9**Cl_2_]	0.53	3.13	0.35		+1.385 V	51.7 min
[Fe**L10**Cl_2_]	0.171	2.2	0.35		+0.194 V	1.86 h
[Mn**L10**Cl_2_]	1.23 (6)	9.40 (6)	1.16 (6)	**5 (tie)** **(Avg = 6)**	+1.422 V	1.86 h
[Fe**L11**Cl_2_]	0.19	0.43	0.26		+0.130 V	n/a
[Mn**L11**Cl_2_]	0.18	0.86	0.15		+1.390 V	n/a
[Fe**L12**Cl_2_]	0.25	1.26	0.70		+0.196 V	n/a
[Mn**L12**Cl_2_]	5.87 (1)	36.49 (2)	1.61 (3)	**1 (Avg = 2.0)**	+1.433 V	n/a

## Data Availability

Data presented in this study are available in the Supplementary Materials or upon request from the corresponding authors.

## Supplementary Materials

The following supporting information can be downloaded at: https://www.mdpi.com/article/10.3390/molecules28186466/s1. Figures S1–S12: ^1^H and ^13^C NMR spectra for novel organic compounds. Figure S13: Example of kinetic decomplexation plot to determine t_1/2_ for copper complexes. Figures S14–S21: Cyclic voltammograms for novel Mn and Fe complexes. Figures S22–S33: Dye bleaching plots of catalyst, H_2_O_2_, and dye (MB, MO, RhB). Tables S1–S52: Crystallographic details for all new structures [69,70,71].

## Sample Availability

Samples of the compounds are not available.
