# Supplementary material for: Earth Abundant Oxidation Catalysts for Removal of Contaminants of Emerging Concern from Wastewater: Homogeneous Catalytic Screening of Monomeric Complexes
Source: Molecules. 2023 Sep 6;28(18):6466. doi: 10.3390/molecules28186466 (PMC10536317; doi:10.3390/molecules28186466)
Supplement: Supplementary file 1 [file molecules-28-06466-s001.zip › molecules-2522663-supplementary.pdf]

Earth abundant oxidation catalysts for removal of contaminants of emerging concern from wastewater: homogeneous catalytic screening of monomeric complexes designed for eventual co-polymerization to form recyclable heterogeneous catalysts

Leslie Garcia[1], Makynna R. Koper[1], Somrita Mondal[1], Joshua T. Priddle[1], William A. Truong[1], Elisabeth M. A. Allbritton[1], Ashtyn G. McAdoo[1], Desiray J. Cannon-Smith[1], Neil L. Funwie[1], Tuyet Hoang[1], Inseo Kim[1], David J. Hubin[1], Jeanette A. Krause[2], Allen G. Oliver[3], Timothy J. Prior[4], and Timothy J. Hubin[1,*]

Supplementary Information

Table of Contents **Page**

1. Figures S1-S12. ^{1}H and ^{13}C NMR Spectra for novel organic compounds. 2
2. Figure S13. Example of Kinetic Decomplexation Plot to Determine $t_{1/2}$ for Copper Complexes. 14
3. Figures S14-S21. Cyclic Voltammograms for novel Mn and Fe complexes 15
4. Figures S22-S33. Dye Bleaching Plots of catalyst, H_2O_2, and dye (MB, MO, RhB) 23
5. Tables S1-S52. Crystallographic Details for all new structures. 29

Figure S1. a) ^{1}H NMR (a) and ^{13}C NMR (b) spectra for **3**.

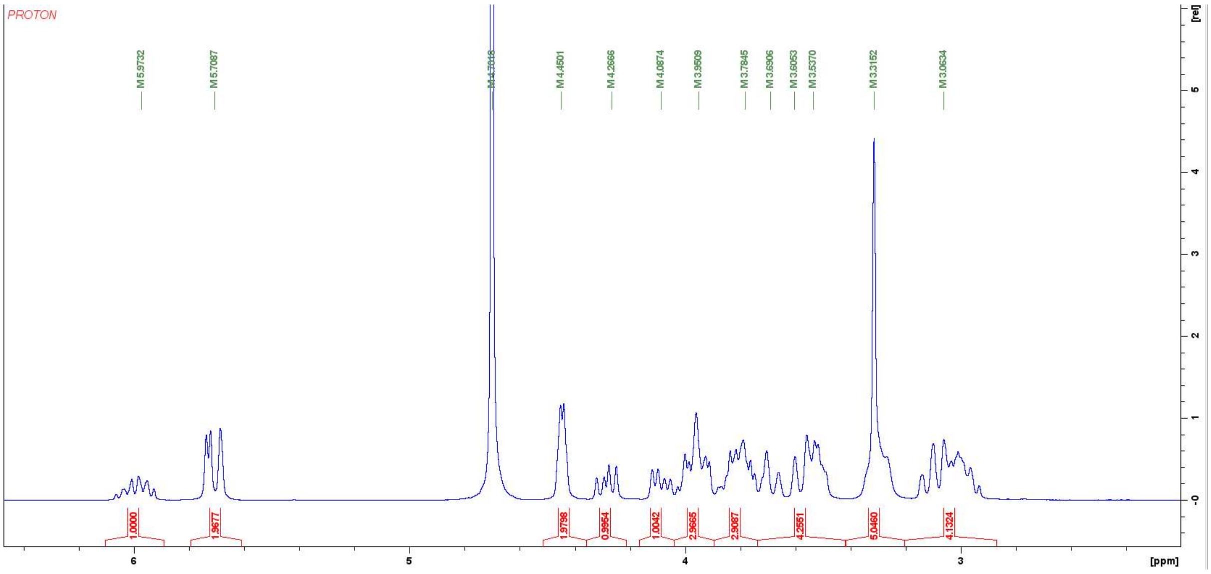

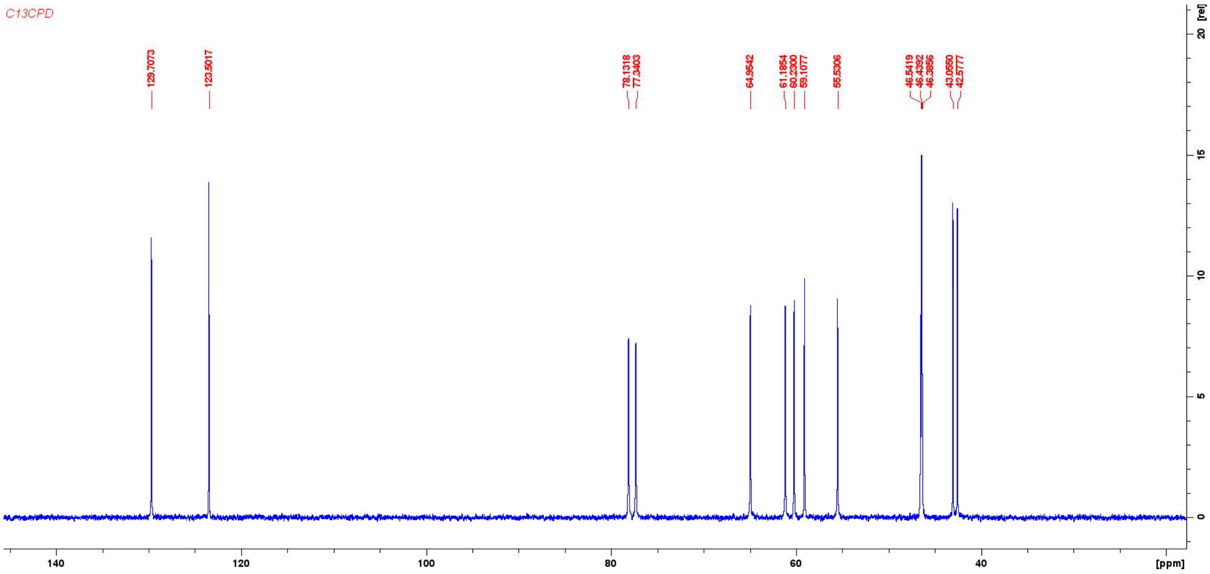

Figure S2. a) ^{1}H NMR (a) and ^{13}C NMR (b) spectra for **4**.

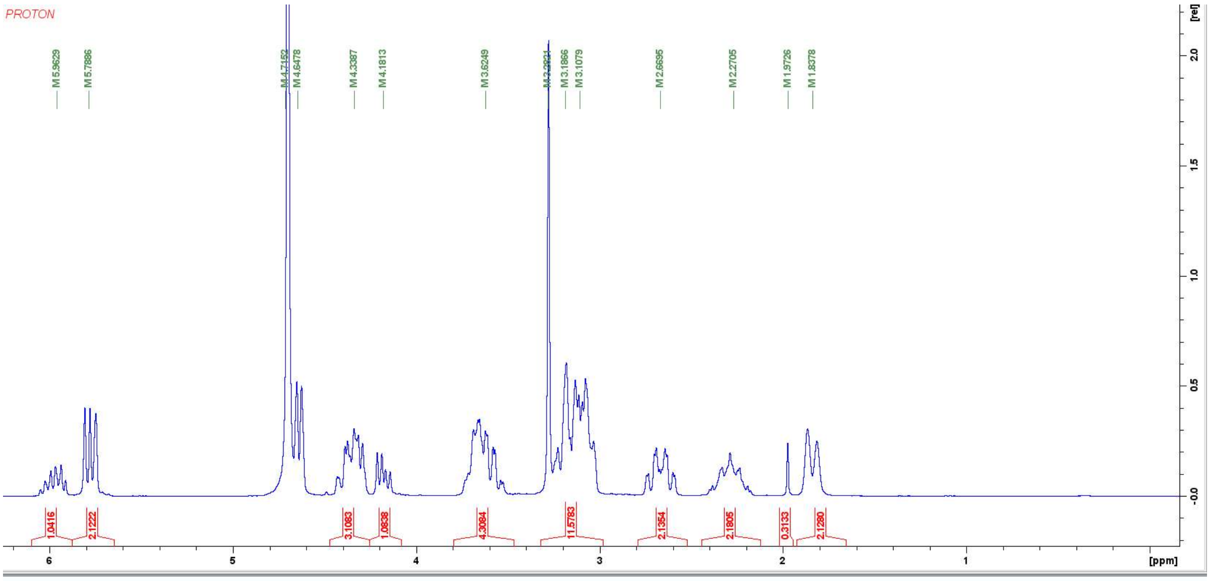

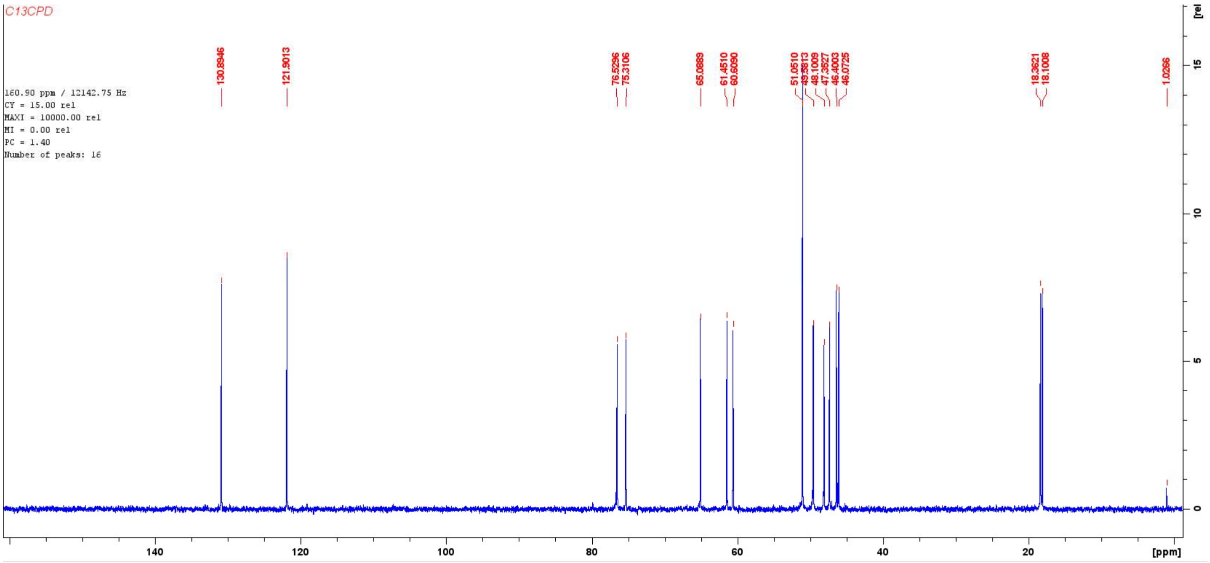

Figure S3. a) ^{1}H NMR (a) and ^{13}C NMR (b) spectra for **L3**.

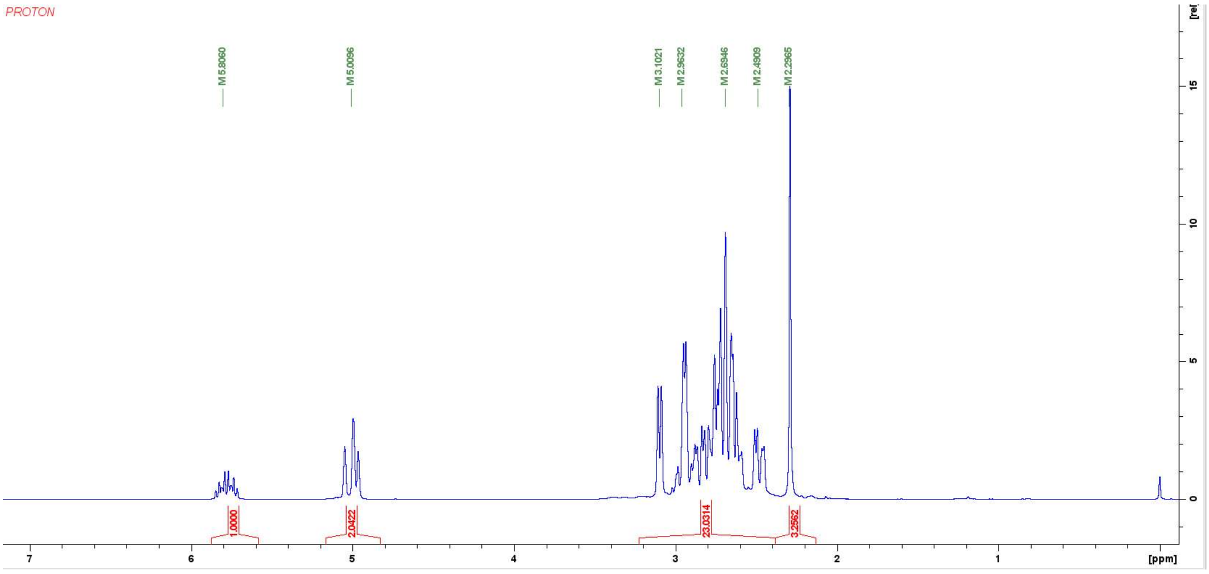

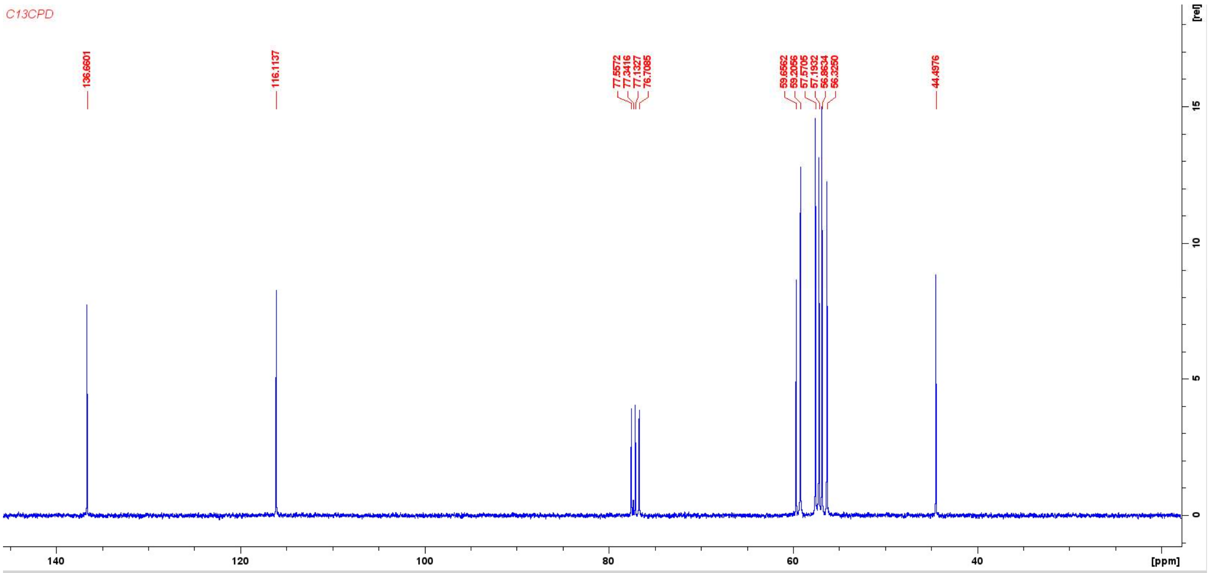

Figure S4. a) ^{1}H NMR (a) and ^{13}C NMR (b) spectra for **L4**.

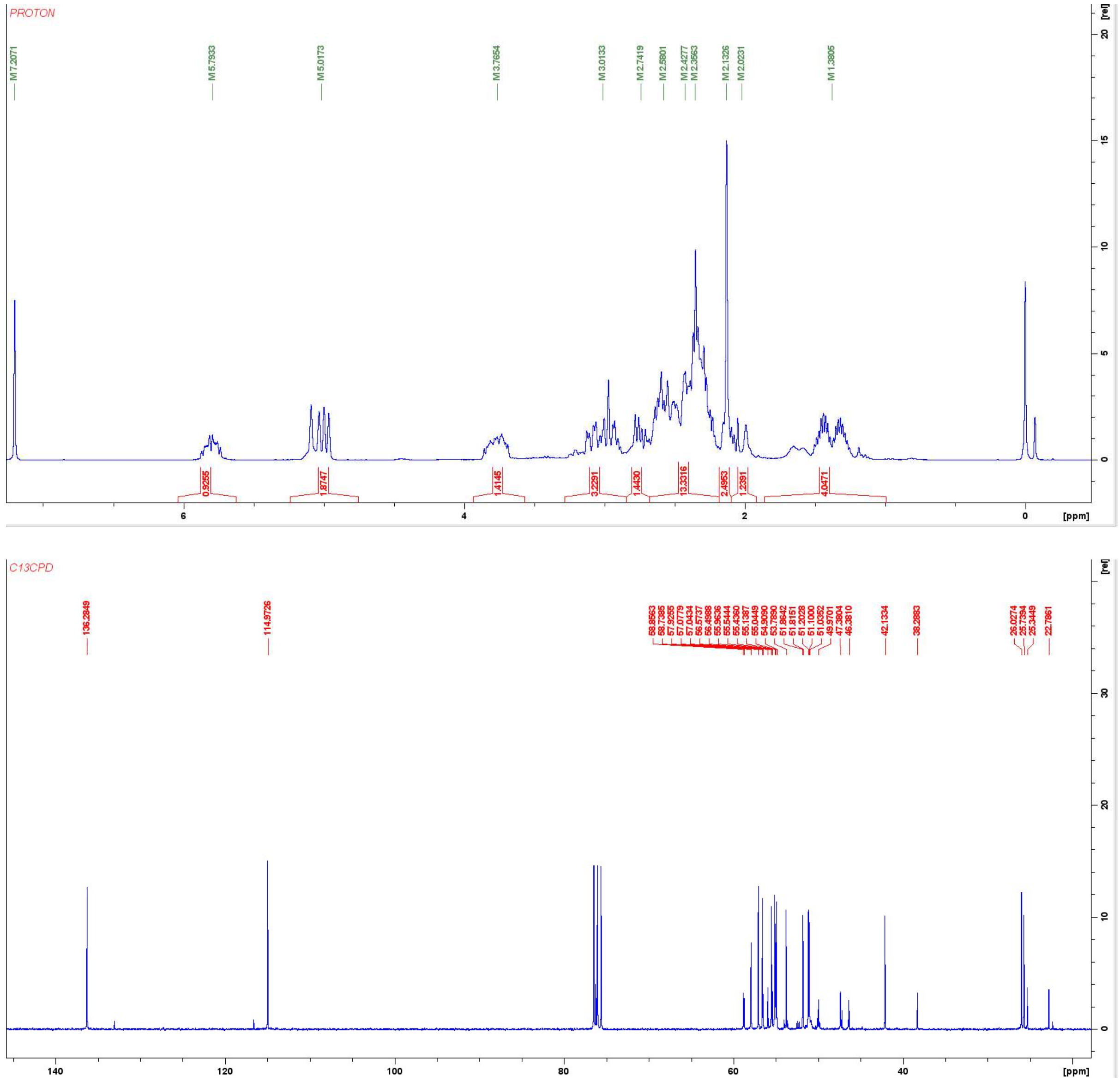

Figure S5. a) ^{1}H NMR (a) and ^{13}C NMR (b) spectra for **7**.

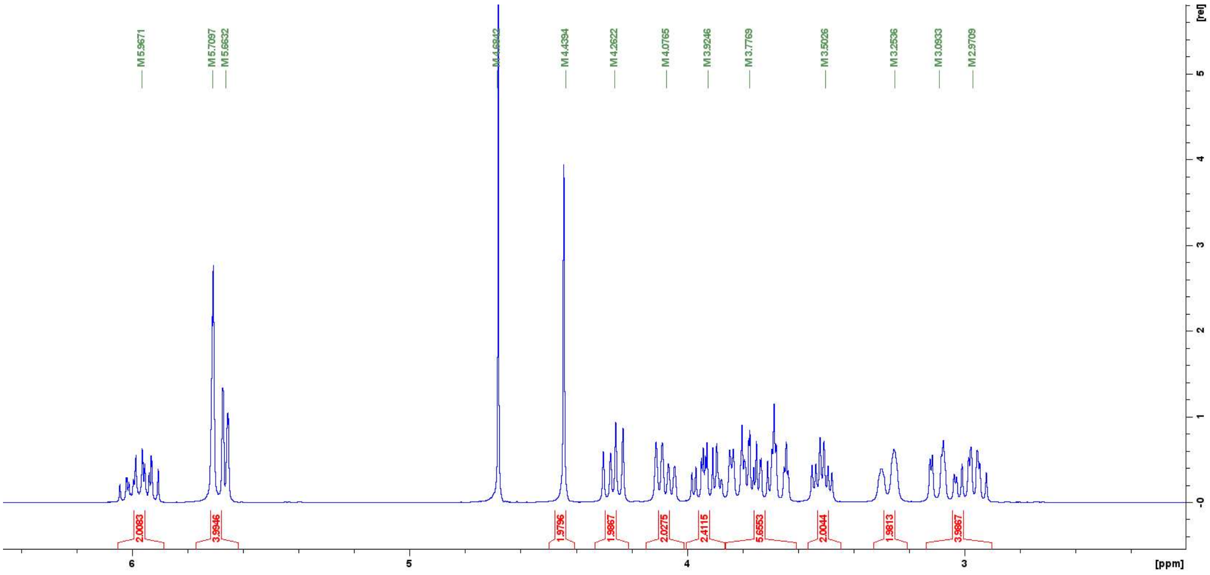

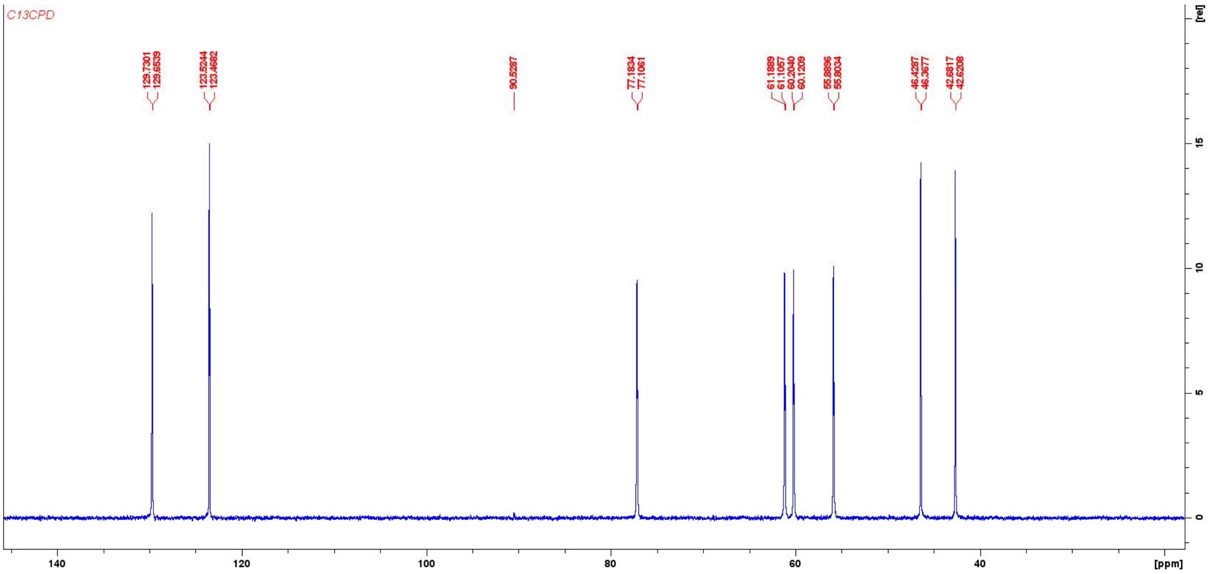

Figure S6. a) ^{1}H NMR (a) and ^{13}C NMR (b) spectra for **8**.

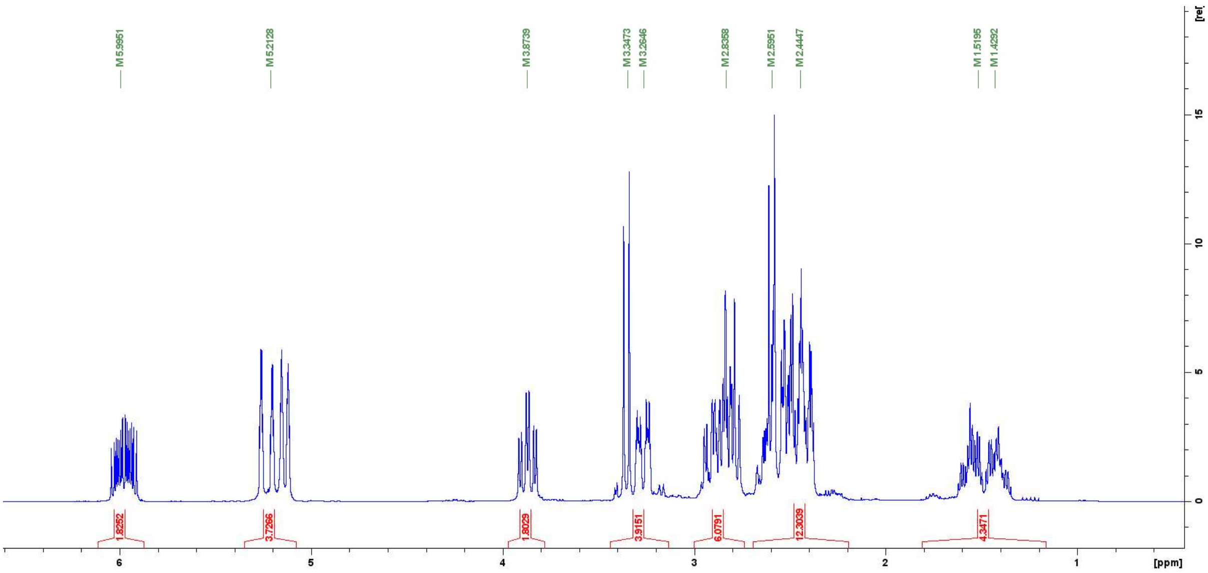

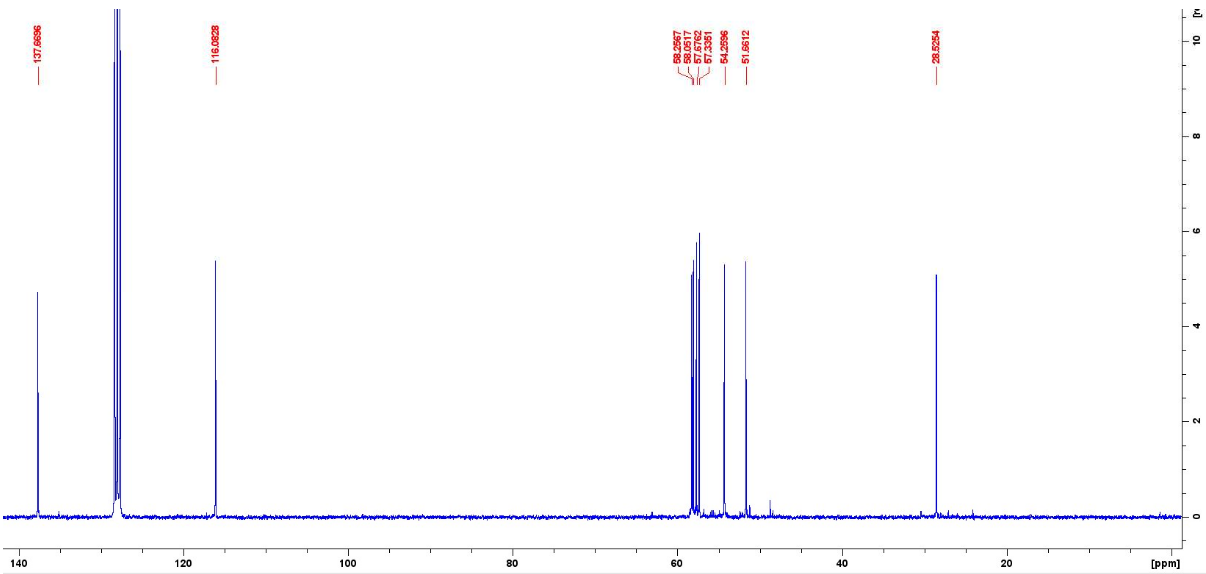

Figure S7. a) ^{1}H NMR (a) and ^{13}C NMR (b) spectra for **L9**.

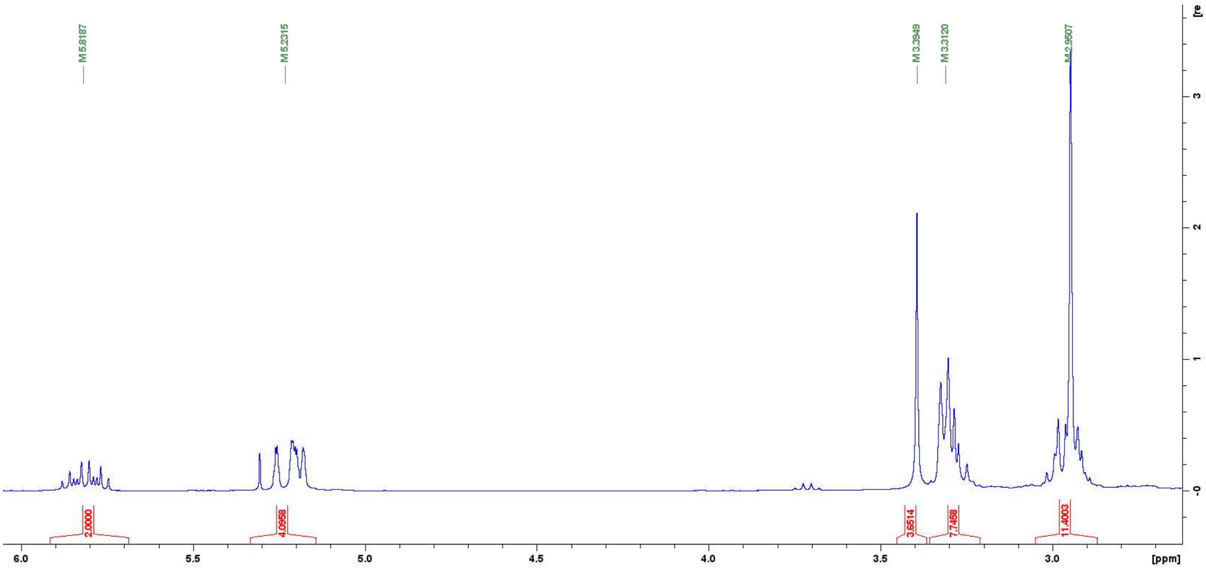

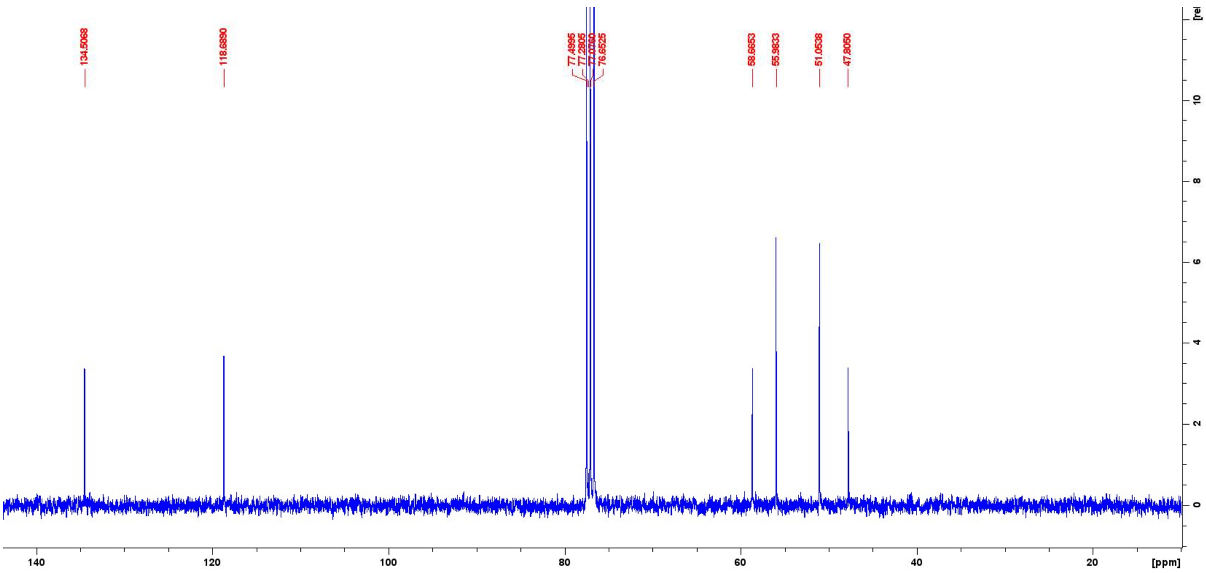

Figure S8. a) ^{1}H NMR (a) and ^{13}C NMR (b) spectra for **L10**.

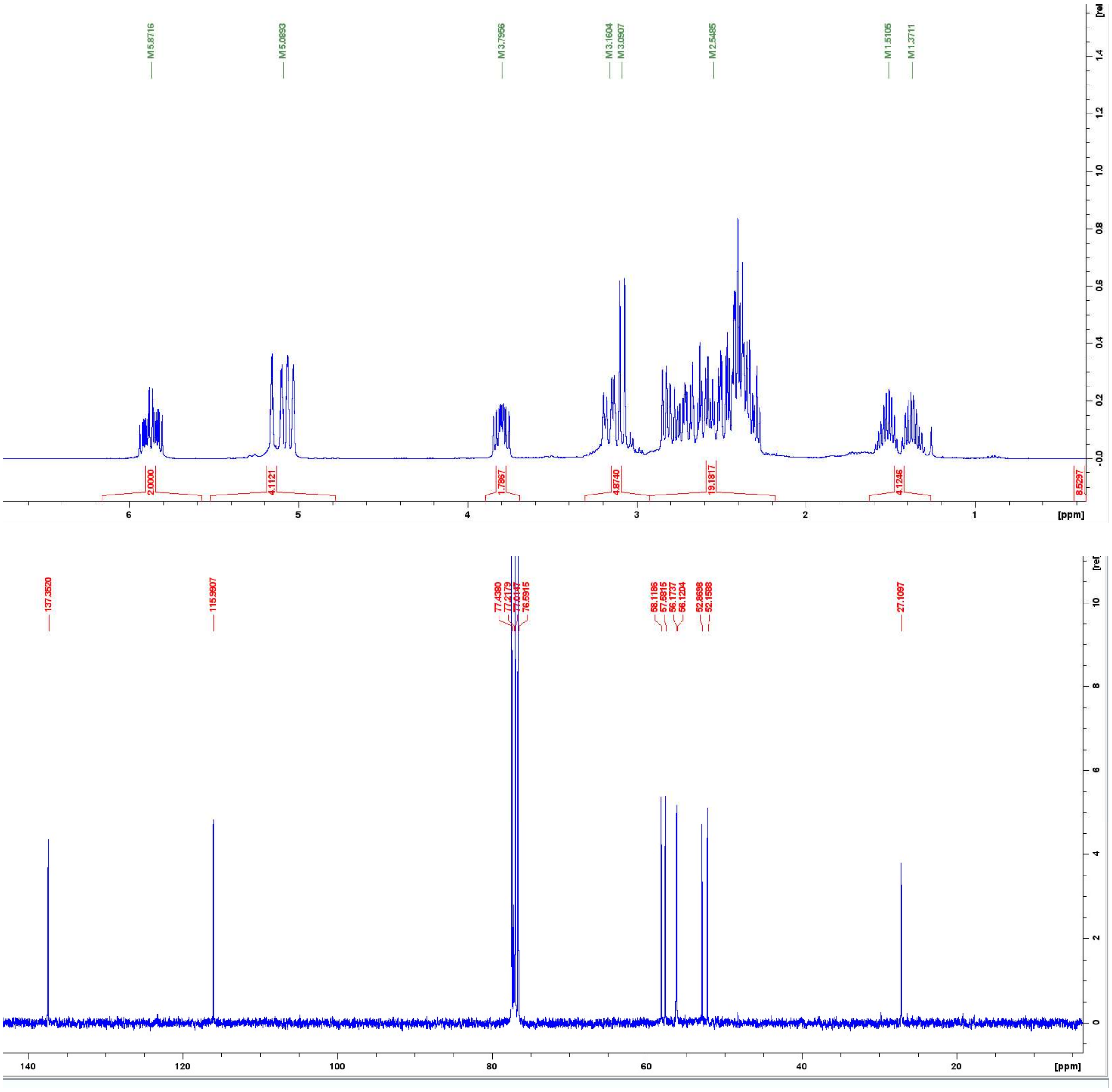

Figure S9. a) ^{1}H NMR (a) and ^{13}C NMR (b) spectra for **11**.

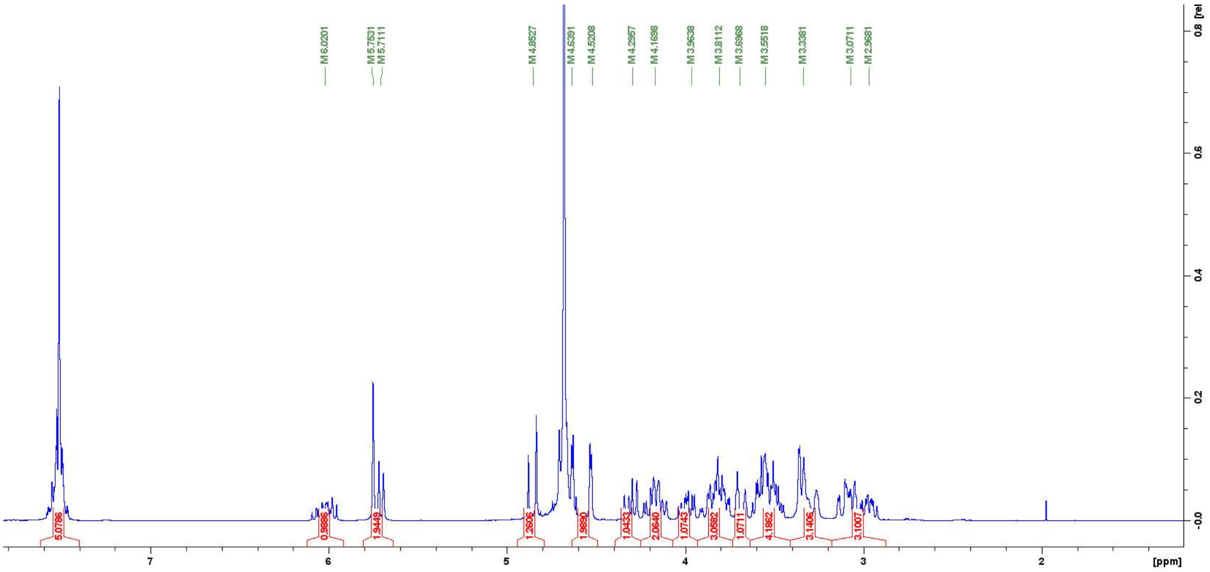

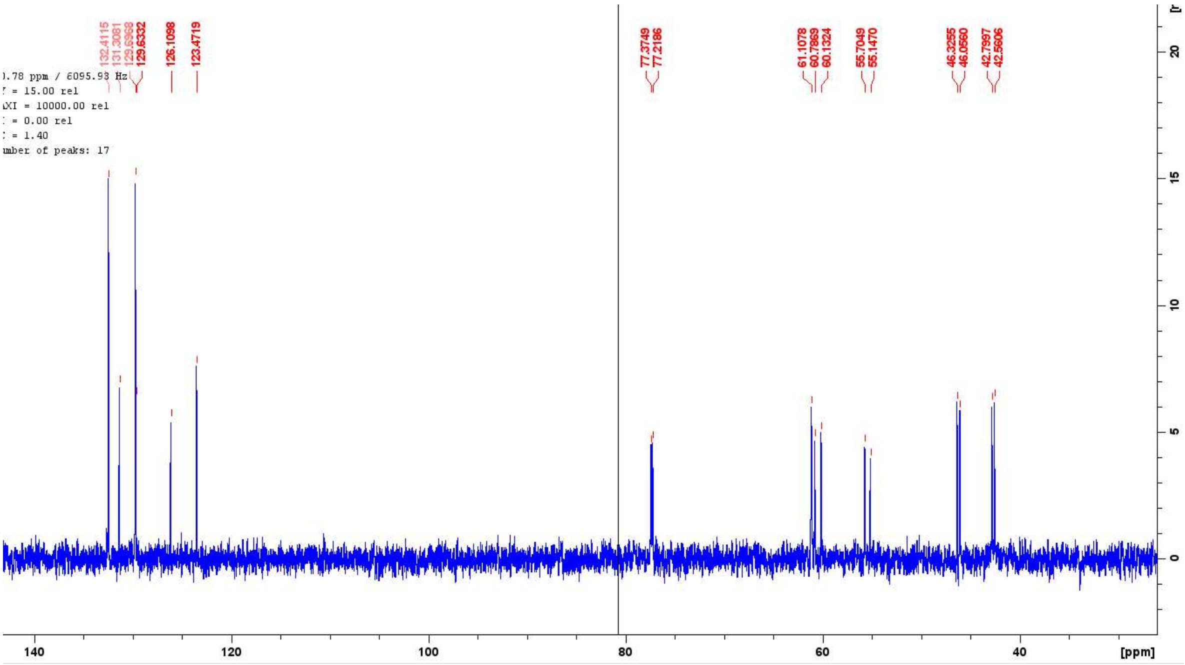

Figure S10. a) ^{1}H NMR (a) and ^{13}C NMR (b) spectra for **12**.

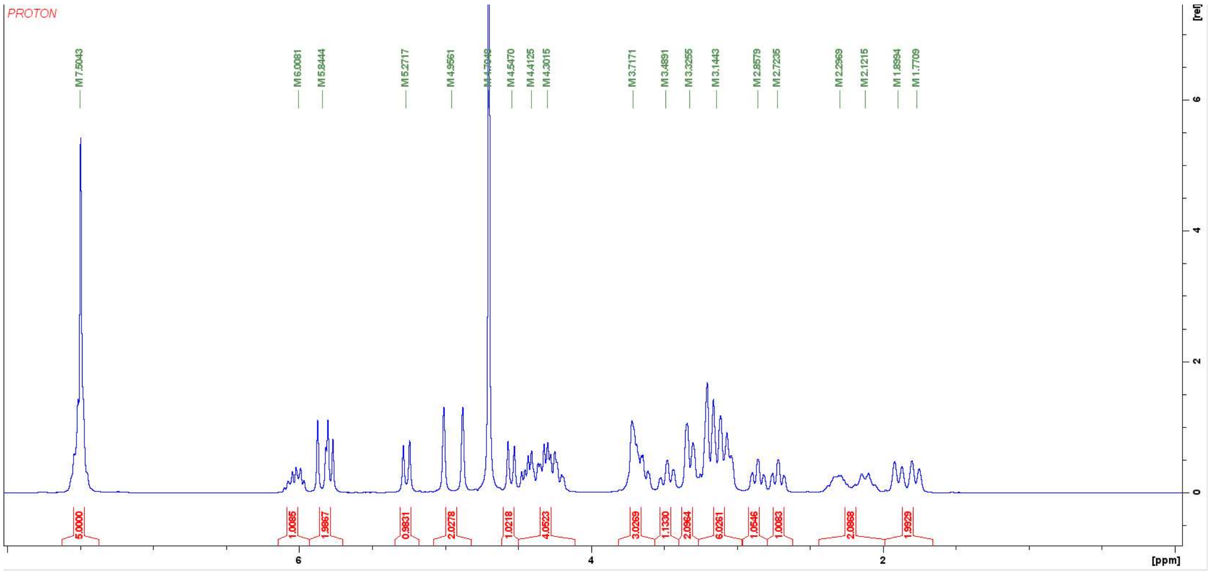

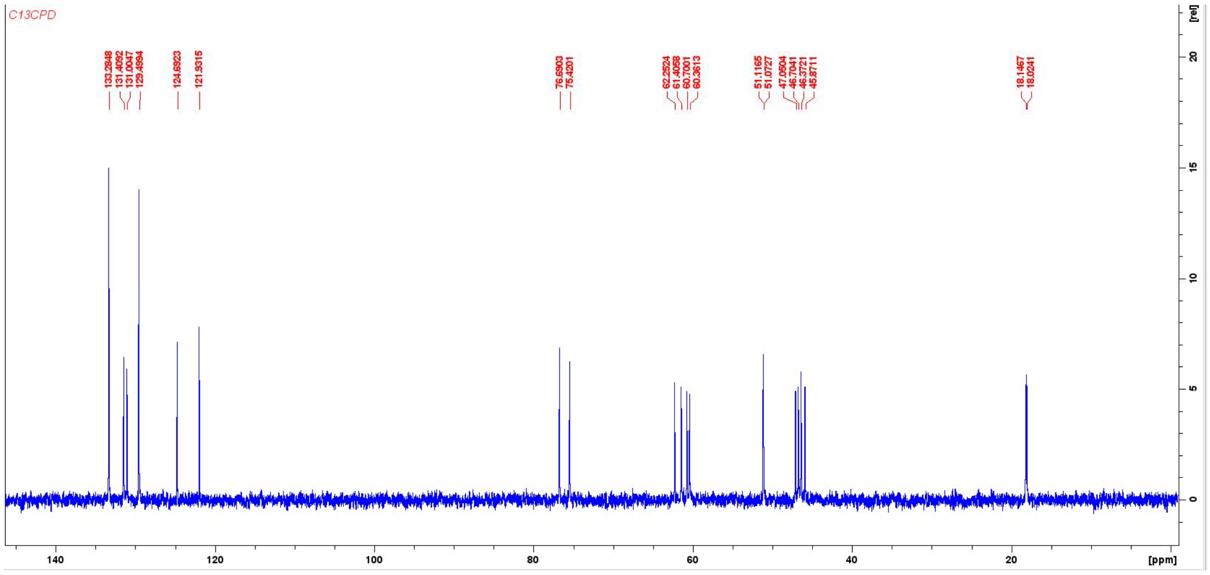

Figure S11. a) ^{1}H NMR (a) and ^{13}C NMR (b) spectra for **L11**.

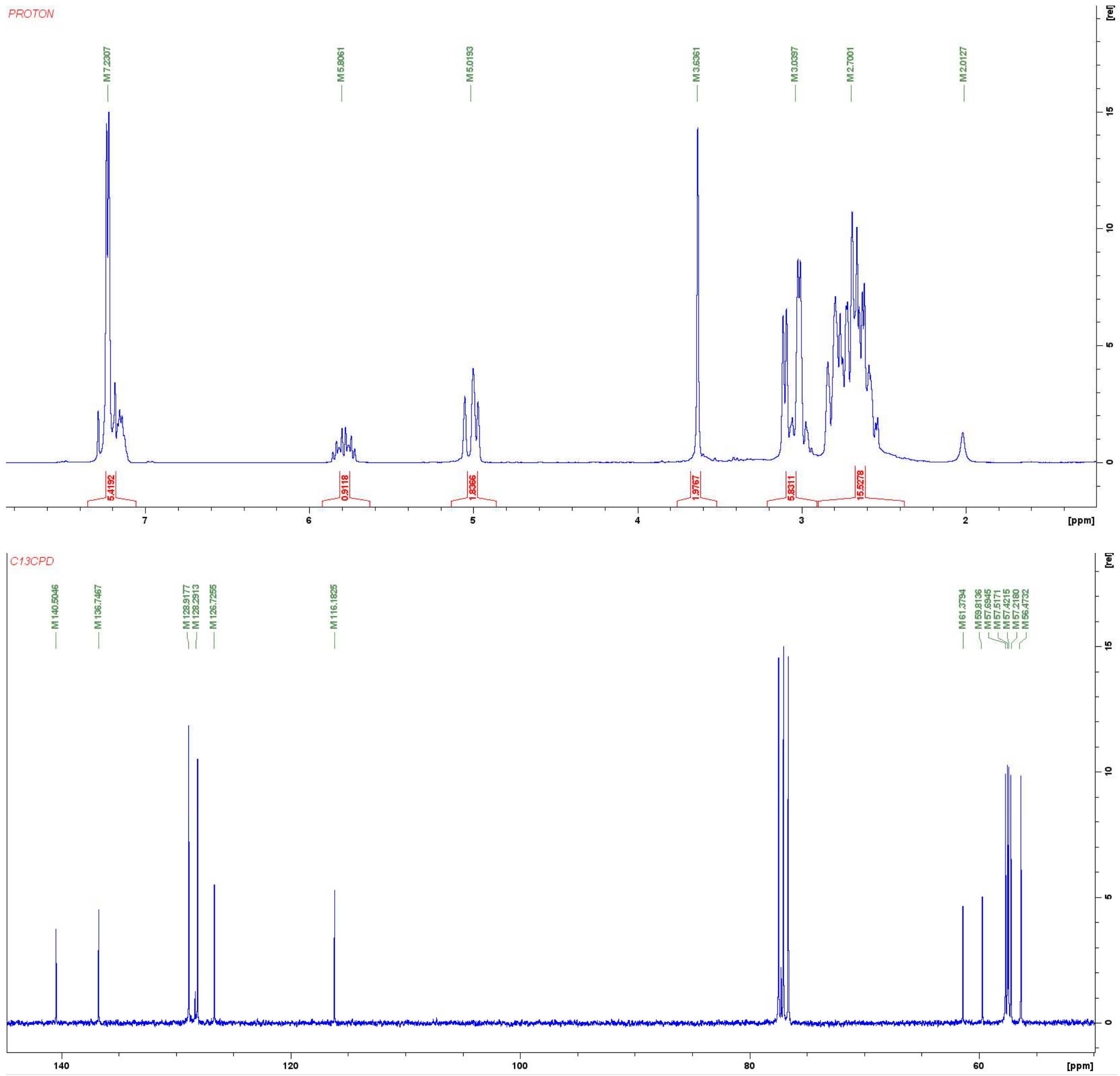

Figure S12. a) ^{1}H NMR (a) and ^{13}C NMR (b) spectra for **L12**.

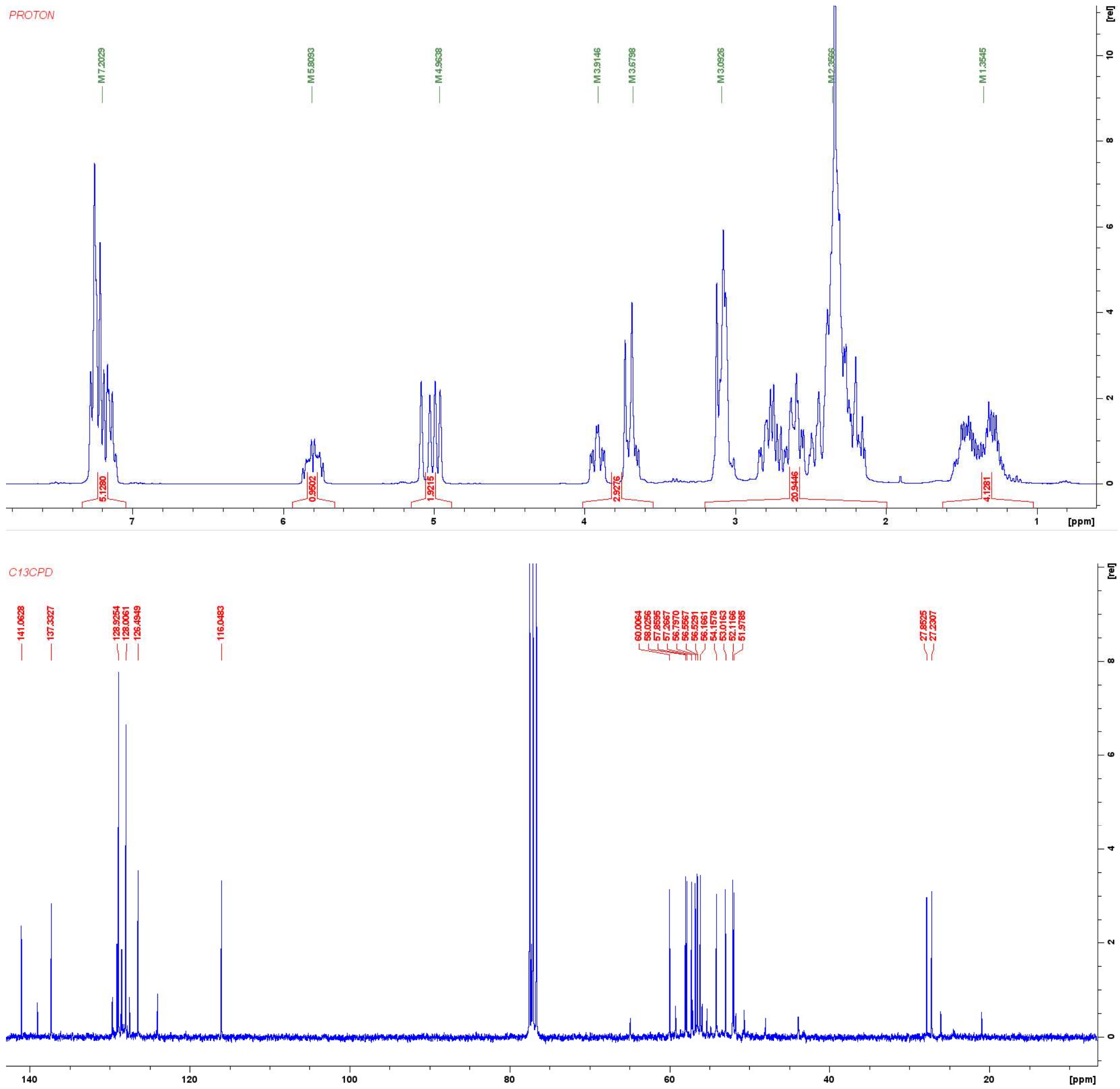

Figure S13. Example of Kinetic Decomplexation Plot to Determine $t_{1/2}$ for Copper Complexes. [Cu**(H_2Bcyclam)**Cl]PF_6 decomplexation in 5M HCl at 50 °C.

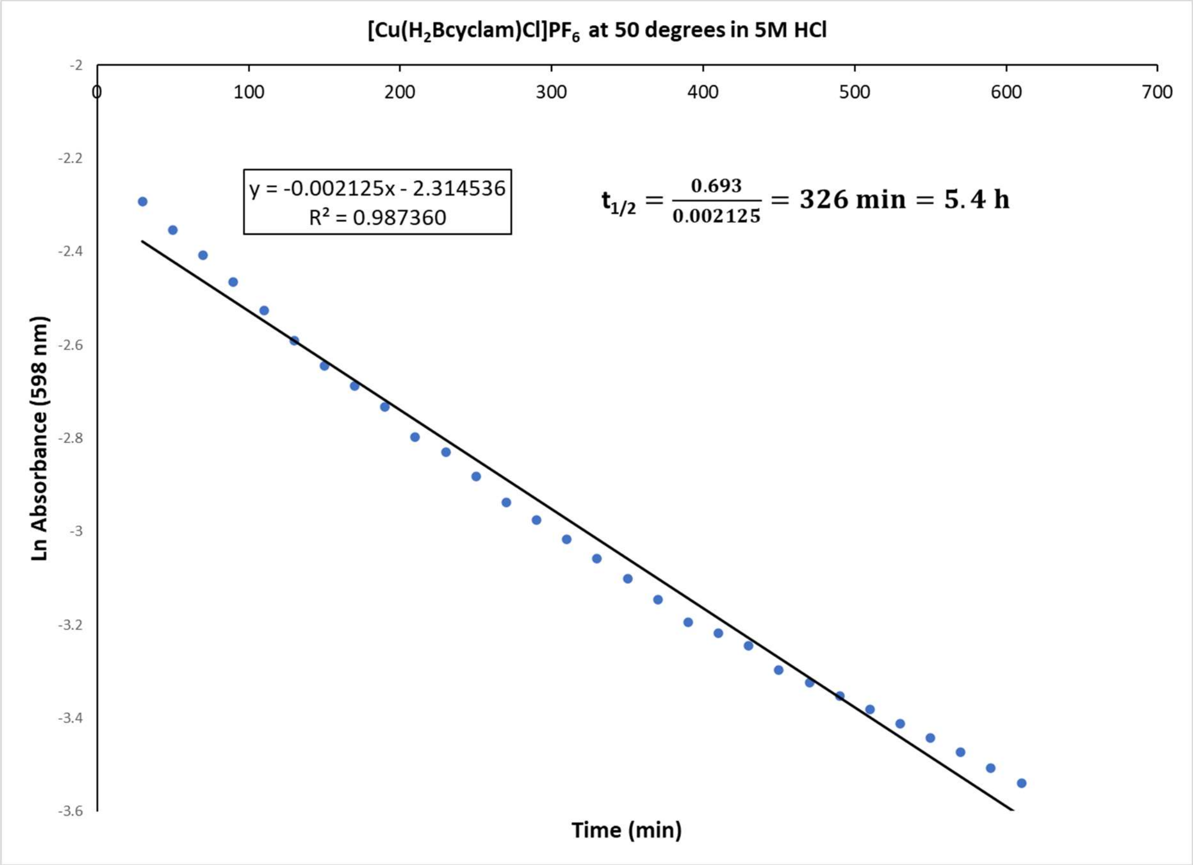

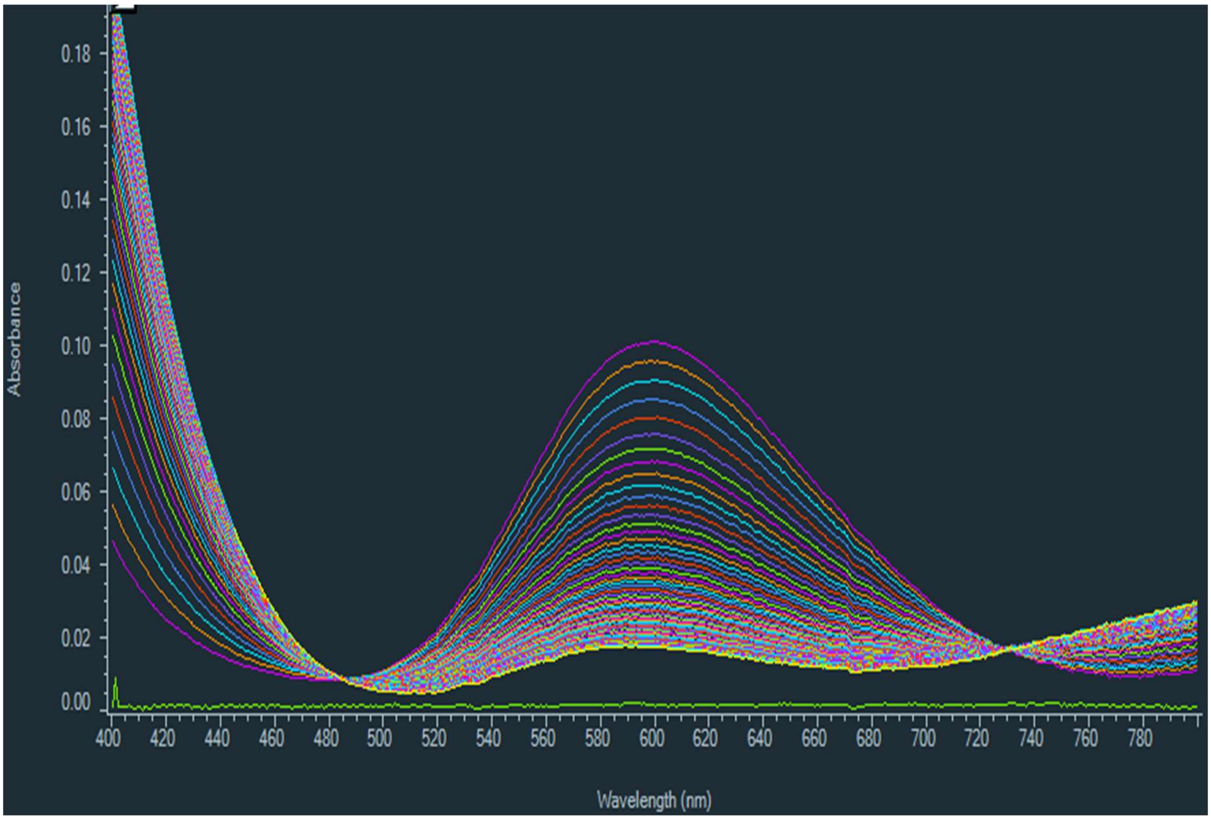

Figure S14. Cyclic Voltammograms in MeCN for (a) Mn**L3**Cl_2 and (b) Fe**L3**Cl_2.

(a)

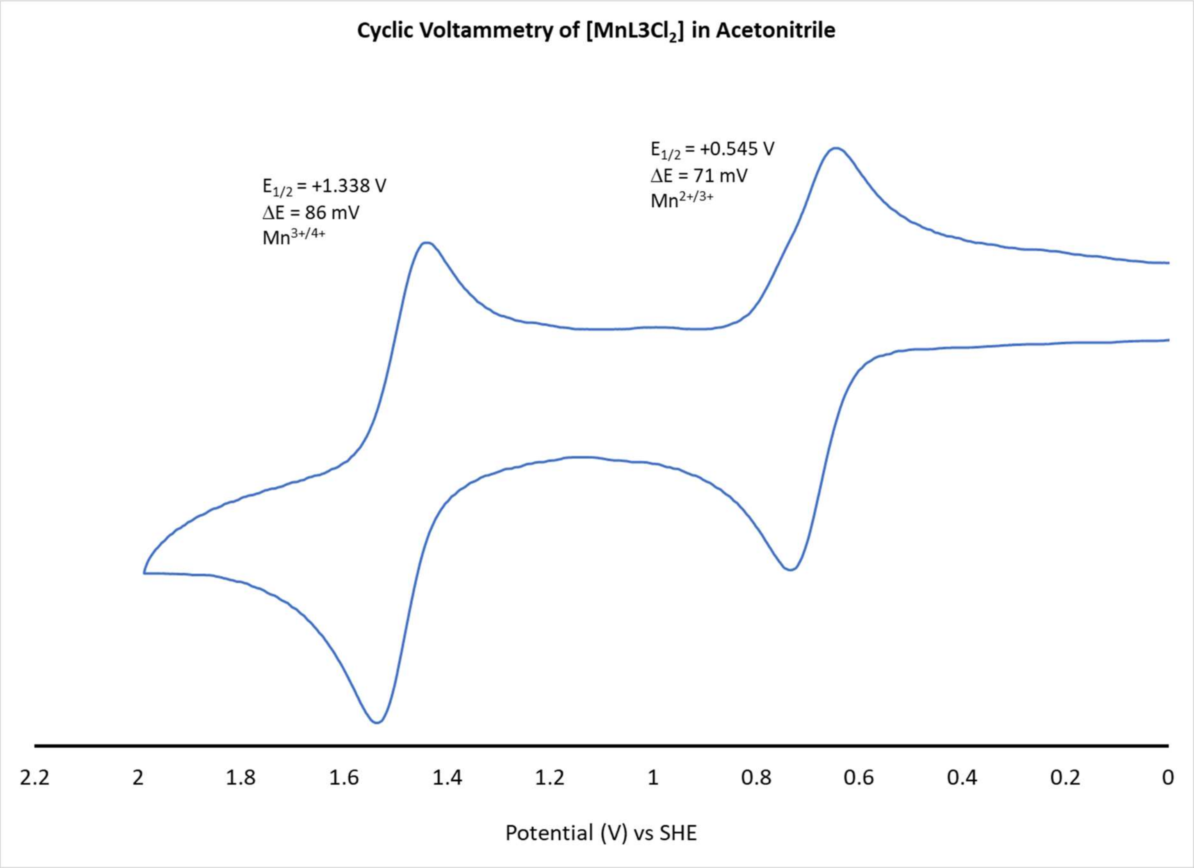

(b)

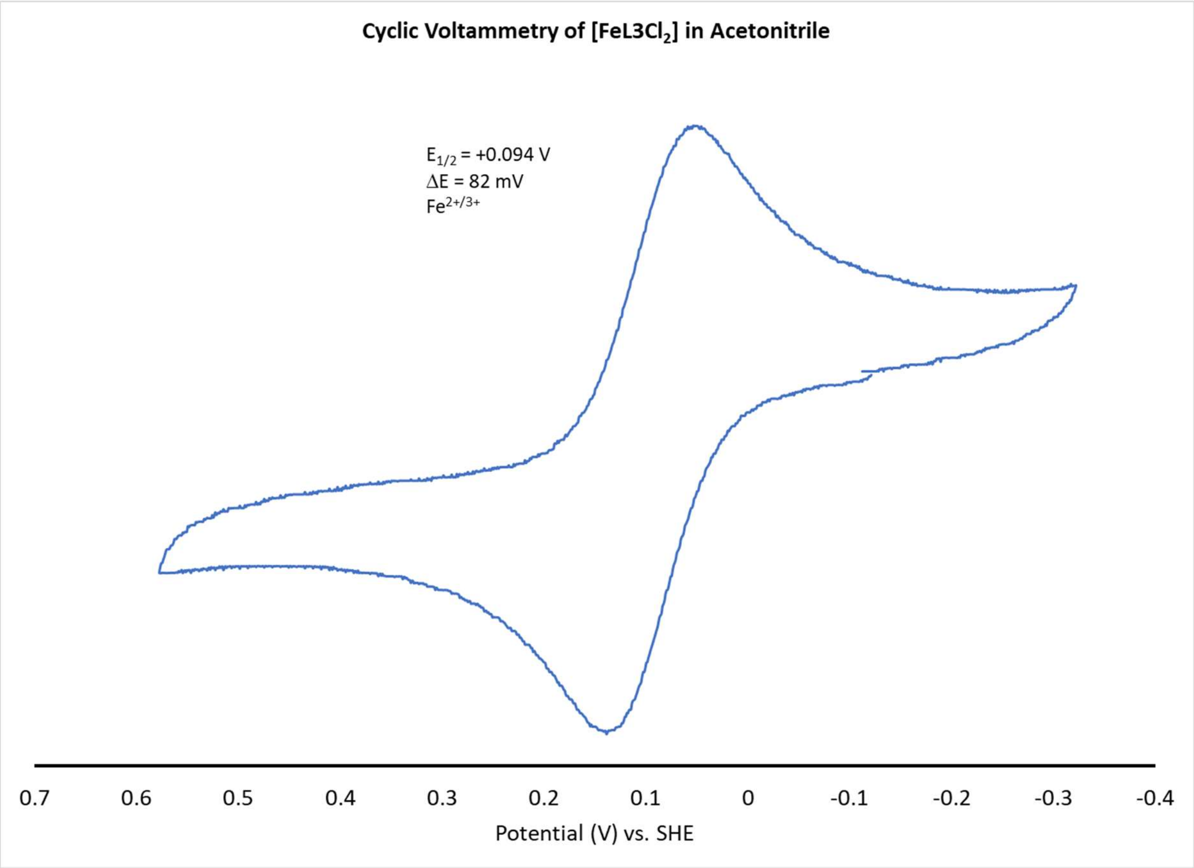

Figure S15. Cyclic Voltammograms in MeCN for (a) Mn**L4**Cl_2 and (b) Fe**L4**Cl_2.

(a)

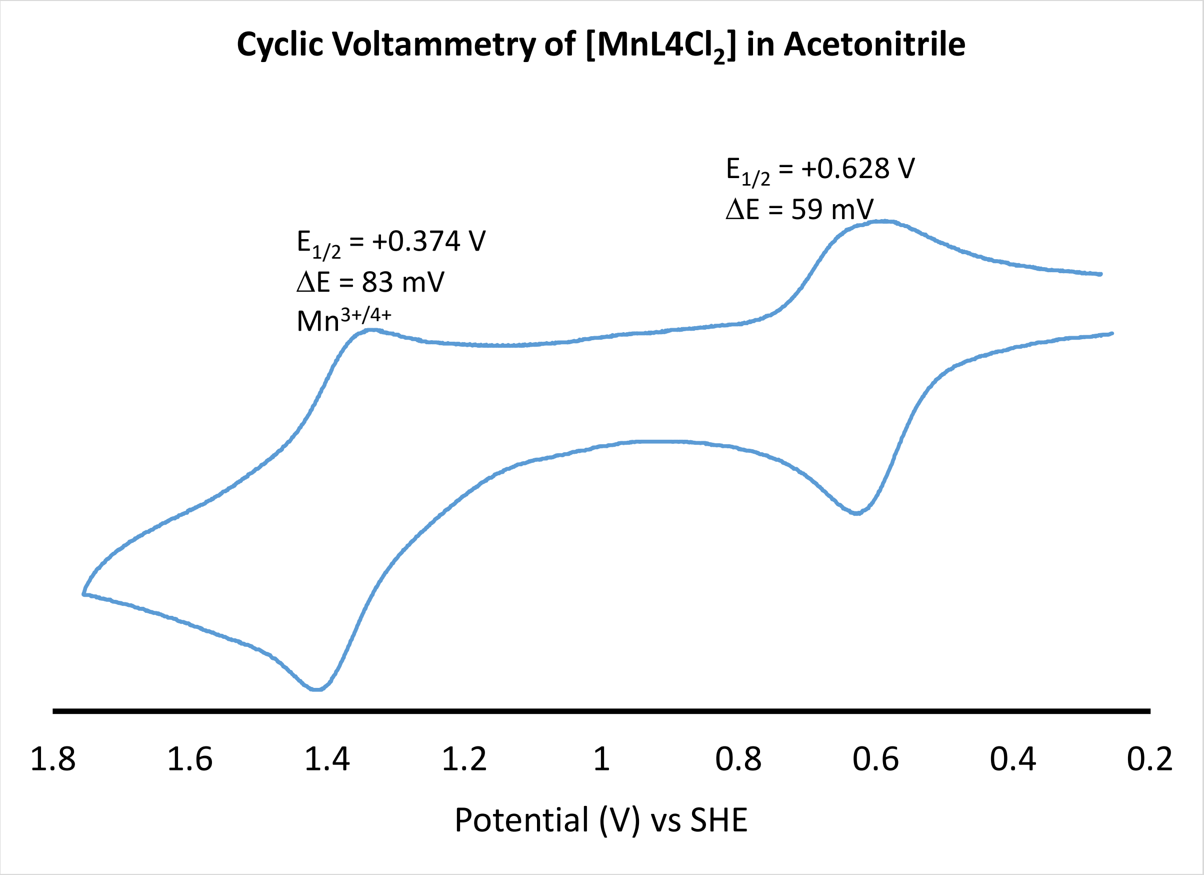

(b)

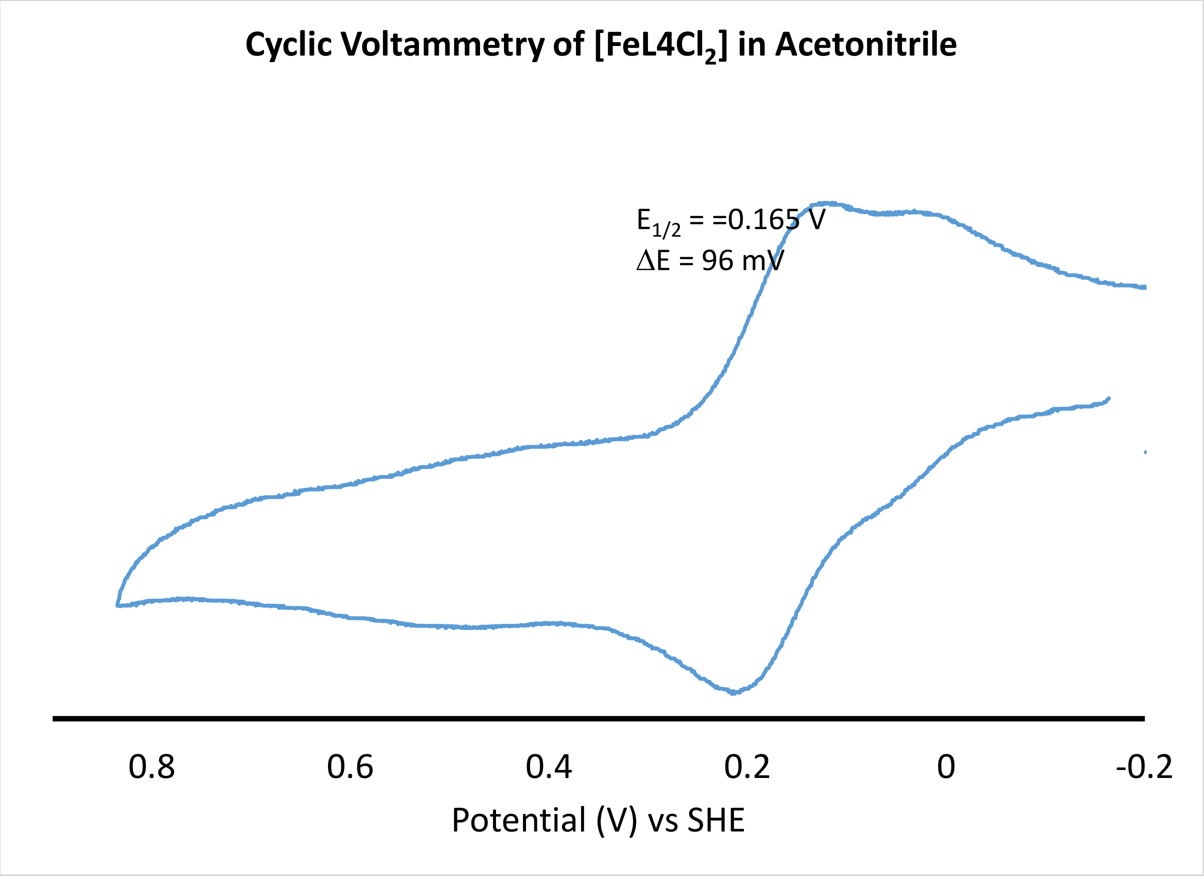

Figure S16. Cyclic Voltammograms in MeCN for (a) Mn**L7**Cl_2 and (b) Fe**L7**Cl_2.

(a)

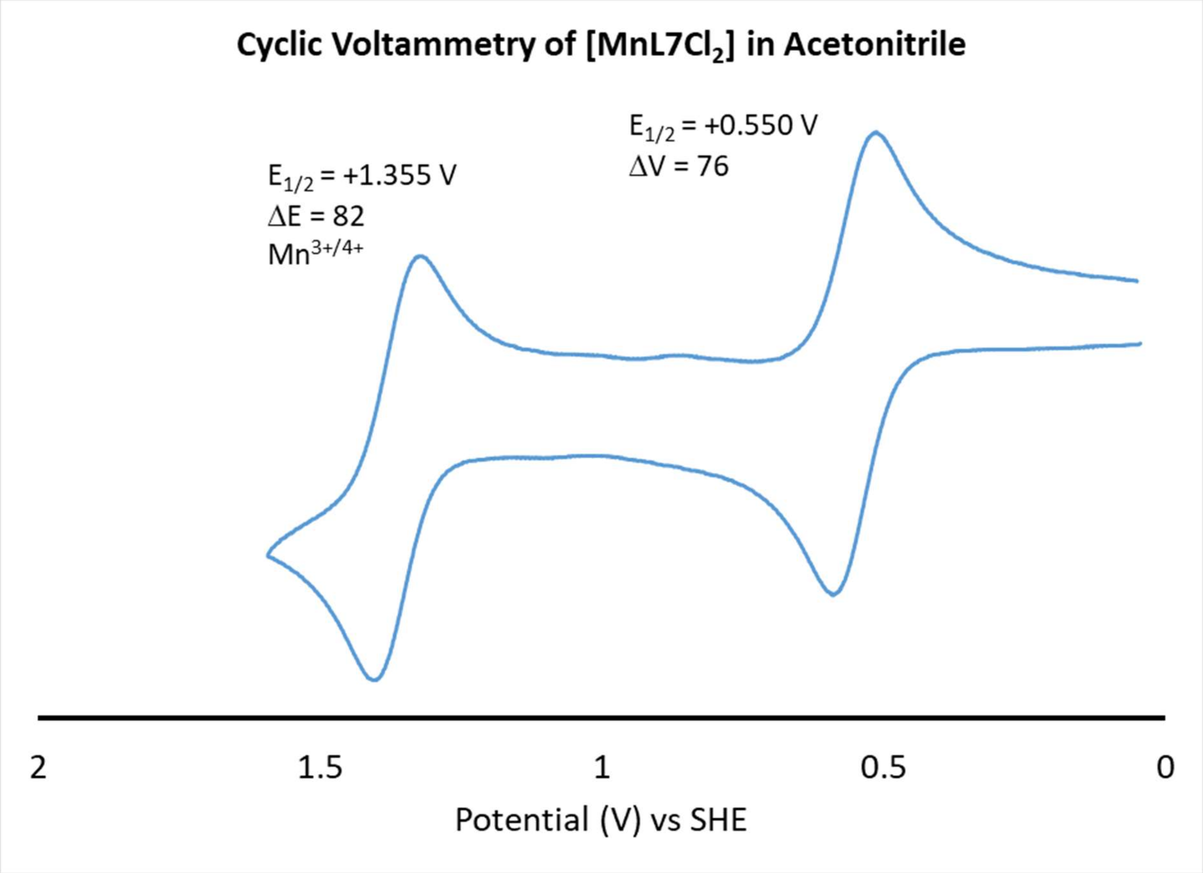

(b)

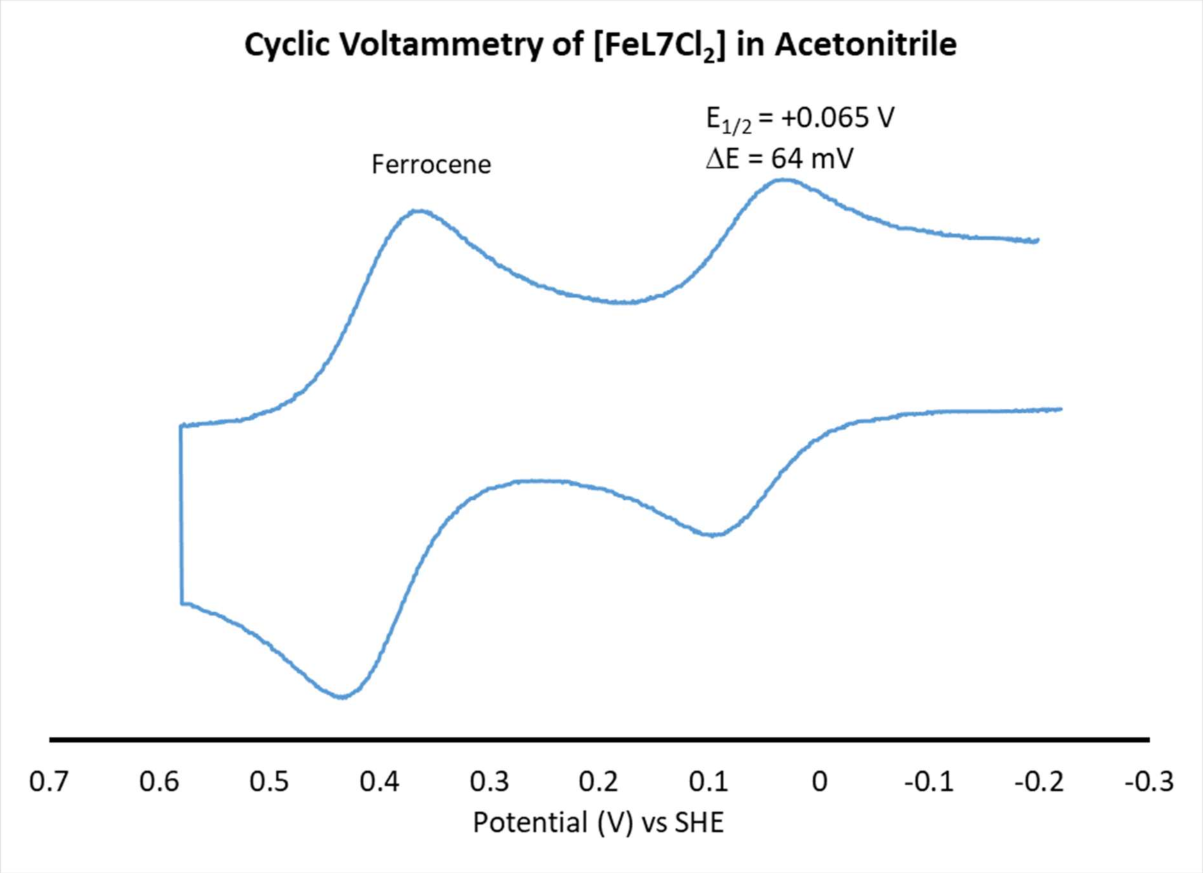

Figure S17. Cyclic Voltammograms in MeCN for (a) Mn**L8**Cl_2 and (b) Fe**L8**Cl_2.

(a)

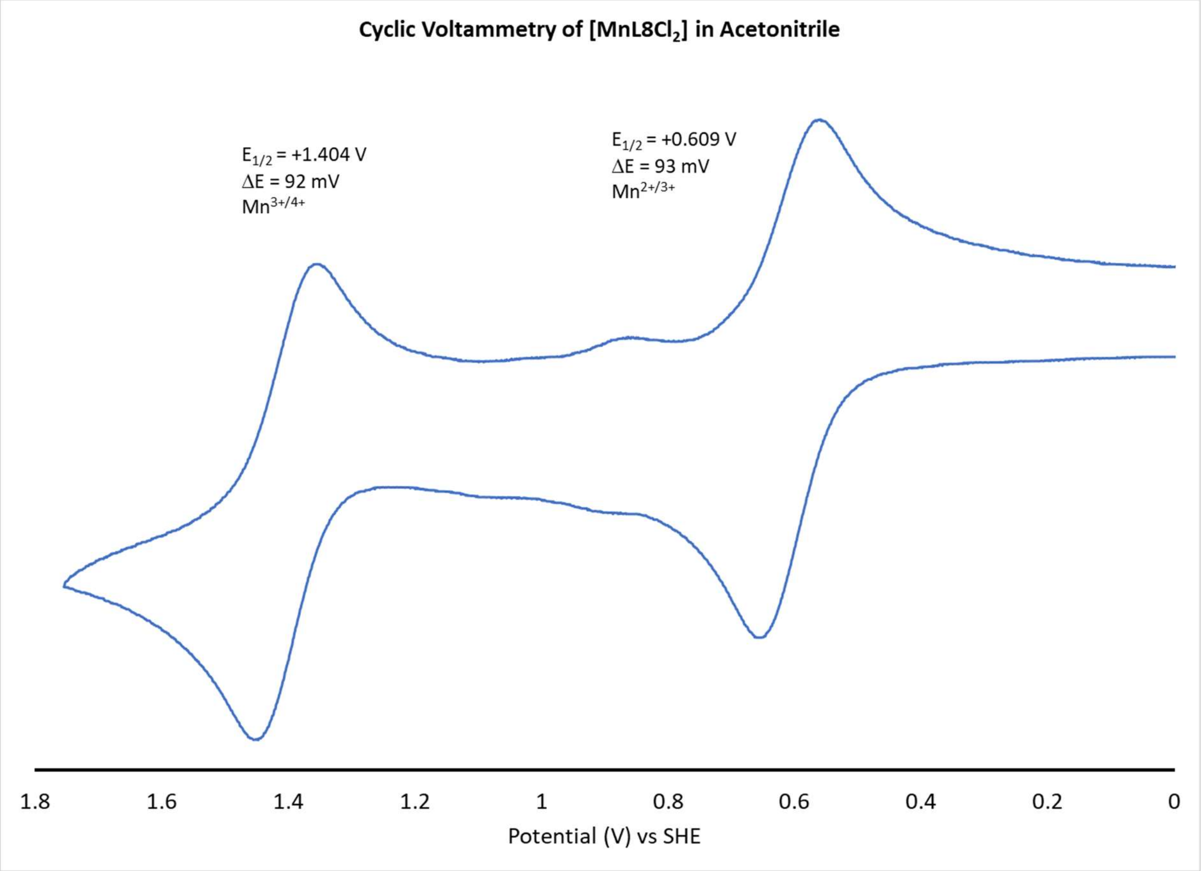

(b)

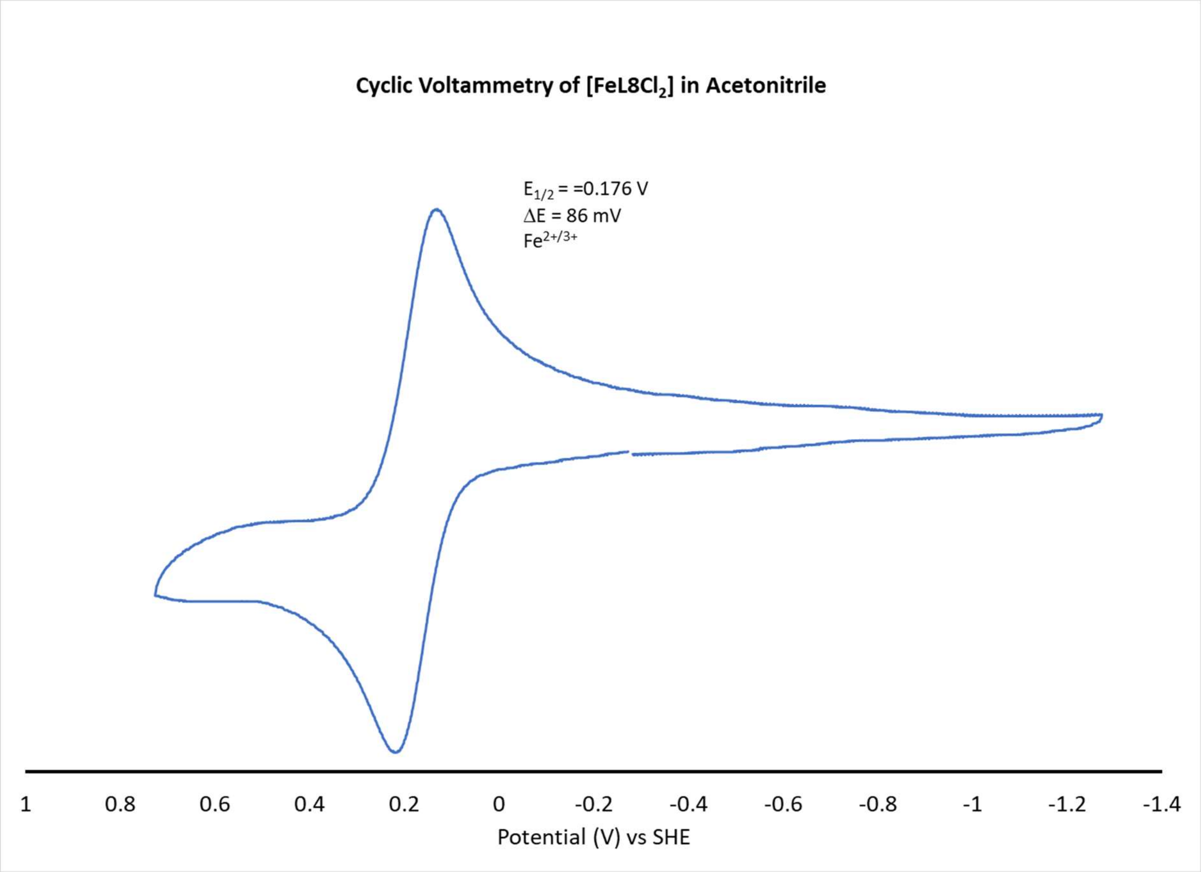

Figure S18. Cyclic Voltammograms in MeCN for (a) Mn**L9**Cl_2 and (b) Fe**L9**Cl_2.

(a)

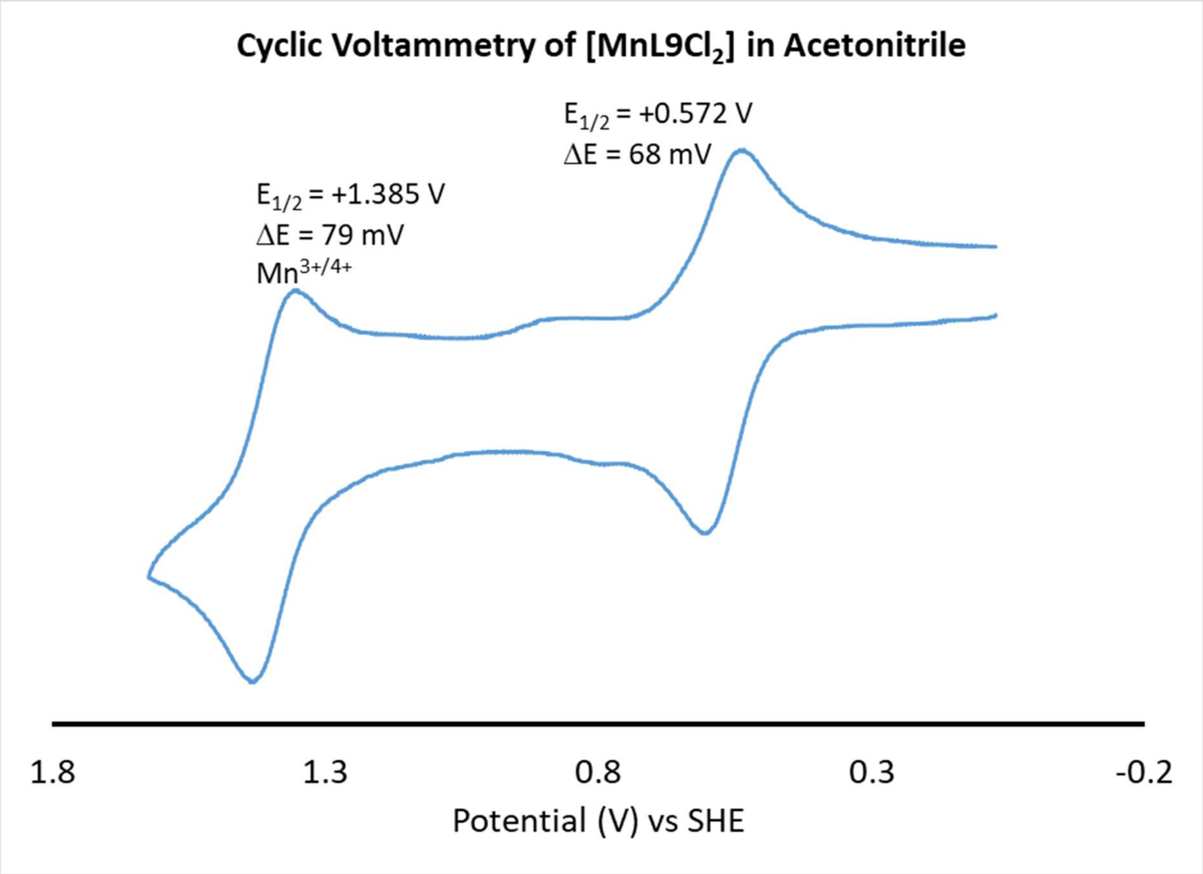

(b)

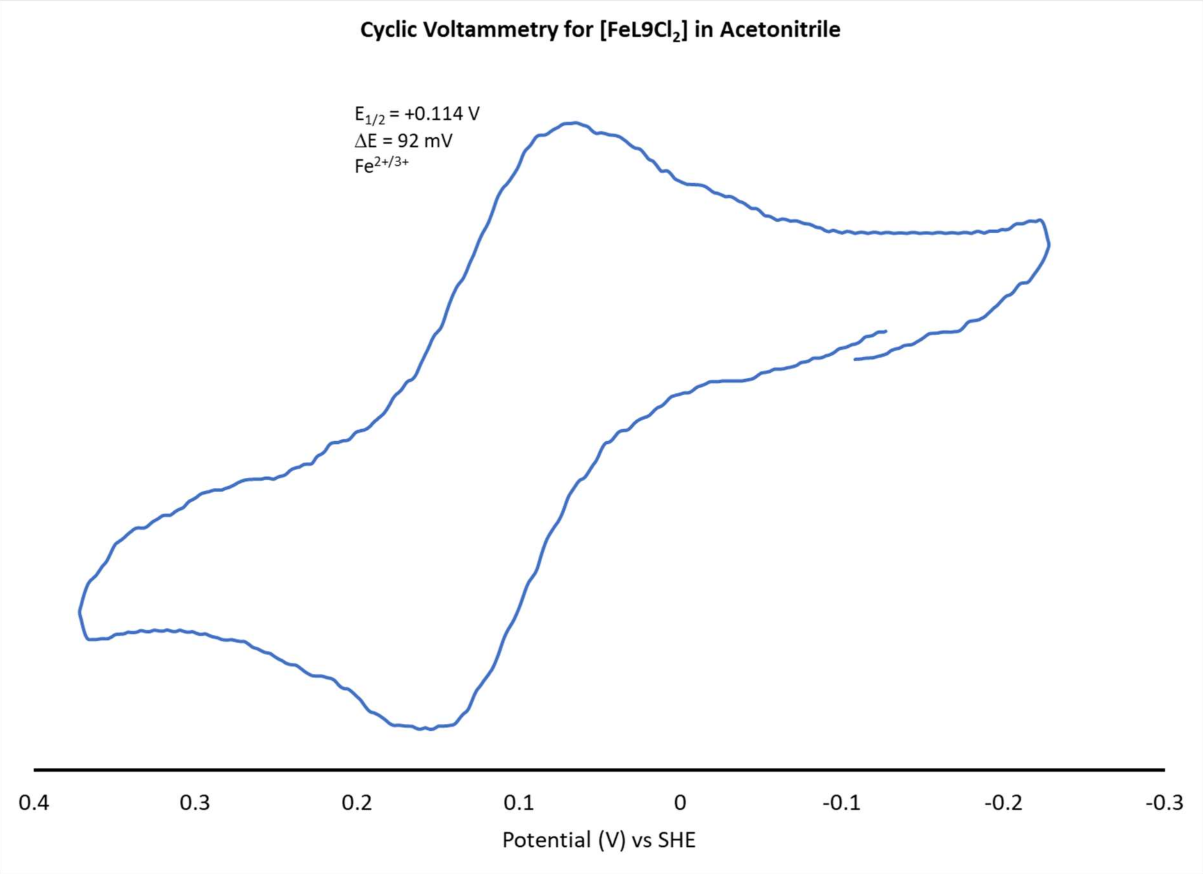

Figure S19. Cyclic Voltammograms in MeCN for (a) Mn**L10**Cl_2 and (b) Fe**L10**Cl_2.

(a)

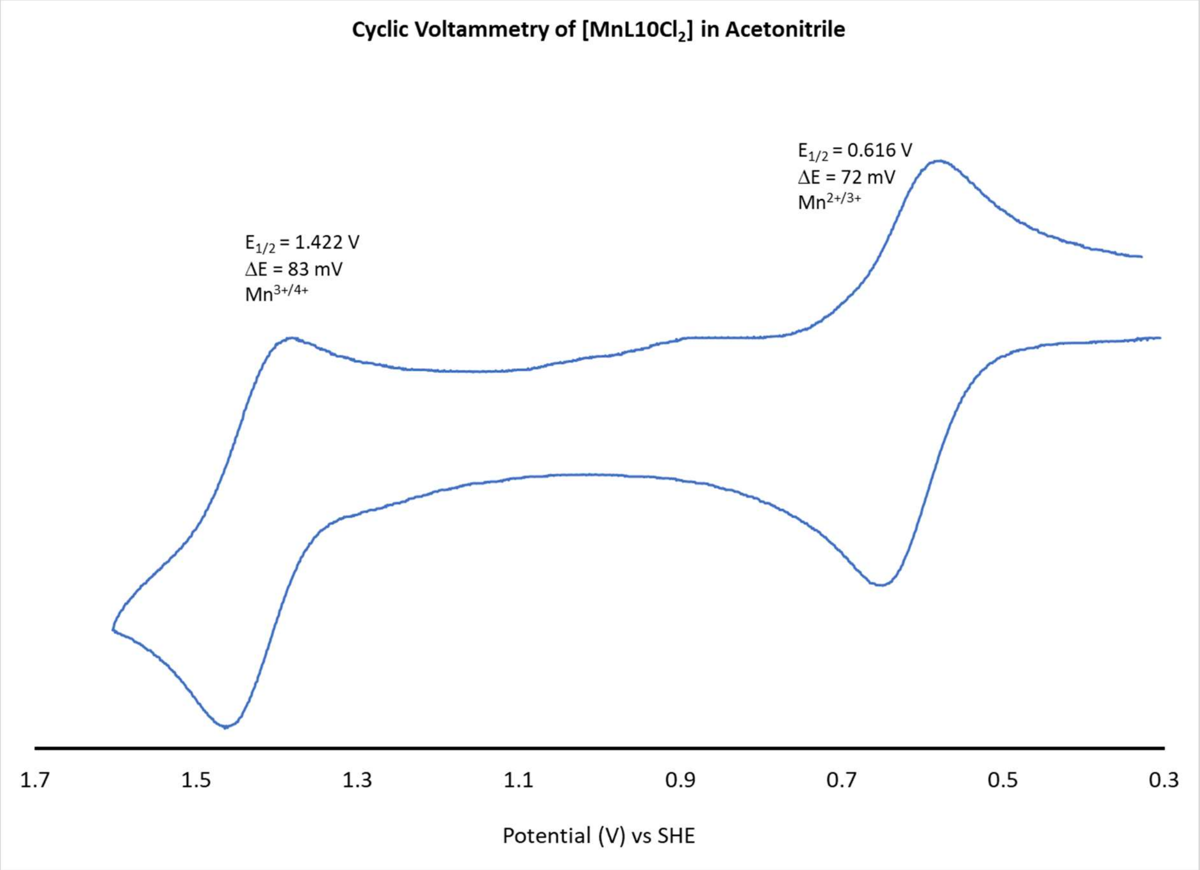

(b)

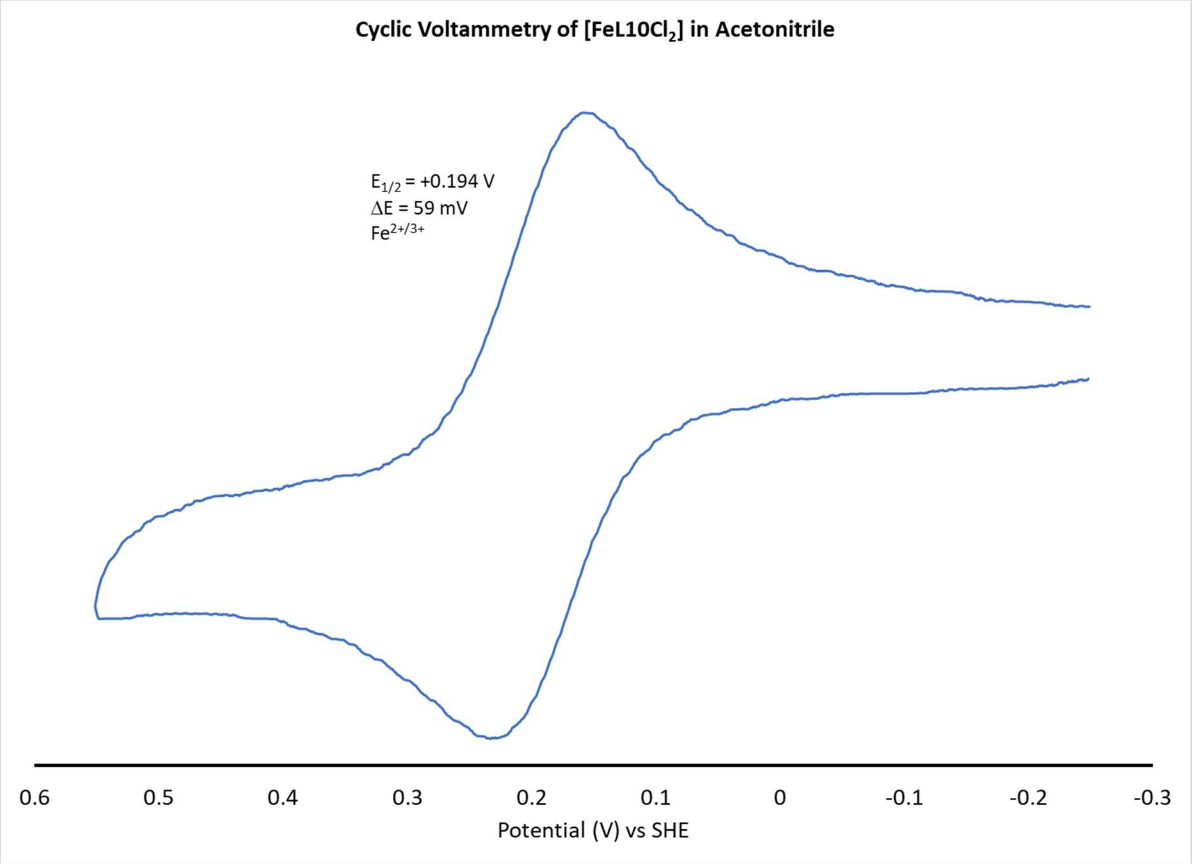

Figure S20. Cyclic Voltammograms in MeCN for (a) Mn**L11**Cl_2 and (b) Fe**L11**Cl_2.

(a)

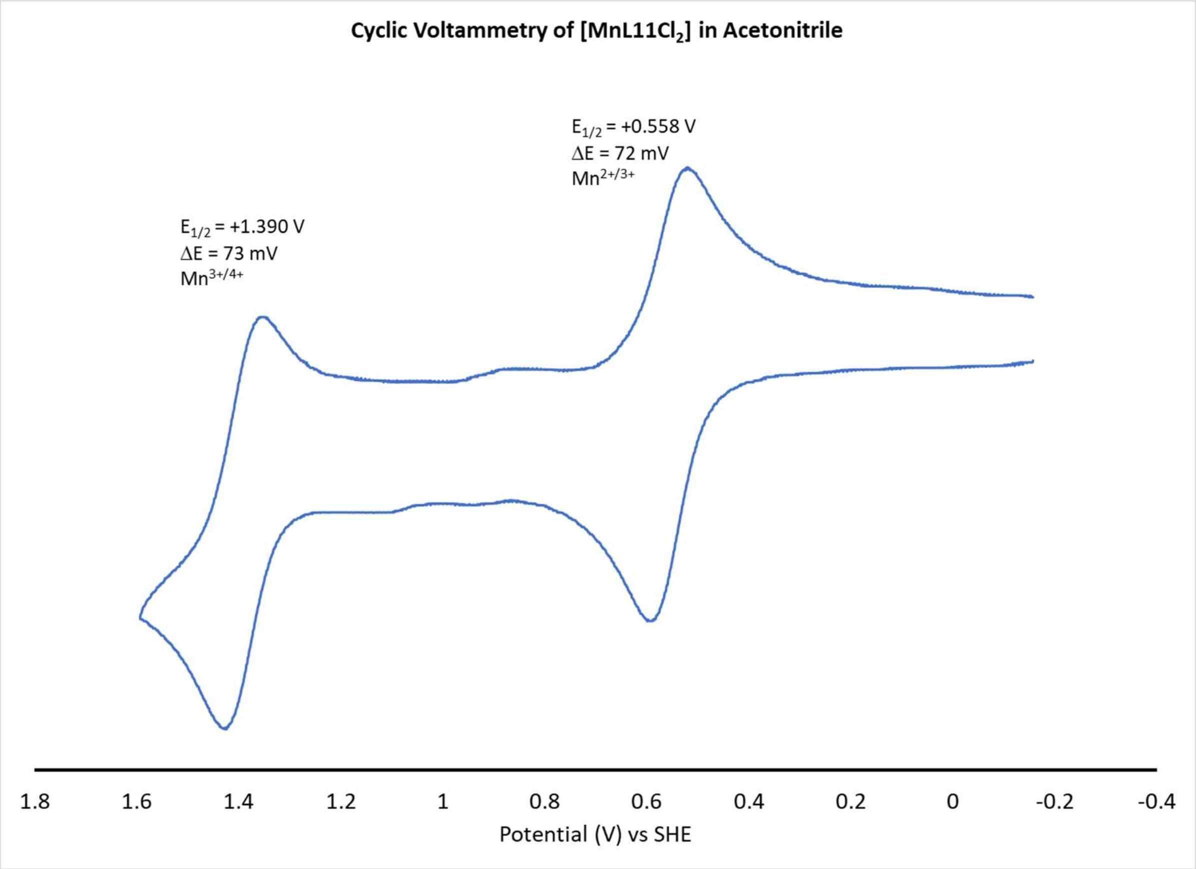

(b)

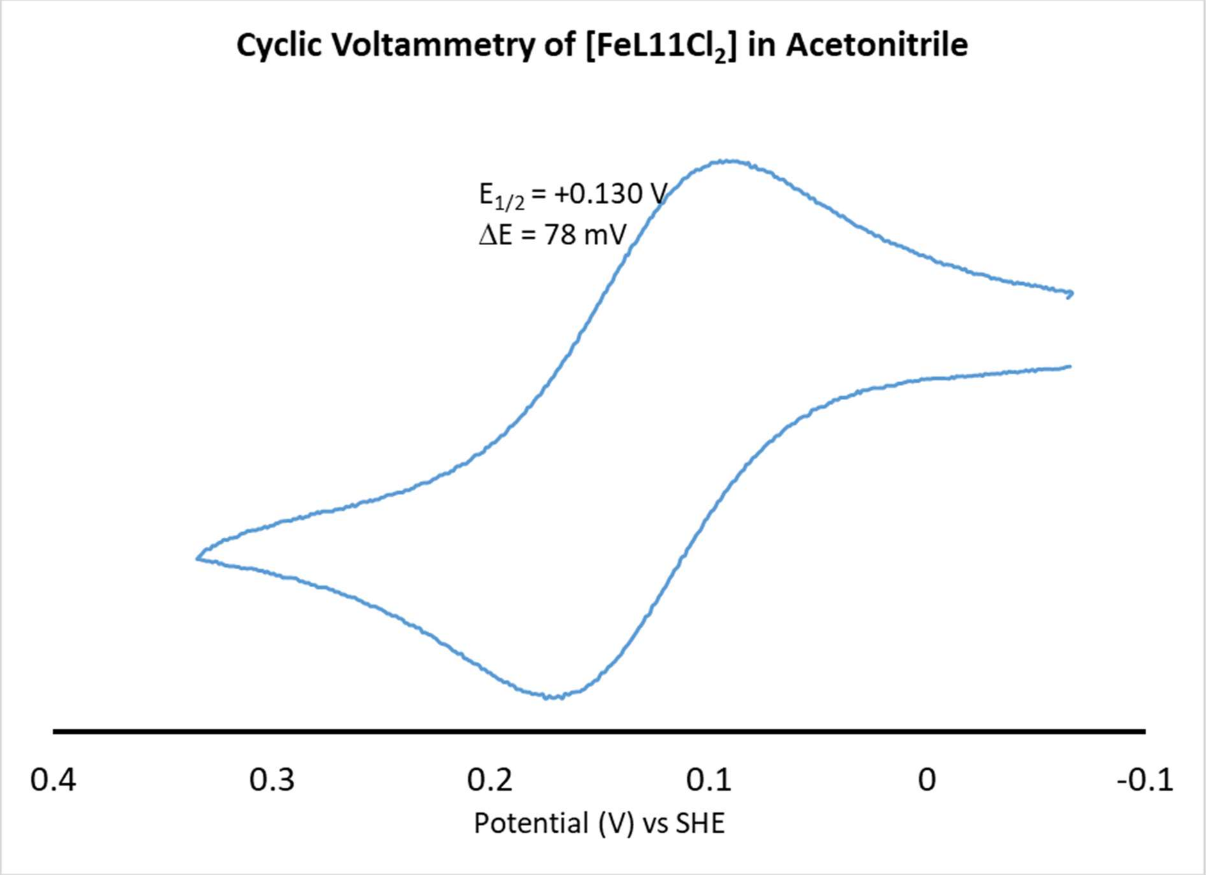

Figure S21. Cyclic Voltammograms in MeCN for (a) Mn**L12**Cl_2 and (b) Fe**L12**Cl_2.

(a)

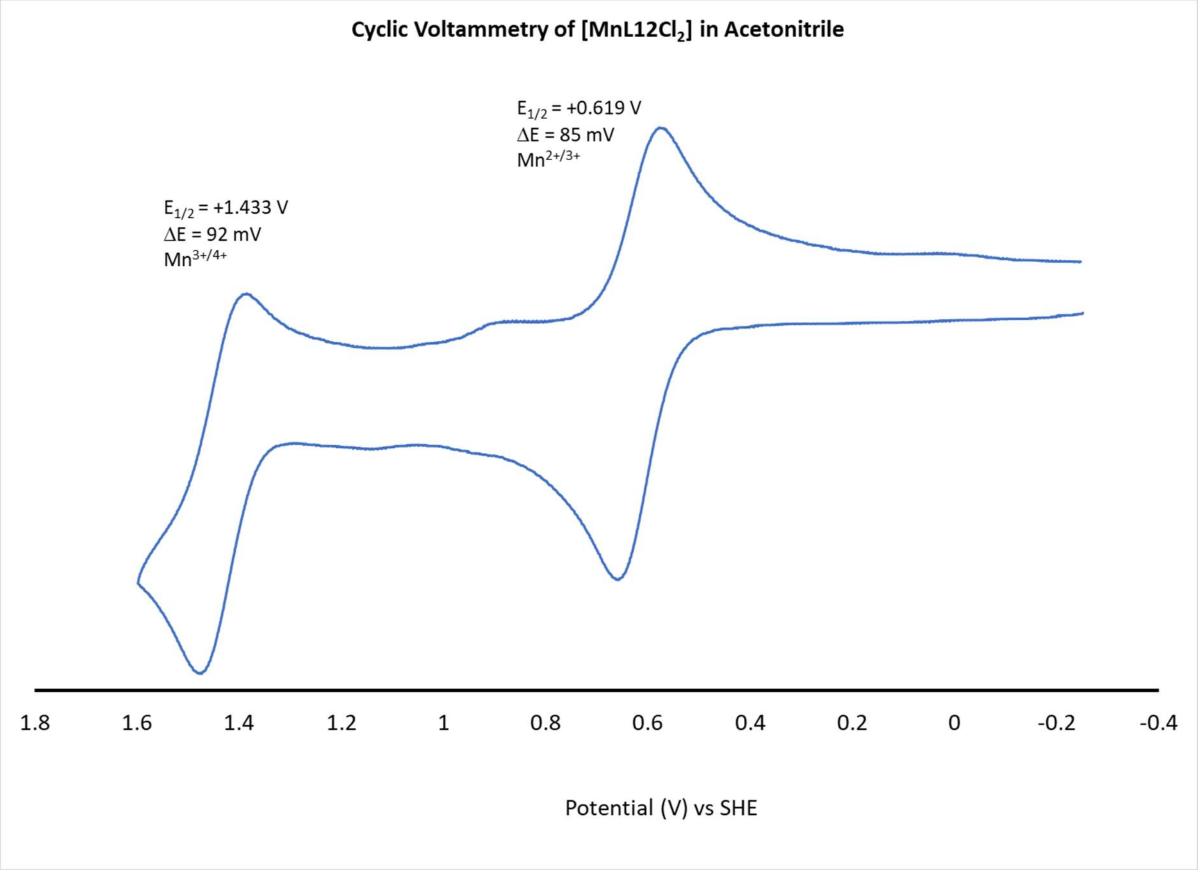

(b)

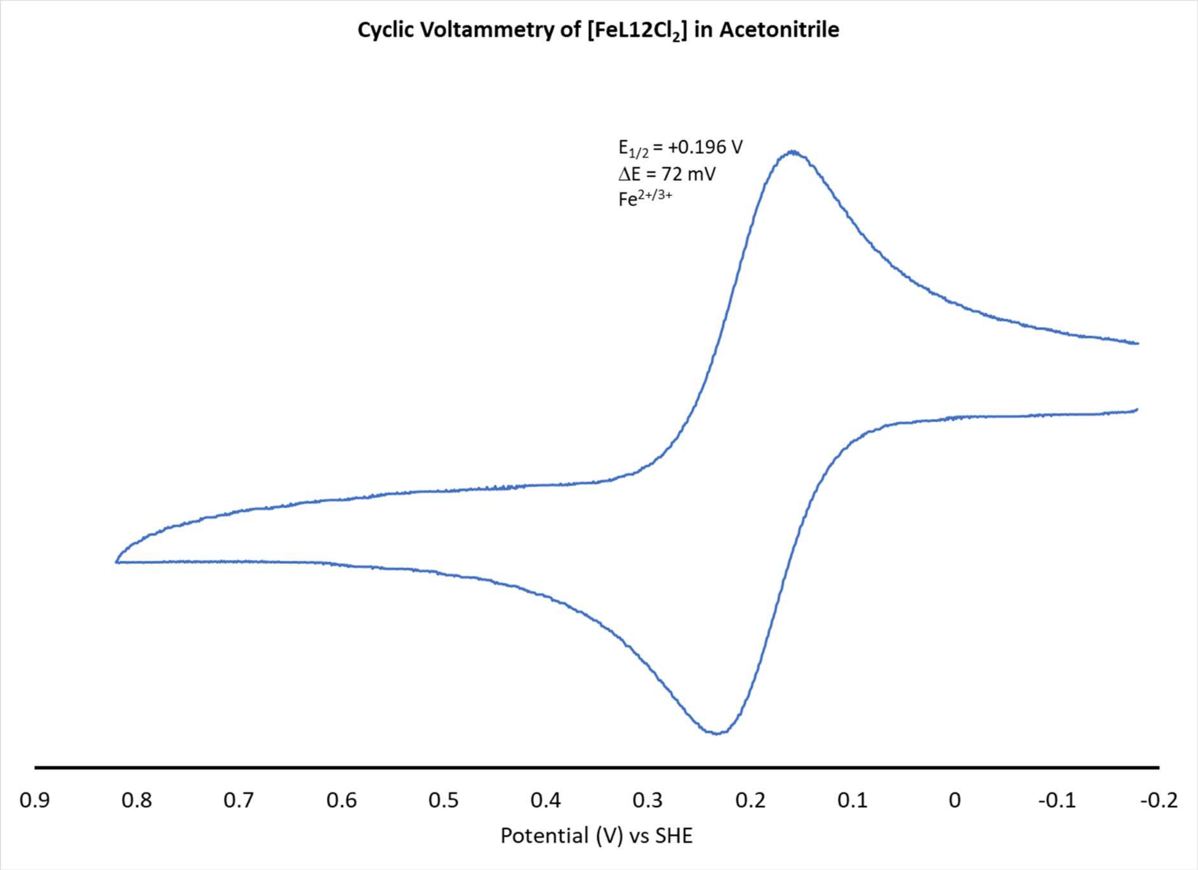

Figure S22. Dye bleaching plots (At/Ao vs time) of Methylene Blue (MB), Methyl Orange (MO), and Rhodamine B (RhB) for catalysts (a) Fe**L1**Cl_2 and (b) Mn**L1**Cl_2.

(a) 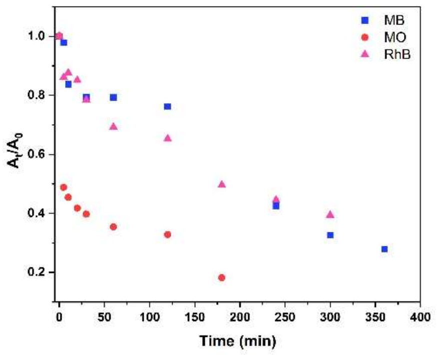

(b) 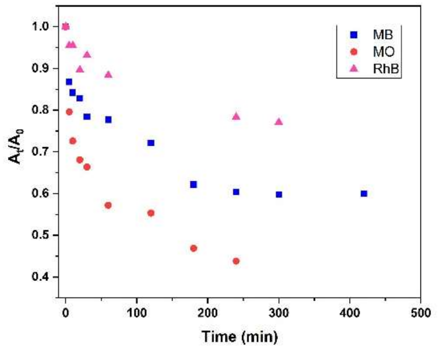

For [Fe**L1**Cl_2] complex, the dye bleaching was fastest for MO, bleaching of MB and RhB was comparable. The turnover frequency (hr $^{-1}$) (TOF) was less than 1 for MB and RhB, while TOF for MO was close to 1. For [Mn**L1**Cl_2] complex, the dye bleaching rate was fastest for MO and slowest for RhB. The TOF value was less than 0.5 for MB and RhB, while TOF value was close to 1 for MO. The TOF values of [Fe**L1**Cl_2] complex was higher than [Mn**L1**Cl_2] complex for MB and RhB, while the TOF of both of the complexes was comparable for MO.

Figure S23. Dye bleaching plots (At/Ao vs time) of Methylene Blue (MB), Methyl Orange (MO), and Rhodamine B (RhB) for catalysts (a) Fe**L2**Cl_2 and (b) Mn**L2**Cl_2.

(a) 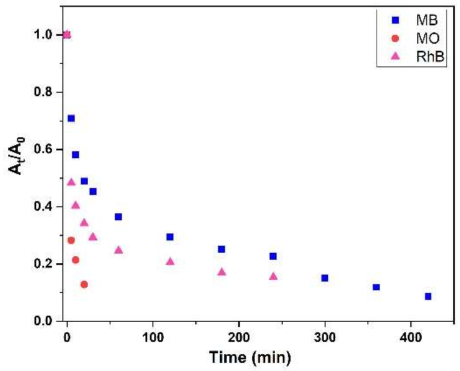

(b) 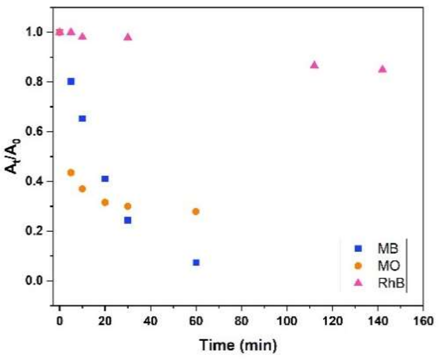

The degradation kinetics are fastest for MO and comparable for MB & RhB using [Fe**L2**Cl_2] catalyst. However, the degradation rate using [Mn**L2**Cl_2] catalyst is comparable for MO and MB, and slower for RhB than MO and MB. The TOF values of [Fe**L2**Cl_2] catalyst for bleaching of MB and RhB is ~1.15 and ~3.62 respectively. In our experience, degradation of complex structured dye RhB has been most challenging leading to a TOF value less than 0.5 for most of our catalysts. [Fe**L2**Cl_2] is our premium catalyst for degradation of RhB with a very high TOF exceeding 3. Nevertheless, the efficiency of [Fe**L2**Cl_2] for bleaching of MO was outstanding with a very high TOF value of 31.35. Thus, [Fe**L2**Cl_2] has turned out to be one of our best catalysts with an extraordinary high TOF value for all three dyes. [Mn**L2**Cl_2] exhibited a very poor performance in degradation of all three dyes with TOF values less than 0.5.

Figure S24. Dye bleaching plots (At/Ao vs time) of Methylene Blue (MB), Methyl Orange (MO), and Rhodamine B (RhB) for catalysts (a) Fe**L3**Cl_2 and (b) Mn**L3**Cl_2.

(a)

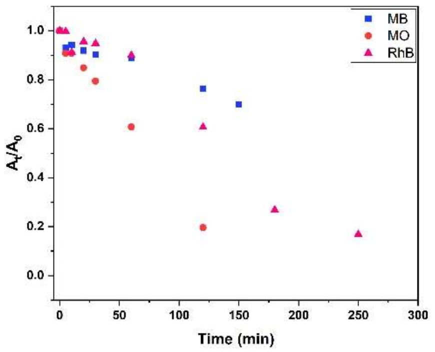

(b)

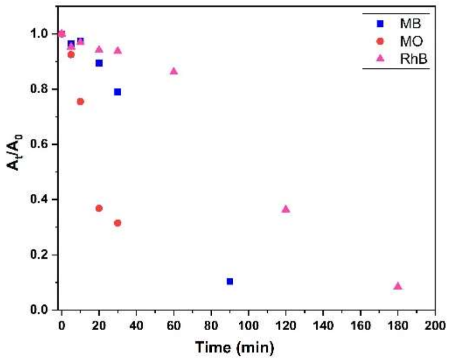

The [Fe**L3**Cl_2] complex, degrades MO fastest and degrades MB slowest. In the case of [Mn**L3**Cl_2], bleaching of MO is fastest and bleaching of RhB is slowest. The TOF values of [Fe**L3**Cl_2] are less than 1 for bleaching of MB and RhB, and the TOF value is between 1 and 1.5 for MO. [Mn**L3**Cl_2] has turned out to be a very efficient catalyst for bleaching of all three dyes. TOF values of [Mn**L3**Cl_2] is between 1.3 and 2 for bleaching of MB, between 1 and 1.5 for bleaching of RhB and TOF values for bleaching of MO is between 6.7 and 7.2. Undoubtedly, [Mn**L3**Cl_2] works as a better catalyst than the [Fe**L3**Cl_2] analogue.

Figure S25. Dye bleaching plots (At/Ao vs time) of Methylene Blue (MB), Methyl Orange (MO), and Rhodamine B (RhB) for catalysts (a) Fe**L4**Cl_2 and (b) Mn**L4**Cl_2.

(a)

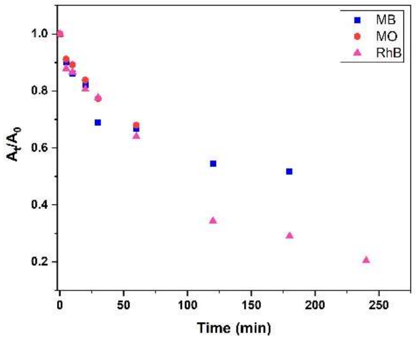

(b)

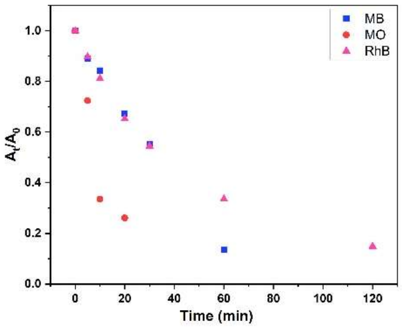

The [Fe**L4**Cl_2] complex decomposition rate is nearly equivalent for all three dyes in the beginning, but with increasing time MO degrades completely, degradation of RhB slows down and decomposition of MB saturates. In the case of [Mn**L4**Cl_2], the degradation of MO is fastest, and the decomposition of MB and RhB is comparable until 30 minutes, after which MB decomposes faster than RhB. The TOF value of [Fe**L4**Cl_2] for bleaching of MB is less than 0.5 and between 0.5 and 1 for bleaching of RhB; TOF value for bleaching of MO is slightly higher with the value is ~1.2. Although [Fe**L4**Cl_2] was not very efficient in bleaching of dyes, [Mn**L4**Cl_2] was a competent oxidation catalyst in bleaching of all three dyes. In our experience with the three dyes, bleaching of MO has been the easiest, and bleaching of MB and RhB has been difficult. With most of our catalysts, TOF values for bleaching of MB and RhB were less than 0.5. In the case of [Mn**L4**Cl_2], the average TOF values for bleaching of MB and RhB were calculated to be 2.03 and 1.05 respectively. On the other hand, the efficiency of [Mn**L4**Cl_2] in bleaching of MO has been excellent with an average TOF value of 16.26. Therefore, [Mn**L4**Cl_2] has been an efficient oxidation catalyst in bleaching of all three dyes, whereas, [Fe**L4**Cl_2] has shown poor performance in doing the same.

Figure S26. Dye bleaching plots (At/Ao vs time) of Methylene Blue (MB), Methyl Orange (MO), and Rhodamine B (RhB) for catalysts (a) Fe**L5**Cl_2 and (b) Mn**L5**Cl_2.

(a)

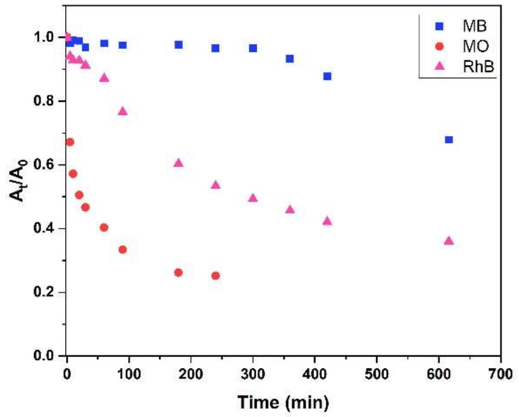

(b)

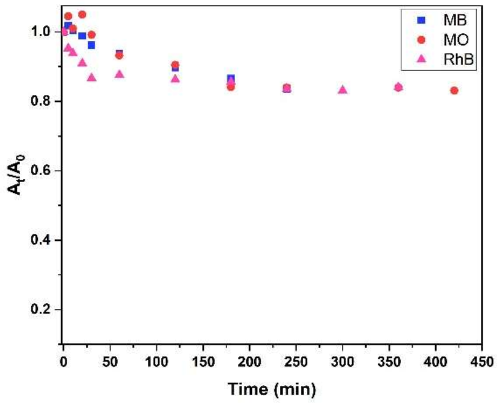

The decomposition rate is fastest for MO and slowest for MB using the [Fe**L5**Cl_2] catalysts. The decomposition of dyes is very slow and comparable for all three dyes using [Mn**L5**Cl_2] catalyst. The TOF values of [Fe**L5**Cl_2] was very low (~0.1 for MB and ~0.2 for RhB) for MB and RhB, however, the TOF value for bleaching of MO was appreciable (4.08) using the[Fe**L5**Cl_2] catalyst. Degradation of MO is relatively easy owing to its simpler structure compared to the other two dyes. As expected from the figure (b), [Mn**L5**Cl_2] exhibited very poor performance in bleaching of all three dyes, the TOF values are ~ 0.1 for MB and RhB, and ~0.6 for MO. Thus, neither [Fe**L5**Cl_2] nor [Mn**L5**Cl_2] turned out to be an efficient catalyst.

Figure S27. Dye bleaching plots (At/Ao vs time) of Methylene Blue (MB), Methyl Orange (MO), and Rhodamine B (RhB) for catalysts (a) Fe**L6**Cl_2 and (b) Mn**L6**Cl_2.

(a)

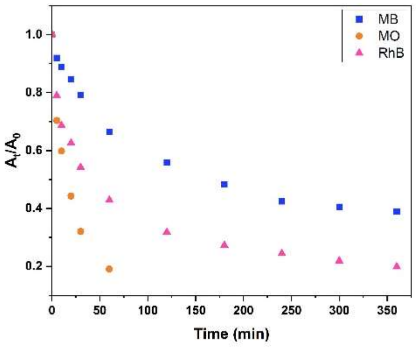

(b)

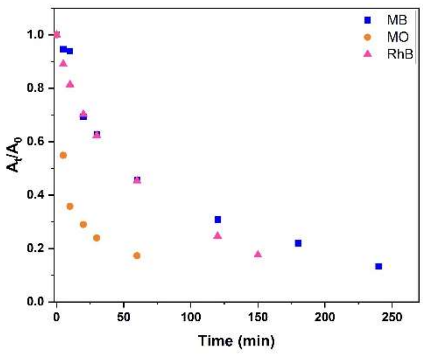

For the [Fe**L6**Cl_2] catalyst, decomposition of MO is fastest and MB degrades at the slowest rate. In the case of [Mn**L6**Cl_2] catalyst, bleaching of MO was fastest and decomposition rates of MB and RhB were comparable. The average TOF values for bleaching of Mb and RhB were 0.45 and 0.62 using [Fe**L6**Cl_2] catalyst, whereas, the average TOF value is 3.41 for bleaching of MO using [Fe**L6**Cl_2]. The TOF values suggest that [Fe**L6**Cl_2] is medium catalyst for bleaching of dyes. Nevertheless, [Mn**L6**Cl_2] turned out to be an excellent catalyst for bleaching of all three dyes. The average TOF values using [Mn**L6**Cl_2] catalyst was 0.74 and 1.27 for bleaching of MB and RhB respectively. Since most of our catalysts have exhibited TOF values less than 0.5 for bleaching of MB and RhB, [Mn**L6**Cl_2] can be termed an good catalyst for degradation of those two dyes. On the other hand, degradation of MO is relatively easy compared to MB and RhB. [Mn**L6**Cl_2] has exhibited superiority in bleaching of MO with a significantly high average TOF value of 16.14.

Figure S28. Dye bleaching plots (At/Ao vs time) of Methylene Blue (MB), Methyl Orange (MO), and Rhodamine B (RhB) for catalysts (a) Fe**L7**Cl_2 and (b) Mn**L7**Cl_2.

(a) (b)

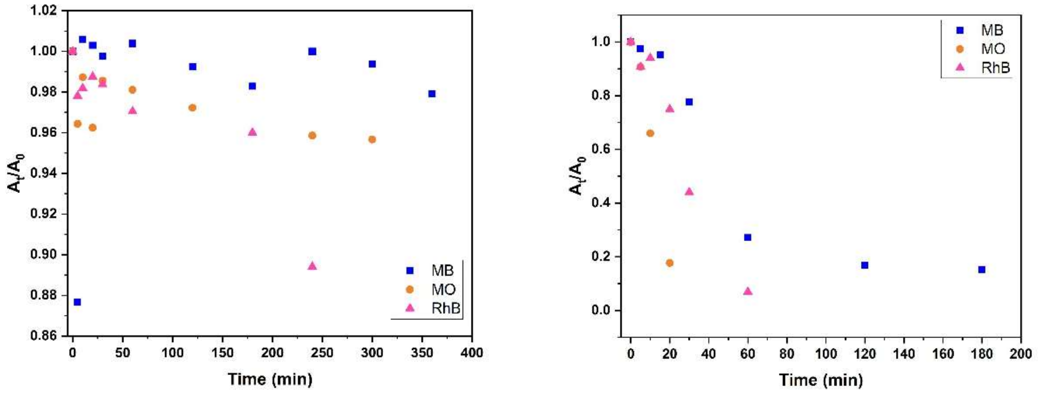

The decomposition rate of all three dyes are slow and comparable for the [Fe**L7**Cl_2] catalyst. Similarly, the degradation rate is fast yet analogous for all three dyes using the [Mn**L7**Cl_2] catalyst. The TOF values of [Fe**L7**Cl_2] catalyst were ~0.1 only for all three dyes. [Mn**L7**Cl_2] has proved to be an outstanding catalyst for bleaching of dyes. The average TOF value using [Mn**L7**Cl_2] for degradation of complex structure MB was 2.64, which is significantly higher than most of our catalysts. Rhodamine B has been most resistant to bleaching so far and it was very hard to achieve TOF value of 1 for RhB decomposition. Surprisingly, [Mn**L7**Cl_2] was able to degrade RhB with an average TOF value of 2.34, which is exceptionally high as compared to our other catalysts. Therefore, [Mn**L7**Cl_2] is an extra-ordinary catalyst for decomposition of both MB and RhB within this test group. The average TOF value for bleaching of MO using [Mn**L7**Cl_2] is 8.32, which is appreciable, but not the highest.

Figure S29. Dye bleaching plots (At/Ao vs time) of Methylene Blue (MB), Methyl Orange (MO), and Rhodamine B (RhB) for catalysts (a) Fe**L8**Cl_2 and (b) Mn**L8**Cl_2.

(a) (b)

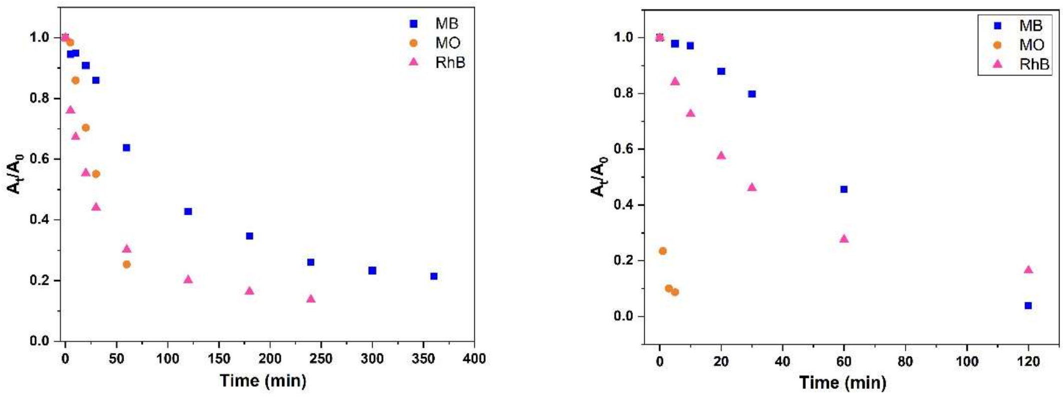

The decomposition rate of MO and RhB is comparable, but the rate of decomposition of RhB is slower for the [Fe**L8**Cl_2] catalyst. In the case of [Mn**L8**Cl_2], the decomposition of MO is very fast and the decomposition of MB and RhB are comparable. The average TOF value for bleaching of MB and MO using [Fe**L8**Cl_2] are 0.57 and 2.82 respectively, which suggests [Fe**L8**Cl_2] is an average catalysts for degradation of MB and MO among this screening set. However, the average TOF value for bleaching of RhB using [Fe**L8**Cl_2] is 1.54, which is significantly higher than most other catalysts. [Mn**L8**Cl_2] is an exceptional catalyst for bleaching all three dyes. The average TOF value for bleaching of MB using [Mn**L8**Cl_2] catalyst is 2.09, which is high for this test group. The performance of [Mn**L8**Cl_2] in degrading RhB was also quite appreciable with an average TOF value of 1.46. [Mn**L8**Cl_2] is the most efficient catalyst of the set to degrade MO dye with an exceptionally high average TOF value of 108.9.

Figure S30. Dye bleaching plots (At/Ao vs time) of Methylene Blue (MB), Methyl Orange (MO), and Rhodamine B (RhB) for catalysts (a) Fe**L9**Cl_2 and (b) Mn**L9**Cl_2.

(a) (b)

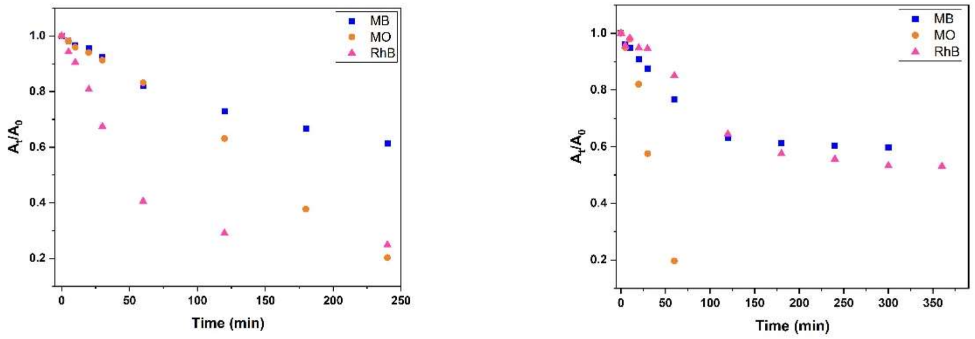

Unlike most of the dyes, the degradation rate of RhB is fastest for the [Fe**L9**Cl_2] catalyst. The decomposition rate of MO and MB using the [Fe**L9**Cl_2] catalyst are comparable through the first 100 minutes, after which bleaching of MB saturates but MO continues degrading. Degradation of MO is fastest and decomposition rate of MB and RhB is comparable using the [Mn**L9**Cl_2] catalyst. The average TOF values of the [Fe**L9**Cl_2] catalyst are 0.35, 0.77 and 0.92 for bleaching of MB, MO and RhB respectively. The TOF values suggest that [Fe**L9**Cl_2] is an average catalyst for MB degradation. However, the catalytic efficiency of [Fe**L9**Cl_2] is appreciable for degradation of the most complex structured dye RhB. On the contrary, [Fe**L9**Cl_2] is not efficient in bleaching of the simplest structured dye MO, which is a notable result against the general trend. The average TOF values using [Mn**L9**Cl_2] catalyst is 0.53, 3.13 and 0.35 for bleaching of MB, MO and RhB respectively. The TOF values conclude [Mn**L9**Cl_2] to be an average catalyst for this set of screened catalysts.

Figure S31. Dye bleaching plots (At/Ao vs time) of Methylene Blue (MB), Methyl Orange (MO), and Rhodamine B (RhB) for catalysts (a) Fe**L10**Cl_2 and (b) Mn**L10**Cl_2.

(a) (b)

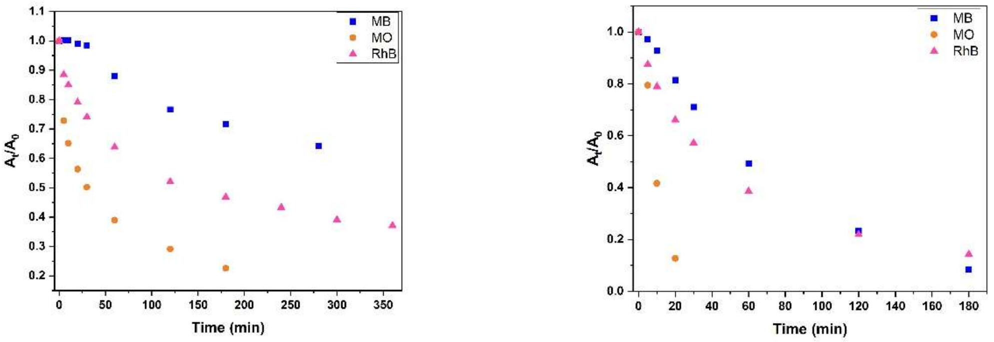

The decomposition rate of MO is fastest and RhB is slowest using the [FeL**10**Cl_2] catalyst. For [Mn**L10**Cl_2], the decomposition rate of MO is fastest, and the degradation rate of MB and RhB are comparable. The average TOF value using [FeL**10**Cl_2] catalyst is 0.171, 2.2, and 0.35 for bleaching of MB, MO and RhB, below average for this screening set. The average TOF values using [MnL**10**Cl_2] catalyst are 1.23 and 1.16 for bleaching of complex structured dyes MB and RhB respectively, which are for this screening set. The average TOF value of the [MnL**10**Cl_2] catalyst for bleaching of MO is 9.40, which suggests that the catalytic efficiency of [MnL**10**Cl_2] is quite high for bleaching of MO.

Figure S32. Dye bleaching plots (At/Ao vs time) of Methylene Blue (MB), Methyl Orange (MO), and Rhodamine B (RhB) for catalysts (a) Fe**L11**Cl_2 and (b) Mn**L11**Cl_2.

(a) (b)

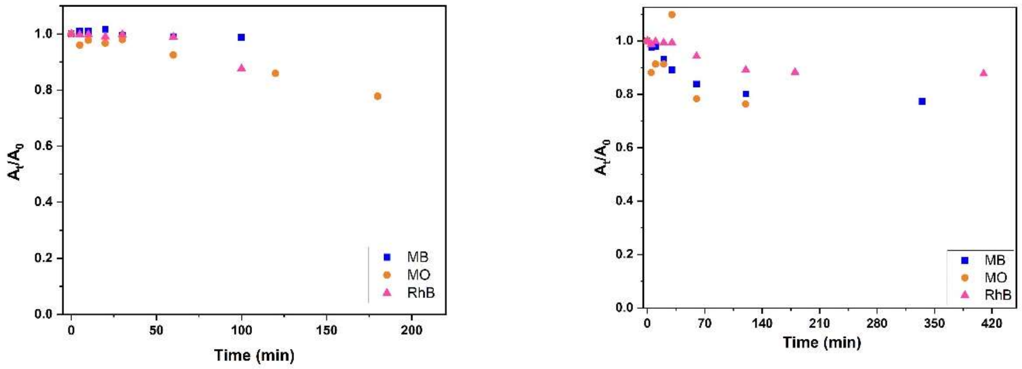

The decomposition rates of dyes using both [FeL**11**Cl_2] and [MnL**11**Cl_2] catalysts are very slow and comparable for all three dyes. The average TOF values using the [FeL**11**Cl_2] catalyst were 0.19, 0.43, and 0.26 for bleaching of MB, MO and RhB respectively. The average TOF values for [Mn**L11**Cl_2] were 0.18, 0.86, and 0.15 for degradation of MB, MO and RhB respectively. The low TOF values determine that both [FeL**11**Cl_2] and [MnL**11**Cl_2] are poor catalyst for bleaching all three dyes, compared to this screening set.

Figure S33. Dye bleaching plots (At/Ao vs time) of Methylene Blue (MB), Methyl Orange (MO), and Rhodamine B (RhB) for catalysts (a) Fe**L12**Cl_2 and (b) Mn**L12**Cl_2.

(a) (b)

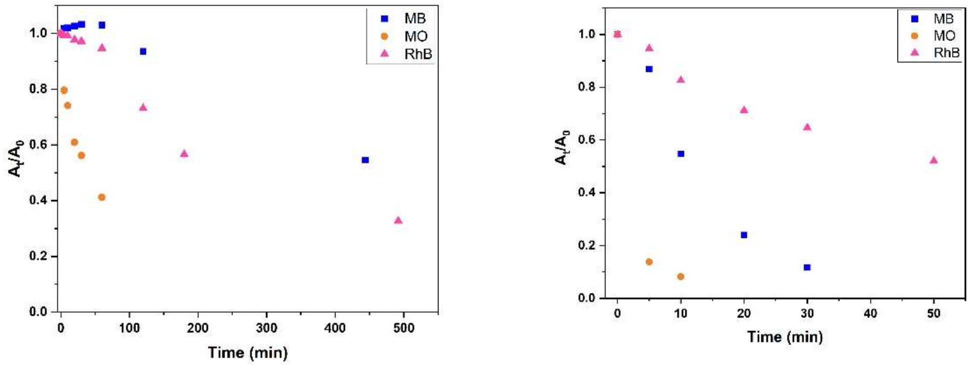

The [FeL**12**Cl_2] catalyst degrades MO fastest and degrades MB slowest. [MnL**12**Cl_2] bleaches MO very fast, with bleaching of MB and RhB more slowly. The average TOF values using the [Fe**L12**Cl_2] catalysts were 0.25, 1.26, and 0.70 for bleaching of MB, MO and RhB respectively. The TOF values recommend that [FeL**12**Cl_2] is an average catalyst for bleaching of dyes. However, the catalytic efficiency of [MnL**12**Cl_2] was outstanding for bleaching of all three dyes with an average TOF values 5.87, 36.49, 1.61 for MB, MO, and RhB respectively. [Mn**L12**Cl_2] is best catalyst among this screening set for bleaching MB. Remarkably, this catalyst came up with an extraordinarily high TOF value of 5.87, whereas most of our catalysts struggled to reach the TOF value of 1 for bleaching of complex structured dye MB. [MnL**12**Cl_2] is our second best catalyst for bleaching MO with a significantly high TOF value of 36.49. The catalytic efficiency of [MnL**12**Cl_2] is also quite impressive for bleaching of the RhB, with a TOF value of 1.61.

[Mn(L1)Cl_2]

Table S1 Crystal data and structure refinement for [Mn(L1)Cl2].

Identification code	[Mn(L1)Cl2]
Empirical formula	$C_{10}H_{22}Cl_2MnN_4$
Formula weight	324.15
Temperature/K	120.02
Crystal system	monoclinic
Space group	Cc
a/Å	12.3081(13)
b/Å	10.1119(13)
c/Å	11.2029(12)
α/°	90
β/°	96.578(2)
γ/°	90
Volume/Å^3	1385.1(3)
Z	4
ρ_{calc}g/cm^3	1.554
μ/mm^{-1}	1.324
F(000)	676.0
Crystal size/mm^3	0.355 × 0.247 × 0.142
Radiation	MoKα (λ = 0.71073)
2Θ range for data collection/°	5.228 to 56.576
Index ranges	-16 ≤ h ≤ 14, -13 ≤ k ≤ 13, -14 ≤ l ≤ 14
Reflections collected	13204
Independent reflections	3268 [R_{int} = 0.0178, R_{sigma} = 0.0211]
Data/restraints/parameters	3268/2/167
Goodness-of-fit on F^2	1.042
Final R indexes [I>=2σ (I)]	R_1 = 0.0131, wR_2 = 0.0319
Final R indexes [all data]	R_1 = 0.0133, wR_2 = 0.0320
Largest diff. peak/hole / e Å^{-3}	0.18/-0.12
Flack parameter	0.077(10)

Table S2 Fractional Atomic Coordinates (×10^4) and Equivalent Isotropic Displacement Parameters (Å^2×10^3) for [Mn(L1)Cl2]. U_{eq} is defined as 1/3 of of the trace of the orthogonalised U_{IJ} tensor.

Atom	*x*	*y*	*z*	U(eq)
Mn1	4641.7(2)	3794.9(2)	4687.1(2)	10.56(5)
Cl2	3087.6(3)	4696.7(4)	3348.5(3)	16.71(8)
Cl1	5395.1(3)	5895.3(4)	5408.4(3)	17.78(8)
N2	4099.6(11)	1608.0(13)	4368.5(11)	13.0(2)
N1	3906.1(11)	3143.5(14)	6365.9(12)	14.3(3)
N3	5718.5(11)	3079.8(13)	3275.6(12)	14.1(3)
N4	6027.1(11)	2485.4(13)	5685.9(11)	14.1(3)
C3	4139.1(14)	1508.0(15)	3068.6(13)	15.5(3)
C6	6999.8(13)	3029.1(17)	5217.8(14)	17.8(3)
C4	5280.7(14)	1805.9(15)	2743.7(13)	16.2(3)

Table S2 Fractional Atomic Coordinates (×10[4]) and Equivalent Isotropic Displacement Parameters (Å[2]×10[3]) for [Mn(L1)Cl2]. U_{eq} is defined as 1/3 of of the trace of the orthogonalised U_{IJ} tensor.

Atom	*x*	*y*	*z*	U(eq)
C2	2986.1(13)	1683.1(16)	4719.9(14)	15.8(3)
C1	2990.4(14)	2219.1(16)	6010.5(14)	16.9(3)
C7	5929.1(14)	2845.8(16)	6941.1(14)	16.9(3)
C10	4763.9(14)	592.3(15)	5056.3(13)	15.8(3)
C8	4777.0(14)	2597.0(16)	7264.5(13)	17.0(3)
C9	5938.7(15)	1044.4(15)	5473.0(15)	16.9(3)
C5	6876.9(14)	3003.8(16)	3830.1(14)	17.5(3)

Table S3 Anisotropic Displacement Parameters (Å[2]×10[3]) for [Mn(L1)Cl2]. The Anisotropic displacement factor exponent takes the form: $-2\pi^2[h^2a^{*2}U_{11}+2hka^*b^*U_{12}+\ldots]$.

Atom	U_{11}	U_{22}	U_{33}	U_{23}	U_{13}	U_{12}
Mn1	12.57(11)	9.38(9)	9.53(10)	-0.45(9)	0.44(8)	-0.73(9)
Cl2	15.77(18)	14.95(17)	18.67(17)	3.03(13)	-1.30(14)	1.33(14)
Cl1	22.4(2)	14.16(17)	16.48(18)	-3.30(14)	0.89(15)	-5.59(15)
N2	16.5(7)	12.0(6)	10.1(6)	0.4(5)	0.2(5)	-0.4(5)
N1	16.4(7)	13.9(6)	12.5(6)	-1.9(5)	1.1(5)	-1.8(5)
N3	18.6(7)	11.8(6)	11.8(6)	1.1(5)	1.9(5)	-0.1(5)
N4	16.1(7)	14.5(6)	11.6(6)	-0.2(5)	0.9(5)	0.0(5)
C3	23.9(8)	12.4(7)	9.8(7)	-2.5(5)	0.0(6)	-2.0(6)
C6	14.0(8)	20.8(8)	18.4(8)	1.1(6)	0.4(6)	0.4(6)
C4	24.8(9)	12.6(7)	11.5(7)	-2.2(5)	3.6(6)	0.4(6)
C2	17.9(8)	15.8(7)	13.7(7)	-1.3(6)	1.6(6)	-5.8(6)
C1	17.2(8)	19.3(8)	14.5(7)	-1.9(6)	3.9(6)	-5.0(6)
C7	19.8(8)	20.1(7)	9.9(7)	0.4(6)	-2.6(6)	0.1(6)
C10	23.9(9)	10.3(7)	12.9(7)	1.7(5)	1.3(6)	0.9(6)
C8	24.0(9)	17.8(7)	8.8(7)	0.5(6)	0.7(6)	-2.0(6)
C9	23.2(9)	12.8(7)	14.5(7)	2.9(5)	1.0(7)	4.0(6)
C5	16.2(8)	18.7(8)	18.4(8)	-0.1(6)	6.0(6)	0.8(6)

Table S4 Bond Lengths for [Mn(L1)Cl2].

Atom	Atom	Length/Å	Atom	Atom	Length/Å
Mn1	Cl2	2.4662(5)	N3	C4	1.4937(19)
Mn1	Cl1	2.4191(5)	N3	C5	1.491(2)
Mn1	N2	2.3255(14)	N4	C6	1.468(2)
Mn1	N1	2.2761(14)	N4	C7	1.471(2)
Mn1	N3	2.2935(13)	N4	C9	1.479(2)
Mn1	N4	2.3401(13)	C3	C4	1.521(2)
N2	C3	1.4661(19)	C6	C5	1.545(2)
N2	C2	1.471(2)	C2	C1	1.544(2)
N2	C10	1.4741(19)	C7	C8	1.524(2)
N1	C1	1.483(2)	C10	C9	1.537(2)
N1	C8	1.490(2)			

TableS 5 Bond Angles for [Mn(L1)Cl2].

Atom	Atom	Atom	Angle/˚	Atom	Atom	Atom	Angle/˚
Cl1	Mn1	Cl2	96.856(16)	C1	N1	C8	114.41(13)
N2	Mn1	Cl2	93.98(3)	C8	N1	Mn1	110.01(10)
N2	Mn1	Cl1	168.07(3)	C4	N3	Mn1	109.65(10)
N2	Mn1	N4	73.49(5)	C5	N3	Mn1	109.25(9)
N1	Mn1	Cl2	104.79(4)	C5	N3	C4	113.95(12)
N1	Mn1	Cl1	98.72(4)	C6	N4	Mn1	101.35(9)
N1	Mn1	N2	73.58(5)	C6	N4	C7	114.10(12)
N1	Mn1	N3	142.60(5)	C6	N4	C9	111.07(13)
N1	Mn1	N4	77.18(5)	C7	N4	Mn1	100.48(9)
N3	Mn1	Cl2	99.59(4)	C7	N4	C9	112.70(12)
N3	Mn1	Cl1	106.11(4)	C9	N4	Mn1	116.38(10)
N3	Mn1	N2	76.86(5)	N2	C3	C4	111.13(13)
N3	Mn1	N4	72.80(5)	N4	C6	C5	111.32(13)
N4	Mn1	Cl2	166.39(3)	N3	C4	C3	112.10(12)
N4	Mn1	Cl1	96.12(3)	N2	C2	C1	111.45(13)
C3	N2	Mn1	100.17(9)	N1	C1	C2	113.18(13)
C3	N2	C2	114.04(13)	N4	C7	C8	111.51(12)
C3	N2	C10	113.06(12)	N2	C10	C9	113.43(13)
C2	N2	Mn1	99.76(9)	N1	C8	C7	113.26(12)
C2	N2	C10	111.97(12)	N4	C9	C10	113.04(13)
C10	N2	Mn1	116.75(10)	N3	C5	C6	113.41(13)
C1	N1	Mn1	108.82(9)				

Table S6 Torsion Angles for [Mn(L1)Cl2].

A	B	C	D	Angle/˚	A	B	C	D	Angle/˚
Mn1	N2	C3	C4	59.07(12)	C3	N2	C10	C9	91.99(16)
Mn1	N2	C2	C1	-56.24(13)	C6	N4	C7	C8	163.96(13)
Mn1	N2	C10	C9	-23.44(16)	C6	N4	C9	C10	-138.14(13)
Mn1	N1	C1	C2	15.03(16)	C4	N3	C5	C6	-106.89(15)
Mn1	N1	C8	C7	10.69(15)	C2	N2	C3	C4	164.66(13)
Mn1	N3	C4	C3	12.60(14)	C2	N2	C10	C9	-137.53(14)
Mn1	N3	C5	C6	16.13(15)	C1	N1	C8	C7	133.52(14)
Mn1	N4	C6	C5	-54.89(13)	C7	N4	C6	C5	-161.92(13)
Mn1	N4	C7	C8	56.39(13)	C7	N4	C9	C10	92.41(16)
Mn1	N4	C9	C10	-22.90(16)	C10	N2	C3	C4	-65.91(16)
N2	C3	C4	N3	-51.34(16)	C10	N2	C2	C1	67.95(16)
N2	C2	C1	N1	29.81(19)	C8	N1	C1	C2	-108.45(16)
N2	C10	C9	N4	29.92(19)	C9	N4	C6	C5	69.37(16)
N4	C6	C5	N3	27.75(18)	C9	N4	C7	C8	-68.17(17)

Table S6 Torsion Angles for [Mn(L1)Cl2].

A	B	C	D	Angle/˚	A	B	C	D	Angle/˚
N4	C7	C8	N1	-48.40(17)	C5	N3	C4	C3	135.40(14)
C3	N2	C2	C1	-162.07(12)					

Table S7 Hydrogen Atom Coordinates (Å×10^4) and Isotropic Displacement Parameters (Å^2×10^3) for [Mn(L1)Cl2].

Atom	*x*	*y*	*z*	U(eq)
H1	3630(6)	3858(16)	6688(7)	17
H3	5682.6(14)	3660(15)	2709(14)	17
H3A	3620(8)	2130(9)	2656(6)	19
H3B	3926(4)	619(13)	2800(4)	19
H6A	7635(9)	2518(7)	5525(5)	21
H6B	7113(2)	3930(13)	5494(4)	21
H4A	5776(7)	1082(10)	3033(4)	19
H4B	5259.1(14)	1848.8(16)	1864(12)	19
H2A	2647(5)	778(12)	4667.7(16)	19
H2B	2528(6)	2278(8)	4145(8)	19
H1A	2289(10)	2681(7)	6075.5(17)	20
H1B	3039.2(15)	1464(11)	6579(9)	20
H7A	6112(3)	3784(14)	7064(2)	20
H7B	6453(8)	2326(7)	7475(8)	20
H10A	4791.8(14)	-209(11)	4550(7)	19
H10B	4402(5)	345(4)	5772(10)	19
H8A	4667(2)	1654(14)	7334.8(17)	20
H8B	4708.8(17)	2990(6)	8040(11)	20
H9A	6204(4)	578(7)	6218(11)	20
H9B	6412(7)	797(4)	4861(9)	20
H5A	7276(7)	3734(11)	3548(5)	21
H5B	7198(5)	2204(13)	3570(4)	21

Experimental

Single crystals of $C_{10}H_{22}Cl_2MnN_4$ **[[Mn(L1)Cl2]]** were **[]**. A suitable crystal was selected and **[]** on a **Bruker APEX-II** diffractometer. The crystal was kept at 120.02 K during data collection. Using Olex2 [1], the structure was solved with the SHELXT [2] structure solution program using Intrinsic Phasing and refined with the SHELXL [3] refinement package using Least Squares minimisation.

1. Dolomanov, O.V., Bourhis, L.J., Gildea, R.J, Howard, J.A.K. & Puschmann, H. (2009), J. Appl. Cryst. 42, 339-341.
2. Sheldrick, G.M. (2015). Acta Cryst. A71, 3-8.
3. Sheldrick, G.M. (2015). Acta Cryst. C71, 3-8.

Crystal structure determination of [[Mn(L1)Cl2]]

Crystal Data for $C_{10}H_{22}Cl_2MnN_4$ (M =324.15 g/mol): monoclinic, space group Cc (no. 9), a = 12.3081(13) Å, b = 10.1119(13) Å, c = 11.2029(12) Å, β = 96.578(2)°, V = 1385.1(3) Å^3, Z = 4, T = 120.02 K, μ(MoKα) = 1.324 mm^{-1}, $Dcalc$ = 1.554 g/cm^3, 13204 reflections measured (5.228° ≤ 2Θ ≤ 56.576°), 3268 unique (R_{int} = 0.0178, R_{sigma} = 0.0211) which were used in all calculations. The final R_1 was 0.0131 (I > 2σ(I)) and wR_2 was 0.0320 (all data).

Refinement model description

Number of restraints - 2, number of constraints - unknown.

Details:

```
1. Twinned data refinement
 Scales: 0.923(10)
 0.077(10)
2. Fixed Uiso
 At 1.2 times of:
  All C(H,H) groups, All N(H) groups
3.a Ternary CH refined with riding coordinates and stretchable bonds:
 N1(H1), N3(H3)
3.b Secondary CH2 refined with riding coordinates and stretchable bonds:
 C3(H3A,H3B), C6(H6A,H6B), C4(H4A,H4B), C2(H2A,H2B), C1(H1A,H1B), C7(H7A,H7B),
 C10(H10A,H10B), C8(H8A,H8B), C9(H9A,H9B), C5(H5A,H5B)
```

This report has been created with Olex2, compiled on 2020.11.12 svn.r5f609507 for OlexSys. Please let us know if there are any errors or if you would like to have additional features.

[Mn(L3)Cl_2]

Table S8 Crystal data and structure refinement for [Mn(L3)Cl2].

Identification code	[Mn(L3)Cl2]
Empirical formula	$C_{14}H_{28}Cl_2MnN_4$
Formula weight	378.24
Temperature/K	120.0
Crystal system	orthorhombic
Space group	$P2_12_12_1$
a/Å	7.6390(4)
b/Å	12.0399(6)
c/Å	18.6843(10)
α/°	90
β/°	90
γ/°	90
Volume/Å^3	1718.45(15)
Z	4
ρ_{calc}g/cm^3	1.462
μ/mm^{-1}	1.078
F(000)	796.0
Crystal size/mm^3	0.213 × 0.15 × 0.078
Radiation	MoKα (λ = 0.71073)
2Θ range for data collection/°	4.024 to 56.618
Index ranges	-10 ≤ h ≤ 10, -16 ≤ k ≤ 16, -24 ≤ l ≤ 24
Reflections collected	31559
Independent reflections	4259 [R_{int} = 0.0520, R_{sigma} = 0.0334]
Data/restraints/parameters	4259/0/192
Goodness-of-fit on F^2	1.180
Final R indexes [I>=2σ (I)]	R_1 = 0.0400, wR_2 = 0.0740
Final R indexes [all data]	R_1 = 0.0445, wR_2 = 0.0751
Largest diff. peak/hole / e Å^{-3}	0.37/-0.52
Flack parameter	0.32(3)

Table S9 Fractional Atomic Coordinates (×10[4]) and Equivalent Isotropic Displacement Parameters (Å[2]×10[3]) for [Mn(L3)Cl2]. U_{eq} is defined as 1/3 of of the trace of the orthogonalised U_{IJ} tensor.

Atom	*x*	*y*	*z*	U(eq)
Mn1	6086.4(6)	4980.8(5)	3785.8(2)	9.24(11)
Cl1	4011.2(13)	3448.4(7)	3963.8(5)	16.28(19)
Cl2	4114.4(14)	6443.6(8)	4170.3(5)	20.6(2)
N2	8383(4)	6164(3)	3551.7(17)	13.4(7)
N1	6014(4)	5155(2)	2511.1(14)	11.3(6)
N4	8286(4)	3859(2)	3335.3(16)	10.0(6)
N3	7985(4)	4826(3)	4744.7(15)	14.2(7)
C6	8662(5)	3175(3)	3972(2)	15.3(8)
C7	7362(5)	3272(3)	2759(2)	13.1(8)
C10	9862(5)	4435(3)	3063(2)	14.7(8)
C11	4138(5)	5357(3)	2332.7(19)	14.5(7)
C2	7610(5)	6838(3)	2978(2)	15.5(8)
C8	6642(5)	4088(3)	2195(2)	13.7(8)
C1	7145(5)	6132(3)	2330(2)	14.3(8)
C5	9255(5)	3907(3)	4590(2)	16.7(8)
C12	3720(5)	5496(3)	1549(2)	16.6(7)
C4	8871(6)	5923(3)	4854(2)	21.2(8)
C3	8557(5)	6751(3)	4239(2)	16.7(8)
C9	10057(5)	5639(3)	3335(2)	15.0(8)
C13	2575(5)	4883(4)	1202(2)	23.0(8)
C14	6892(6)	4530(4)	5370(2)	24.1(9)

Table S10 Anisotropic Displacement Parameters (Å[2]×10[3]) for [Mn(L3)Cl2]. The Anisotropic displacement factor exponent takes the form: $-2\pi^2[h^2a^{*2}U_{11}+2hka^*b^*U_{12}+…]$.

Atom	U_{11}	U_{22}	U_{33}	U_{23}	U_{13}	U_{12}
Mn1	9.2(2)	9.5(2)	9.0(2)	-0.9(2)	0.1(2)	-0.1(3)
Cl1	13.1(4)	15.4(4)	20.3(4)	1.8(3)	0.9(4)	-4.2(4)
Cl2	15.1(4)	19.9(5)	26.9(5)	-10.4(4)	-1.2(4)	4.9(4)
N2	15.3(16)	9.1(14)	15.7(16)	1.2(12)	-4.6(12)	0.4(12)
N1	12.4(12)	8.7(14)	12.6(13)	-0.6(11)	-1.8(12)	1.0(13)
N4	10.2(15)	8.4(14)	11.6(15)	-0.6(12)	0.5(11)	2.8(11)
N3	14.9(13)	16.2(18)	11.4(14)	-0.1(12)	0.2(11)	-1.1(13)
C6	14.3(18)	11.7(17)	20.0(19)	3.8(14)	0.6(15)	4.6(14)
C7	13.5(17)	11.0(18)	14.8(18)	-3.7(14)	2.3(14)	0.8(14)
C10	10.8(17)	18.4(19)	15.0(19)	-0.3(15)	3.3(15)	2.5(15)
C11	12.7(16)	16.5(16)	14.2(16)	1.0(13)	1.4(15)	2.1(13)
C2	16.1(18)	9.1(18)	21(2)	2.8(15)	-1.7(15)	-1.6(14)
C8	14.1(18)	13.1(18)	14.0(18)	-2.3(14)	1.1(14)	0.6(14)
C1	15.2(18)	12.2(18)	15.4(18)	4.6(15)	1.0(15)	-1.8(14)
C5	15.2(19)	17.9(18)	17.1(18)	4.2(15)	-1.7(15)	-0.6(15)
C12	12.3(17)	19.6(17)	17.8(18)	3.4(15)	0.9(15)	5.3(15)
C4	23(2)	18.4(18)	22.0(19)	-5.9(15)	-9.9(19)	-0.5(17)
C3	16.8(19)	12.2(17)	21.2(19)	-4.7(15)	-5.7(16)	-6.6(14)

Table S10 Anisotropic Displacement Parameters (Å^2×10^3) for [Mn(L3)Cl2]. The Anisotropic displacement factor exponent takes the form: $-2\pi^2[h^2a^{*2}U_{11}+2hka^*b^*U_{12}+…]$.

Atom	U_{11}	U_{22}	U_{33}	U_{23}	U_{13}	U_{12}
C9	9.5(17)	16.5(19)	19(2)	2.9(16)	0.2(15)	-2.6(15)
C13	20.4(16)	30(2)	18.6(18)	-2(2)	-0.6(15)	0.4(18)
C14	26(2)	33(2)	13.2(19)	2.4(17)	3.6(17)	-2.7(18)

Table S11 Bond Lengths for [Mn(L3)Cl2].

Atom	Atom	Length/Å	Atom	Atom	Length/Å
Mn1	Cl1	2.4552(11)	N4	C7	1.469(5)
Mn1	Cl2	2.4264(11)	N4	C10	1.480(5)
Mn1	N2	2.302(3)	N3	C5	1.500(5)
Mn1	N1	2.392(3)	N3	C4	1.498(5)
Mn1	N4	2.314(3)	N3	C14	1.479(5)
Mn1	N3	2.313(3)	C6	C5	1.521(5)
N2	C2	1.469(5)	C7	C8	1.543(5)
N2	C3	1.472(5)	C10	C9	1.544(5)
N2	C9	1.482(5)	C11	C12	1.507(5)
N1	C11	1.491(4)	C2	C1	1.521(5)
N1	C8	1.493(4)	C12	C13	1.316(5)
N1	C1	1.499(4)	C4	C3	1.541(5)
N4	C6	1.476(5)			

Table S12 Bond Angles for [Mn(L3)Cl2].

Atom	Atom	Atom	Angle/˚	Atom	Atom	Atom	Angle/˚
Cl2	Mn1	Cl1	96.00(3)	C1	N1	Mn1	106.3(2)
N2	Mn1	Cl1	169.38(9)	C6	N4	Mn1	100.0(2)
N2	Mn1	Cl2	94.62(9)	C6	N4	C10	112.4(3)
N2	Mn1	N1	76.95(10)	C7	N4	Mn1	101.5(2)
N2	Mn1	N4	74.85(10)	C7	N4	C6	114.6(3)
N2	Mn1	N3	73.68(11)	C7	N4	C10	111.3(3)
N1	Mn1	Cl1	100.71(7)	C10	N4	Mn1	116.2(2)
N1	Mn1	Cl2	102.52(7)	C5	N3	Mn1	108.4(2)
N4	Mn1	Cl1	94.55(8)	C4	N3	Mn1	108.5(2)
N4	Mn1	Cl2	169.14(8)	C4	N3	C5	112.6(3)
N4	Mn1	N1	72.90(10)	C14	N3	Mn1	106.1(2)
N3	Mn1	Cl1	103.86(8)	C14	N3	C5	109.9(3)
N3	Mn1	Cl2	102.62(8)	C14	N3	C4	111.1(3)
N3	Mn1	N1	142.48(10)	N4	C6	C5	110.2(3)
N3	Mn1	N4	77.26(11)	N4	C7	C8	111.4(3)
C2	N2	Mn1	100.0(2)	N4	C10	C9	113.9(3)
C2	N2	C3	114.0(3)	N1	C11	C12	116.0(3)
C2	N2	C9	112.5(3)	N2	C2	C1	111.5(3)
C3	N2	Mn1	101.6(2)	N1	C8	C7	113.1(3)
C3	N2	C9	111.4(3)	N1	C1	C2	113.1(3)

Table S12 Bond Angles for [Mn(L3)Cl2].

Atom	Atom	Atom	Angle/˚	Atom	Atom	Atom	Angle/˚
C9	N2	Mn1	116.5(2)	N3	C5	C6	112.4(3)
C11	N1	Mn1	105.02(19)	C13	C12	C11	123.9(4)
C11	N1	C8	111.2(3)	N3	C4	C3	113.5(3)
C11	N1	C1	112.1(3)	N2	C3	C4	110.7(3)
C8	N1	Mn1	108.1(2)	N2	C9	C10	114.0(3)
C8	N1	C1	113.6(3)				

Table S13 Torsion Angles for [Mn(L3)Cl2].

A	B	C	D	Angle/˚	A	B	C	D	Angle/˚
Mn1	N2	C2	C1	-61.6(3)	C7	N4	C10	C9	134.1(3)
Mn1	N2	C3	C4	57.3(3)	C10	N4	C6	C5	63.7(4)
Mn1	N2	C9	C10	18.9(4)	C10	N4	C7	C8	-64.2(4)
Mn1	N1	C11	C12	-179.8(2)	C11	N1	C8	C7	-122.2(3)
Mn1	N1	C8	C7	-7.4(3)	C11	N1	C1	C2	101.0(3)
Mn1	N1	C1	C2	-13.2(3)	C2	N2	C3	C4	163.9(3)
Mn1	N4	C6	C5	-60.3(3)	C2	N2	C9	C10	-95.7(4)
Mn1	N4	C7	C8	60.1(3)	C8	N1	C11	C12	-63.1(4)
Mn1	N4	C10	C9	18.6(4)	C8	N1	C1	C2	-132.0(3)
Mn1	N3	C5	C6	-14.8(3)	C1	N1	C11	C12	65.2(4)
Mn1	N3	C4	C3	-10.6(4)	C1	N1	C8	C7	110.3(3)
N2	C2	C1	N1	53.4(4)	C5	N3	C4	C3	109.4(4)
N1	C11	C12	C13	123.7(4)	C4	N3	C5	C6	-134.9(3)
N4	C6	C5	N3	53.7(4)	C3	N2	C2	C1	-169.2(3)
N4	C7	C8	N1	-36.0(4)	C3	N2	C9	C10	134.8(3)
N4	C10	C9	N2	-24.3(4)	C9	N2	C2	C1	62.7(4)
N3	C4	C3	N2	-32.4(5)	C9	N2	C3	C4	-67.4(4)
C6	N4	C7	C8	166.8(3)	C14	N3	C5	C6	100.7(4)
C6	N4	C10	C9	-95.8(4)	C14	N3	C4	C3	-126.9(4)
C7	N4	C6	C5	-167.9(3)					

Table S14 Hydrogen Atom Coordinates (Å×10[4]) and Isotropic Displacement Parameters (Å[2]×10[3]) for [Mn(L3)Cl2].

Atom	*x*	*y*	*z*	U(eq)
H6A	9589.32	2628.63	3857.79	18
H6B	7596.42	2760.35	4113.1	18
H7A	6381.19	2839.51	2964.84	16
H7B	8174.47	2743.7	2525.01	16
H10A	9817.28	4444.48	2533.03	18
H10B	10911.29	4006.9	3205.53	18
H11A	3750.24	6034.08	2588.3	17
H11B	3440.05	4727.76	2519.73	17
H2A	8449.96	7422.66	2833.51	19
H2B	6540.33	7207.96	3159.2	19

Table S14 Hydrogen Atom Coordinates (Å×10[4]) and Isotropic Displacement Parameters (Å[2]×10[3]) for [Mn(L3)Cl2].

Atom	*x*	*y*	*z*	U(eq)
H8A	7574.64	4250.99	1841.75	16
H8B	5662.5	3729.92	1936.06	16
H1A	6528.57	6601.7	1975.25	17
H1B	8238.84	5862.27	2104.82	17
H5A	9396.54	3444.65	5023.94	20
H5B	10410.36	4232.78	4472.66	20
H12	4320.06	6059.31	1291.38	20
H4A	10145.67	5797.5	4905.05	25
H4B	8443	6254.9	5306.1	25
H3A	7478.15	7181.93	4334.66	20
H3B	9549.08	7279.17	4212.57	20
H9A	10863.8	5642.14	3749.38	18
H9B	10597.05	6093.52	2952.36	18
H13A	1954.09	4313.51	1445.12	28
H13B	2368.13	5011.2	707.03	28
H14A	6000.82	5104.04	5443.72	36
H14B	7631.86	4474.93	5796.69	36
H14C	6318.73	3814.2	5282.78	36

Experimental

Single crystals of $C_{14}H_{28}Cl_2MnN_4$ **[[Mn(L3)Cl2]]** were []. A suitable crystal was selected and [] on a **Bruker PHOTON-II** diffractometer. The crystal was kept at 120.0 K during data collection. Using Olex2 [1], the structure was solved with the SHELXT [2] structure solution program using Intrinsic Phasing and refined with the SHELXL [3] refinement package using Least Squares minimisation.

1. Dolomanov, O.V., Bourhis, L.J., Gildea, R.J, Howard, J.A.K. & Puschmann, H. (2009), J. Appl. Cryst. 42, 339-341.
2. Sheldrick, G.M. (2015). Acta Cryst. A71, 3-8.
3. Sheldrick, G.M. (2015). Acta Cryst. C71, 3-8.

Crystal structure determination of [[Mn(L3)Cl2]]

Crystal Data for $C_{14}H_{28}Cl_2MnN_4$ (M =378.24 g/mol): orthorhombic, space group $P2_12_12_1$ (no. 19), a = 7.6390(4) Å, b = 12.0399(6) Å, c = 18.6843(10) Å, V = 1718.45(15) Å^3, Z = 4, T = 120.0 K, μ(MoKα) = 1.078 mm^{-1}, $Dcalc$ = 1.462 g/cm^3, 31559 reflections measured (4.024° ≤ 2Θ ≤ 56.618°), 4259 unique (R_{int} = 0.0520, R_{sigma} = 0.0334) which were used in all calculations. The final R_1 was 0.0400 (I > 2σ(I)) and wR_2 was 0.0751 (all data).

Refinement model description

Number of restraints - 0, number of constraints - unknown.

Details:

```
1. Twinned data refinement
 Scales: 0.68(3)
 0.32(3)
2. Fixed Uiso
 At 1.2 times of:
  All C(H) groups, All C(H,H) groups
 At 1.5 times of:
  All C(H,H,H) groups
3.a Secondary CH2 refined with riding coordinates:
 C6(H6A,H6B), C7(H7A,H7B), C10(H10A,H10B), C11(H11A,H11B), C2(H2A,H2B), C8(H8A,
 H8B), C1(H1A,H1B), C5(H5A,H5B), C4(H4A,H4B), C3(H3A,H3B), C9(H9A,H9B)
3.b Aromatic/amide H refined with riding coordinates:
 C12(H12)
3.c X=CH2 refined with riding coordinates:
 C13(H13A,H13B)
```

```
3.d Idealised Me refined as rotating group:
 C14(H14A,H14B,H14C)
```

This report has been created with Olex2, compiled on 2020.11.12 svn.r5f609507 for OlexSys. Please let us know if there are any errors or if you would like to have additional features.

[Fe(L3)Cl$_2$]

Table S15 Crystal data and structure refinement for [Fe(L3)Cl2].

Identification code	[Fe(L3)Cl2]
Empirical formula	$C_{14}H_{28}Cl_2FeN_4$
Formula weight	379.15
Temperature/K	120.00
Crystal system	orthorhombic
Space group	$P2_12_12_1$
a/Å	7.6198(5)
b/Å	11.9637(8)
c/Å	18.6306(14)
α/°	90
β/°	90
γ/°	90
Volume/Å^3	1698.4(2)
Z	4
ρ_{calc}g/cm^3	1.483
μ/mm^{-1}	1.202
F(000)	800.0
Crystal size/mm^3	0.287 × 0.134 × 0.127
Radiation	MoKα (λ = 0.71073)
2Θ range for data collection/°	4.046 to 56.798
Index ranges	-10 ≤ h ≤ 9, -15 ≤ k ≤ 15, -24 ≤ l ≤ 24
Reflections collected	41891
Independent reflections	4239 [R_{int} = 0.0400, R_{sigma} = 0.0206]
Data/restraints/parameters	4239/0/192
Goodness-of-fit on F^2	1.061
Final R indexes [I>=2σ (I)]	R_1 = 0.0384, wR_2 = 0.0919
Final R indexes [all data]	R_1 = 0.0400, wR_2 = 0.0930
Largest diff. peak/hole / e Å^{-3}	0.94/-0.58
Flack parameter	0.47(3)

Table S16 Fractional Atomic Coordinates (×10^4) and Equivalent Isotropic Displacement Parameters (Å^2×10^3) for [Fe(L3)Cl2]. U_{eq} is defined as 1/3 of of the trace of the orthogonalised U_{IJ} tensor.

Atom	*x*	*y*	*z*	U(eq)
Fe1	6185.4(6)	4988.9(5)	3777.3(2)	9.66(12)
Cl1	4083.5(14)	3492.8(9)	3952.1(6)	23.4(2)
Cl2	4176.0(15)	6404.8(10)	4157.2(7)	29.1(3)
N2	8387(5)	6174(3)	3557(2)	19.2(8)

Table S16 Fractional Atomic Coordinates (×10⁴) and Equivalent Isotropic Displacement Parameters (Å²×10³) for [Fe(L3)Cl2]. U_{eq} is defined as 1/3 of of the trace of the orthogonalised U_{IJ} tensor.

Atom	*x*	*y*	*z*	U(eq)
N1	6067(4)	5129(3)	2536.5(16)	15.5(6)
N4	8305(5)	3863(3)	3375(2)	17.3(7)
N3	7930(4)	4858(4)	4752.9(17)	21.3(8)
C6	8676(6)	3173(3)	4011(2)	21.3(9)
C7	7435(6)	3260(4)	2787(3)	21.1(10)
C10	9907(6)	4436(4)	3105(3)	22.3(9)
C11	4184(5)	5354(4)	2363(2)	19.6(8)
C2	7622(6)	6850(4)	2976(3)	22.3(10)
C8	6712(7)	4072(4)	2217(2)	22.3(10)
C1	7167(7)	6121(4)	2338(2)	21.8(9)
C5	9222(6)	3909(4)	4621(3)	25.5(10)
C12	3770(6)	5482(4)	1574(2)	23.0(8)
C4	8825(8)	5940(4)	4874(3)	30.8(11)
C3	8545(7)	6771(4)	4246(3)	25.6(10)
C9	10070(6)	5643(4)	3353(3)	23.6(10)
C13	2590(5)	4899(5)	1233(2)	28.5(10)
C14	6780(7)	4535(5)	5354(2)	31.1(11)

Table S17 Anisotropic Displacement Parameters (Å²×10³) for [Fe(L3)Cl2]. The Anisotropic displacement factor exponent takes the form: $-2\pi^2[h^2a^{*2}U_{11}+2hka^*b^*U_{12}+\ldots]$.

Atom	U_{11}	U_{22}	U_{33}	U_{23}	U_{13}	U_{12}
Fe1	7.83(19)	11.8(2)	9.3(2)	-0.4(2)	0.57(16)	0.4(2)
Cl1	13.8(5)	21.0(5)	35.3(6)	8.9(4)	1.7(4)	-5.0(4)
Cl2	15.5(5)	27.9(5)	43.9(6)	-20.2(5)	0.5(4)	5.6(4)
N2	13.4(17)	11.0(15)	33(2)	5.2(14)	-3.1(14)	-1.7(13)
N1	11.7(13)	21.3(17)	13.4(13)	1.0(12)	-2.6(11)	3.2(15)
N4	12.8(17)	14.0(16)	25.0(18)	1.7(13)	3.1(14)	0.9(13)
N3	16.6(14)	36(2)	11.0(14)	1.1(15)	-0.1(11)	-1.5(17)
C6	19(2)	15.0(18)	30(2)	3.5(16)	-4.3(17)	4.4(16)
C7	18(2)	15(2)	30(2)	-7.4(17)	4.3(17)	0.9(16)
C10	11.0(19)	23(2)	33(2)	-1.0(19)	8.1(18)	2.6(16)
C11	12.4(17)	27(2)	19.4(18)	2.0(15)	-1.0(15)	2.8(14)
C2	20(2)	13(2)	34(3)	6.3(18)	0.6(18)	-2.0(15)
C8	29(2)	20(2)	18(2)	-6.3(16)	5.4(17)	-3.5(18)
C1	25(2)	21(2)	19(2)	10.0(17)	7.9(17)	4.5(18)
C5	24(2)	22(2)	30(2)	8.1(18)	-10.8(19)	-2.6(18)
C12	17.3(19)	31(2)	20.3(19)	5.3(17)	0.1(17)	5.4(17)
C4	35(3)	24(2)	34(2)	-10.6(19)	-21(2)	0(2)
C3	25(2)	15.6(19)	36(2)	-8.9(18)	-10(2)	-3.9(17)
C9	13.0(19)	22(2)	36(3)	9.0(19)	1.5(18)	-3.5(17)
C13	20.8(18)	44(3)	20.8(18)	-4(2)	-3.1(15)	0(2)
C14	29(2)	48(3)	16(2)	-0.1(19)	3.9(18)	-2(2)

Table S18 Bond Lengths for [Fe(L3)Cl2].

Atom	Atom	Length/Å	Atom	Atom	Length/Å
Fe1	Cl1	2.4238(12)	N4	C7	1.470(6)
Fe1	Cl2	2.3905(12)	N4	C10	1.487(6)
Fe1	N2	2.235(4)	N3	C5	1.523(6)
Fe1	N1	2.320(3)	N3	C4	1.480(6)
Fe1	N4	2.233(4)	N3	C14	1.474(6)
Fe1	N3	2.257(3)	C6	C5	1.496(6)
N2	C2	1.471(6)	C7	C8	1.541(7)
N2	C3	1.474(6)	C10	C9	1.521(6)
N2	C9	1.481(6)	C11	C12	1.510(6)
N1	C11	1.495(5)	C2	C1	1.515(7)
N1	C8	1.481(5)	C12	C13	1.303(7)
N1	C1	1.500(6)	C4	C3	1.550(7)
N4	C6	1.471(5)			

Table S19 Bond Angles for [Fe(L3)Cl2].

Atom	Atom	Atom	Angle/˚	Atom	Atom	Atom	Angle/˚
Cl2	Fe1	Cl1	93.46(4)	C1	N1	Fe1	106.4(2)
N2	Fe1	Cl1	171.63(10)	C6	N4	Fe1	101.9(3)
N2	Fe1	Cl2	94.90(10)	C6	N4	C10	111.9(4)
N2	Fe1	N1	78.50(13)	C7	N4	Fe1	102.7(3)
N2	Fe1	N3	75.50(14)	C7	N4	C6	114.4(3)
N1	Fe1	Cl1	99.29(9)	C7	N4	C10	110.1(4)
N1	Fe1	Cl2	102.64(9)	C10	N4	Fe1	115.4(3)
N4	Fe1	Cl1	94.44(10)	C5	N3	Fe1	107.6(3)
N4	Fe1	Cl2	171.99(10)	C4	N3	Fe1	109.5(3)
N4	Fe1	N2	77.20(11)	C4	N3	C5	112.3(3)
N4	Fe1	N1	74.77(13)	C14	N3	Fe1	106.3(3)
N4	Fe1	N3	78.60(14)	C14	N3	C5	108.1(4)
N3	Fe1	Cl1	103.28(11)	C14	N3	C4	112.8(4)
N3	Fe1	Cl2	100.83(11)	N4	C6	C5	109.6(3)
N3	Fe1	N1	146.15(12)	N4	C7	C8	111.4(3)
C2	N2	Fe1	100.7(3)	N4	C10	C9	113.7(4)
C2	N2	C3	114.0(3)	N1	C11	C12	115.4(3)
C2	N2	C9	112.9(4)	N2	C2	C1	110.7(4)
C3	N2	Fe1	102.1(3)	N1	C8	C7	112.3(3)
C3	N2	C9	111.2(4)	N1	C1	C2	112.9(3)
C9	N2	Fe1	115.1(3)	C6	C5	N3	112.4(4)
C11	N1	Fe1	105.5(2)	C13	C12	C11	124.4(4)
C11	N1	C1	109.9(3)	N3	C4	C3	112.5(4)
C8	N1	Fe1	109.0(2)	N2	C3	C4	111.0(3)
C8	N1	C11	112.7(3)	N2	C9	C10	114.5(4)
C8	N1	C1	113.0(3)				

Table S20 Torsion Angles for [Fe(L3)Cl2].

A	B	C	D	Angle/˚	A	B	C	D	Angle/˚
Fe1	N2	C2	C1	-60.1(4)	C7	N4	C10	C9	133.4(4)
Fe1	N2	C3	C4	55.5(4)	C10	N4	C6	C5	65.1(5)
Fe1	N2	C9	C10	17.8(5)	C10	N4	C7	C8	-65.9(4)
Fe1	N1	C11	C12	180.0(3)	C11	N1	C8	C7	-122.6(4)
Fe1	N1	C8	C7	-5.8(4)	C11	N1	C1	C2	100.2(4)
Fe1	N1	C1	C2	-13.5(4)	C2	N2	C3	C4	163.2(4)
Fe1	N4	C6	C5	-58.7(4)	C2	N2	C9	C10	-97.1(4)
Fe1	N4	C7	C8	57.5(4)	C8	N1	C11	C12	-61.2(5)
Fe1	N4	C10	C9	17.7(5)	C8	N1	C1	C2	-133.0(4)
Fe1	N3	C5	C6	-15.3(4)	C1	N1	C11	C12	65.7(5)
Fe1	N3	C4	C3	-8.8(5)	C1	N1	C8	C7	112.2(4)
N2	C2	C1	N1	51.9(5)	C5	N3	C4	C3	110.6(4)
N1	C11	C12	C13	125.1(5)	C4	N3	C5	C6	-135.8(4)
N4	C6	C5	N3	51.6(5)	C3	N2	C2	C1	-168.6(4)
N4	C7	C8	N1	-34.8(5)	C3	N2	C9	C10	133.2(4)
N4	C10	C9	N2	-23.1(6)	C9	N2	C2	C1	63.2(5)
N3	C4	C3	N2	-32.1(6)	C9	N2	C3	C4	-67.7(5)
C6	N4	C7	C8	167.1(4)	C14	N3	C5	C6	99.1(4)
C6	N4	C10	C9	-98.2(4)	C14	N3	C4	C3	-126.9(4)
C7	N4	C6	C5	-168.7(4)					

Table S21 Hydrogen Atom Coordinates (Å×10^4) and Isotropic Displacement Parameters (Å^2×10^3) for [Fe(L3)Cl2].

Atom	*x*	*y*	*z*	U(eq)
H6A	9624.95	2634.77	3900.64	26
H6B	7614.8	2745.34	4146.21	26
H7A	6458.5	2807.81	2983.47	25
H7B	8283.34	2743.58	2558.84	25
H10A	9896.51	4419.85	2574.01	27
H10B	10952.66	4016.18	3268.63	27
H11A	3822.52	6046.9	2612.95	24
H11B	3466.17	4734.85	2556.95	24
H2A	8470.27	7432.05	2827.35	27
H2B	6549.54	7227.51	3152.37	27
H8A	7650.42	4244.28	1866.16	27
H8B	5739.91	3705.78	1954.65	27
H1A	6526.09	6576.34	1979.68	26
H1B	8266.44	5856.88	2110.74	26
H5A	10393.4	4227.06	4516.9	31
H5B	9321.08	3451.65	5062.41	31
H12	4412.88	6022.57	1309.08	28
H4A	10098.17	5805.09	4935.54	37
H4B	8376.82	6278.2	5322.56	37

Table S21 Hydrogen Atom Coordinates (Å×10^4) and Isotropic Displacement Parameters (Å^2×10^3) for [Fe(L3)Cl2].

Atom	*x*	*y*	*z*	U(eq)
H3A	7466.55	7212.02	4333.25	31
H3B	9548.01	7295.78	4223.58	31
H9A	10873	5669.89	3770.39	28
H9B	10611.08	6086.3	2962.7	28
H13A	1924.17	4350.7	1482.18	34
H13B	2396.17	5021.72	735.79	34
H14A	5912.91	5125.96	5438.65	47
H14B	7487.9	4428.14	5788.08	47
H14C	6173	3836.49	5236.46	47

Experimental

Single crystals of $C_{14}H_{28}Cl_2FeN_4$ **[[Fe(L3)Cl2]]** were []. A suitable crystal was selected and [] on a **Bruker PHOTON-II** diffractometer. The crystal was kept at 120.00 K during data collection. Using Olex2 [1], the structure was solved with the SHELXT [2] structure solution program using Intrinsic Phasing and refined with the SHELXL [3] refinement package using Least Squares minimisation.

1. Dolomanov, O.V., Bourhis, L.J., Gildea, R.J, Howard, J.A.K. & Puschmann, H. (2009), J. Appl. Cryst. 42, 339-341.
2. Sheldrick, G.M. (2015). Acta Cryst. A71, 3-8.
3. Sheldrick, G.M. (2015). Acta Cryst. C71, 3-8.

Crystal structure determination of [[Fe(L3)Cl2]]

Crystal Data for $C_{14}H_{28}Cl_2FeN_4$ (M =379.15 g/mol): orthorhombic, space group P2$_1$2$_1$2$_1$ (no. 19), a = 7.6198(5) Å, b = 11.9637(8) Å, c = 18.6306(14) Å, V = 1698.4(2) Å^3, Z = 4, T = 120.00 K, μ(MoKα) = 1.202 mm^{-1}, $Dcalc$ = 1.483 g/cm^3, 41891 reflections measured (4.046° ≤ 2Θ ≤ 56.798°), 4239 unique (R_{int} = 0.0400, R_{sigma} = 0.0206) which were used in all calculations. The final R_1 was 0.0384 (I > 2σ(I)) and wR_2 was 0.0930 (all data).

Refinement model description

Number of restraints - 0, number of constraints - unknown.

Details:

```
1. Twinned data refinement
 Scales: 0.53(3)
 0.47(3)
2. Fixed Uiso
 At 1.2 times of:
  All C(H) groups, All C(H,H) groups
 At 1.5 times of:
  All C(H,H,H) groups
3.a Secondary CH2 refined with riding coordinates:
 C6(H6A,H6B), C7(H7A,H7B), C10(H10A,H10B), C11(H11A,H11B), C2(H2A,H2B), C8(H8A,
 H8B), C1(H1A,H1B), C5(H5A,H5B), C4(H4A,H4B), C3(H3A,H3B), C9(H9A,H9B)
3.b Aromatic/amide H refined with riding coordinates:
 C12(H12)
3.c X=CH2 refined with riding coordinates:
 C13(H13A,H13B)
3.d Idealised Me refined as rotating group:
 C14(H14A,H14B,H14C)
```

This report has been created with Olex2, compiled on 2020.11.12 svn.r5f609507 for OlexSys. Please let us know if there are any errors or if you would like to have additional features.

L8-precursor

Table S22 Crystal data and structure refinement for L8-precursor.

Identification code	L8-precursor
Empirical formula	$C_{20}H_{34}I_2N_4O_2$
Formula weight	616.31

Temperature/K	150
Crystal system	monoclinic
Space group	$P2_1/n$
a/Å	9.5389(10)
b/Å	16.9155(17)
c/Å	14.7768(14)
α/°	90
β/°	91.479(4)
γ/°	90
Volume/$Å^3$	2383.5(4)
Z	4
$\rho_{calc}g/cm^3$	1.717
μ/mm^{-1}	3.350
F(000)	1216.0
Crystal size/mm^3	0.05 × 0.02 × 0.01
Radiation	synchrotron (λ = 0.7749)
2Θ range for data collection/°	3.992 to 52.228
Index ranges	-10 ≤ h ≤ 10, -19 ≤ k ≤ 19, -16 ≤ l ≤ 16
Reflections collected	37933
Independent reflections	3652 [R_{int} = 0.1904, R_{sigma} = 0.0979]
Data/restraints/parameters	3652/0/269
Goodness-of-fit on F^2	1.037
Final R indexes [I>=2σ (I)]	R_1 = 0.0617, wR_2 = 0.1579
Final R indexes [all data]	R_1 = 0.0820, wR_2 = 0.1757
Largest diff. peak/hole / e $Å^{-3}$	1.03/-0.74

Table S23 Fractional Atomic Coordinates (×10^4) and Equivalent Isotropic Displacement Parameters ($Å^2$×10^3) for L8-precursor. U_{eq} is defined as 1/3 of of the trace of the orthogonalised U_{IJ} tensor.

Atom	*x*	*y*	*z*	U(eq)
I1	-1606.2(17)	5768.0(4)	2280.9(12)	74.5(5)
I2	1221.0(7)	2067.0(9)	4507.7(5)	55.8(4)
O1	1226(9)	4926(5)	3641(6)	72(3)
O2	1327(12)	4027(5)	5238(6)	109(3)
N1	3579(7)	3694(4)	2204(4)	47.0(15)
N2	2035(7)	2318(4)	1576(4)	46.3(15)
N3	-147(7)	3009(4)	1621(5)	52.5(17)
N4	2060(7)	3957(4)	897(5)	51.9(17)
C1	4270(9)	3027(5)	1713(6)	53(2)
C2	3499(9)	2266(5)	1848(6)	55(2)
C3	1364(9)	1554(5)	1766(7)	58(2)
C4	-115(9)	1557(5)	1375(7)	62(2)
C5	-912(10)	2234(5)	1774(7)	64(2)
C6	-106(10)	3208(6)	647(7)	66(2)
C7	651(10)	3978(5)	502(6)	64(2)

Table S23 Fractional Atomic Coordinates (×10⁴) and Equivalent Isotropic Displacement Parameters (Å²×10³) for L8-precursor. U_{eq} is defined as 1/3 of of the trace of the orthogonalised U_{IJ} tensor.

Atom	*x*	*y*	*z*	U(eq)
C8	2721(11)	4736(5)	734(6)	67(2)
C9	4198(10)	4710(5)	1047(6)	65(2)
C10	4331(10)	4470(5)	2041(6)	57(2)
C11	2080(8)	3748(4)	1860(5)	46.8(19)
C12	1324(9)	2964(4)	2015(6)	48.5(19)
C13	3615(9)	3555(5)	3237(5)	53(2)
C14	5045(9)	3573(5)	3673(5)	54(2)
C15	5527(13)	4268(6)	4058(6)	72(3)
C16	6845(14)	4293(8)	4461(7)	83(3)
C17	7678(12)	3637(9)	4505(7)	83(3)
C18	7186(11)	2939(7)	4138(7)	75(3)
C19	5886(10)	2905(5)	3719(6)	60(2)
C20	-981(10)	3608(5)	2143(8)	73(3)
I2A	1278(14)	2449(15)	4674(10)	55.8(4)
I1A	-2123(19)	5732(6)	1885(14)	74.5(5)
O1A	400(40)	5230(20)	3610(30)	72(3)

Table S24 Anisotropic Displacement Parameters (Å²×10³) for L8-precursor. The Anisotropic displacement factor exponent takes the form: $-2\pi^2[h^2a^{*2}U_{11}+2hka^*b^*U_{12}+\ldots]$.

Atom	U_{11}	U_{22}	U_{33}	U_{23}	U_{13}	U_{12}
I1	89.3(8)	48.5(4)	86.2(10)	-1.3(4)	9.9(7)	9.8(4)
I2	63.3(4)	52.9(8)	51.5(4)	5.2(4)	9.0(3)	-4.5(3)
O1	66(6)	60(5)	89(6)	-13(4)	-17(5)	3(4)
O2	135(7)	87(5)	103(6)	-13(5)	-4(6)	-9(6)
N1	55(4)	47(4)	39(4)	6(3)	9(3)	-6(3)
N2	52(4)	38(3)	49(4)	-2(3)	4(3)	2(3)
N3	55(4)	38(4)	65(5)	-3(3)	12(3)	1(3)
N4	56(4)	48(4)	52(4)	11(3)	5(3)	-1(3)
C1	59(5)	56(5)	44(5)	6(4)	12(4)	7(4)
C2	65(6)	51(5)	49(5)	-5(4)	5(4)	4(4)
C3	64(6)	38(4)	72(6)	1(4)	6(4)	1(4)
C4	61(6)	46(5)	79(6)	-12(4)	8(5)	-5(4)
C5	60(6)	54(5)	78(7)	-11(5)	7(5)	-9(4)
C6	61(6)	70(6)	67(6)	3(5)	-1(5)	7(5)
C7	74(6)	59(5)	59(6)	20(5)	4(5)	10(5)
C8	85(7)	62(6)	54(5)	20(4)	6(5)	3(5)
C9	84(7)	56(5)	56(6)	22(4)	14(5)	-14(5)
C10	72(6)	45(4)	53(5)	7(4)	8(4)	-11(4)
C11	60(5)	37(4)	45(5)	4(3)	18(4)	2(3)
C12	55(5)	42(4)	48(5)	-5(3)	5(4)	0(3)
C13	63(5)	51(5)	46(5)	3(4)	1(4)	1(4)
C14	73(6)	43(5)	45(5)	1(4)	16(4)	-8(4)
C15	99(8)	62(6)	53(6)	6(4)	3(6)	-18(5)

Table S24 Anisotropic Displacement Parameters (Å^2×10^3) for L8-precursor. The Anisotropic displacement factor exponent takes the form: $-2\pi^2[h^2a^{*2}U_{11}+2hka^*b^*U_{12}+\ldots]$.

Atom	U_{11}	U_{22}	U_{33}	U_{23}	U_{13}	U_{12}
C16	108(9)	89(8)	53(6)	3(5)	-2(6)	-43(7)
C17	75(7)	128(11)	45(6)	-4(6)	1(5)	-30(7)
C18	66(7)	98(8)	61(6)	17(6)	11(5)	-2(6)
C19	66(6)	65(6)	49(5)	-1(4)	5(4)	-8(4)
C20	66(6)	46(5)	107(8)	-19(5)	30(6)	2(4)
I2A	63.3(4)	52.9(8)	51.5(4)	5.2(4)	9.0(3)	-4.5(3)
I1A	89.3(8)	48.5(4)	86.2(10)	-1.3(4)	9.9(7)	9.8(4)
O1A	66(6)	60(5)	89(6)	-13(4)	-17(5)	3(4)

Table S25 Bond Lengths for L8-precursor.

Atom	Atom	Length/Å	Atom	Atom	Length/Å
N1	C1	1.503(10)	C1	C2	1.499(12)
N1	C10	1.517(10)	C3	C4	1.510(12)
N1	C11	1.508(10)	C4	C5	1.503(12)
N1	C13	1.544(10)	C6	C7	1.507(13)
N2	C2	1.446(11)	C8	C9	1.472(14)
N2	C3	1.472(10)	C9	C10	1.525(12)
N2	C12	1.449(10)	C11	C12	1.530(11)
N3	C5	1.520(11)	C13	C14	1.493(12)
N3	C6	1.481(12)	C14	C15	1.380(12)
N3	C12	1.506(11)	C14	C19	1.386(13)
N3	C20	1.512(11)	C15	C16	1.377(16)
N4	C7	1.452(11)	C16	C17	1.366(17)
N4	C8	1.484(12)	C17	C18	1.376(15)
N4	C11	1.465(10)	C18	C19	1.373(14)

Table S26 Bond Angles for L8-precursor.

Atom	Atom	Atom	Angle/˚	Atom	Atom	Atom	Angle/˚
C1	N1	C10	111.0(6)	C4	C5	N3	110.4(7)
C1	N1	C11	107.8(6)	N3	C6	C7	111.0(8)
C1	N1	C13	111.3(6)	N4	C7	C6	111.4(7)
C10	N1	C13	106.9(6)	C9	C8	N4	109.2(7)
C11	N1	C10	110.0(6)	C8	C9	C10	111.5(7)
C11	N1	C13	109.8(6)	N1	C10	C9	110.8(7)
C2	N2	C3	108.3(6)	N1	C11	C12	110.1(6)
C2	N2	C12	112.5(6)	N4	C11	N1	109.3(6)
C12	N2	C3	111.4(6)	N4	C11	C12	111.1(7)
C6	N3	C5	111.4(7)	N2	C12	N3	107.9(6)
C6	N3	C12	109.9(6)	N2	C12	C11	111.0(7)
C6	N3	C20	111.8(7)	N3	C12	C11	109.7(6)
C12	N3	C5	110.2(6)	C14	C13	N1	114.9(7)
C12	N3	C20	109.5(7)	C15	C14	C13	119.1(8)

Table S26 Bond Angles for L8-precursor.

Atom	Atom	Atom	Angle/˚	Atom	Atom	Atom	Angle/˚
C20	N3	C5	103.9(7)	C15	C14	C19	119.2(9)
C7	N4	C8	107.8(7)	C19	C14	C13	121.7(8)
C7	N4	C11	112.6(6)	C16	C15	C14	119.7(11)
C11	N4	C8	112.1(7)	C17	C16	C15	121.3(10)
C2	C1	N1	111.0(6)	C16	C17	C18	119.1(11)
N2	C2	C1	112.5(7)	C19	C18	C17	120.6(11)
N2	C3	C4	109.3(7)	C18	C19	C14	120.2(9)
C5	C4	C3	109.3(7)				

Table S27 Torsion Angles for L8-precursor.

A	B	C	D	Angle/˚	A	B	C	D	Angle/˚
N1	C1	C2	N2	56.1(9)	C8	N4	C11	C12	176.9(7)
N1	C11	C12	N2	-57.7(8)	C8	C9	C10	N1	54.5(10)
N1	C11	C12	N3	-176.8(6)	C10	N1	C1	C2	-178.1(7)
N1	C13	C14	C15	95.2(9)	C10	N1	C11	N4	57.2(8)
N1	C13	C14	C19	-86.3(10)	C10	N1	C11	C12	179.5(6)
N2	C3	C4	C5	-57.7(10)	C10	N1	C13	C14	-54.7(9)
N3	C6	C7	N4	56.5(10)	C11	N1	C1	C2	-57.6(8)
N4	C8	C9	C10	-56.3(10)	C11	N1	C10	C9	-54.0(9)
N4	C11	C12	N2	63.6(9)	C11	N1	C13	C14	-174.0(6)
N4	C11	C12	N3	-55.5(8)	C11	N4	C7	C6	-55.1(10)
C1	N1	C10	C9	65.2(9)	C11	N4	C8	C9	60.9(9)
C1	N1	C11	N4	-64.0(7)	C12	N2	C2	C1	-54.4(9)
C1	N1	C11	C12	58.4(7)	C12	N2	C3	C4	63.2(9)
C1	N1	C13	C14	66.6(9)	C12	N3	C5	C4	-56.7(10)
C2	N2	C3	C4	-172.6(7)	C12	N3	C6	C7	-57.8(9)
C2	N2	C12	N3	175.0(6)	C13	N1	C1	C2	63.0(9)
C2	N2	C12	C11	54.8(9)	C13	N1	C10	C9	-173.2(7)
C3	N2	C2	C1	-177.9(7)	C13	N1	C11	N4	174.5(6)
C3	N2	C12	N3	-63.3(8)	C13	N1	C11	C12	-63.2(8)
C3	N2	C12	C11	176.6(7)	C13	C14	C15	C16	-179.9(8)
C3	C4	C5	N3	55.3(11)	C13	C14	C19	C18	-178.9(8)
C5	N3	C6	C7	179.8(7)	C14	C15	C16	C17	-1.4(16)
C5	N3	C12	N2	59.2(8)	C15	C14	C19	C18	-0.4(13)
C5	N3	C12	C11	-179.8(7)	C15	C16	C17	C18	0.0(16)
C6	N3	C5	C4	65.5(10)	C16	C17	C18	C19	1.2(15)
C6	N3	C12	N2	-63.9(8)	C17	C18	C19	C14	-1.0(14)
C6	N3	C12	C11	57.1(8)	C19	C14	C15	C16	1.6(14)
C7	N4	C8	C9	-174.5(7)	C20	N3	C5	C4	-174.0(8)
C7	N4	C11	N1	176.8(6)	C20	N3	C6	C7	64.1(10)
C7	N4	C11	C12	55.1(9)	C20	N3	C12	N2	173.0(7)
C8	N4	C7	C6	-179.4(8)	C20	N3	C12	C11	-66.1(8)
C8	N4	C11	N1	-61.4(8)					

Table S28 Hydrogen Atom Coordinates (Å×10⁴) and Isotropic Displacement Parameters (Å²×10³) for L8-precursor.

Atom	*x*	*y*	*z*	U(eq)
H2C	1331.05	4182.93	5785.45	163
H2D	1274.6	3525.8	5222.6	163
H1A	4289.32	3150.11	1058.12	64
H1B	5250.65	2969.01	1938.95	64
H2A	3949.7	1843.76	1493.48	66
H2B	3570.51	2115.63	2495.09	66
H3A	1347.43	1466.31	2427.82	69
H3B	1906.34	1120.35	1492.64	69
H4A	-95.34	1613.24	708.92	74
H4B	-583.72	1051.77	1517.23	74
H5A	-1016.23	2147.04	2431.01	77
H5B	-1861.45	2261.41	1488.42	77
H6A	-1075.62	3249.15	395.69	79
H6B	375.72	2780.02	319.68	79
H7A	700.69	4084.47	-155.06	77
H7B	118.22	4413.52	779.58	77
H8A	2666.86	4863.34	79.92	81
H8B	2217.49	5153.67	1064.72	81
H9A	4628.42	5236.63	966.72	78
H9B	4714.29	4326.51	674.5	78
H10A	5334.42	4413.7	2216.4	68
H10B	3923.45	4887.71	2422.1	68
H11	1594.48	4174.07	2201.13	56
H12	1286.8	2857.36	2679.77	58
H13A	3031.59	3963.36	3523.48	64
H13B	3184.46	3034.43	3359.41	64
H15	4953.73	4727.35	4044.71	86
H16	7180.91	4776.23	4712.4	100
H17	8584.72	3661.41	4785	99
H18	7750.03	2477.25	4175.88	90
H19	5561.87	2422.7	3459.32	72
H20A	-901.95	3490.17	2791.15	109
H20B	-1968.61	3583.17	1944.07	109
H20C	-614.68	4139.43	2030.34	109

Table S29 Atomic Occupancy for L8-precursor.

Atom	*Occupancy*	Atom	*Occupancy*	Atom	*Occupancy*
I1	0.921(5)	I2	0.948(4)	O1	0.807(14)
I2A	0.052(4)	I1A	0.079(5)	O1A	0.193(14)

Experimental

Single crystals of $C_{20}H_{34}I_2N_4O_2$ **[L8-precursor]** were **[]**. A suitable crystal was selected and **[]** on a diffractometer. The crystal was kept at 150 K during data collection. Using Olex2 [1], the structure was solved

with the SHELXT [2] structure solution program using Intrinsic Phasing and refined with the SHELXL [3] refinement package using Least Squares minimisation.

1. Dolomanov, O.V., Bourhis, L.J., Gildea, R.J, Howard, J.A.K. & Puschmann, H. (2009), J. Appl. Cryst. 42, 339-341.
2. Sheldrick, G.M. (2015). Acta Cryst. A71, 3-8.
3. Sheldrick, G.M. (2015). Acta Cryst. C71, 3-8.

Crystal structure determination of [L8-precursor]

Crystal Data for $C_{20}H_{34}I_2N_4O_2$ (M=616.31 g/mol): monoclinic, space group P2$_1$/n (no. 14), a = 9.5389(10) Å, b = 16.9155(17) Å, c = 14.7768(14) Å, β = 91.479(4)°, V = 2383.5(4) Å^3, Z = 4, T = 150 K, μ(synchrotron) = 3.350 mm^{-1}, $Dcalc$ = 1.717 g/cm^3, 37933 reflections measured (3.992° ≤ 2Θ ≤ 52.228°), 3652 unique (R_{int} = 0.1904, R_{sigma} = 0.0979) which were used in all calculations. The final R_1 was 0.0617 (I > 2σ(I)) and wR_2 was 0.1757 (all data).

Refinement model description

Number of restraints - 0, number of constraints - unknown.

Details:

```
1. Fixed Uiso
 At 1.2 times of:
  All C(H) groups, All C(H,H) groups
 At 1.5 times of:
  All C(H,H,H) groups, All O(H,H) groups
2. Uiso/Uaniso restraints and constraints
Uanis(I2A) = Uanis(I2)
Uanis(I1A) = Uanis(I1)
Uanis(O1A) = Uanis(O1)
3. Others
 Sof(I2A)=1-FVAR(1)
 Sof(I2)=FVAR(1)
 Sof(I1A)=1-FVAR(2)
 Sof(I1)=FVAR(2)
 Sof(O1A)=1-FVAR(3)
 Sof(O1)=FVAR(3)
4.a Free rotating group:
 O2(H2C,H2D)
4.b Ternary CH refined with riding coordinates:
 C11(H11), C12(H12)
4.c Secondary CH2 refined with riding coordinates:
 C1(H1A,H1B), C2(H2A,H2B), C3(H3A,H3B), C4(H4A,H4B), C5(H5A,H5B), C6(H6A,H6B),
 C7(H7A,H7B), C8(H8A,H8B), C9(H9A,H9B), C10(H10A,H10B), C13(H13A,H13B)
4.d Aromatic/amide H refined with riding coordinates:
 C15(H15), C16(H16), C17(H17), C18(H18), C19(H19)
4.e Idealised Me refined as rotating group:
 C20(H20A,H20B,H20C)
```

This report has been created with Olex2, compiled on 2020.11.12 svn.r5f609507 for OlexSys. Please let us know if there are any errors or if you would like to have additional features.

[Mn(L9)Cl$_2$]

Table S30 Crystal data and structure refinement for [Mn(L9)Cl2].

Identification code	[Mn(L9)Cl2]
Empirical formula	$C_{16}H_{30}Cl_2MnN_4$
Formula weight	404.28
Temperature/K	120
Crystal system	triclinic
Space group	P-1
a/Å	8.5540(11)
b/Å	13.2482(17)
c/Å	17.920(2)
α/°	107.492(2)
β/°	95.600(2)

γ/°	97.994(2)
Volume/Å³	1897.0(4)
Z	4
ρ_{calc}g/cm³	1.416
μ/mm⁻¹	0.982
F(000)	852.0
Crystal size/mm³	0.253 × 0.154 × 0.05
Radiation	Mo Kα (λ = 0.71073)
2Θ range for data collection/°	2.41 to 56.584
Index ranges	-11 ≤ h ≤ 11, -17 ≤ k ≤ 17, -23 ≤ l ≤ 23
Reflections collected	29491
Independent reflections	9415 [R_{int} = 0.0370, R_{sigma} = 0.0433]
Data/restraints/parameters	9415/41/437
Goodness-of-fit on F²	1.005
Final R indexes [I>=2σ (I)]	R_1 = 0.0307, wR_2 = 0.0591
Final R indexes [all data]	R_1 = 0.0499, wR_2 = 0.0645
Largest diff. peak/hole / e Å⁻³	0.33/-0.51

Table S31 Fractional Atomic Coordinates (×10⁴) and Equivalent Isotropic Displacement Parameters (Å²×10³) for [Mn(L9)Cl2]. U_{eq} is defined as 1/3 of of the trace of the orthogonalised U_{IJ} tensor.

Atom	*x*	*y*	*z*	U(eq)
Mn1	7920.4(3)	8362.4(2)	7883.3(2)	13.23(6)
Mn2	2915.8(3)	2990.6(2)	7116.7(2)	15.60(6)
Cl1	10749.1(5)	9097.0(3)	8302.6(2)	19.59(9)
Cl2	7557.1(5)	9291.8(3)	6906.2(2)	18.64(9)
Cl4	2221.8(5)	4600.1(3)	6919.1(2)	20.45(9)
Cl3	5556.3(6)	3763.7(4)	7906.3(3)	33.38(12)
N4	7821.6(17)	7371.3(11)	8738.2(8)	16.9(3)
N8	2964.5(17)	1312.5(11)	7214.0(8)	18.5(3)
N1	6949.4(16)	9484.7(11)	8943.9(8)	16.4(3)
N3	7833.7(17)	6588.2(11)	7119.3(8)	17.4(3)
N6	726.7(17)	1878.4(11)	6257.7(8)	16.9(3)
N2	5300.2(16)	7651.4(11)	7764.6(8)	16.3(3)
N5	1090.7(18)	2771.8(11)	7979.7(8)	20.3(3)
N7	3860.2(18)	2102.5(12)	5929.6(9)	22.8(3)
C7	8333(2)	8245.8(14)	9492.3(9)	20.0(4)
C6	9033(2)	6702.7(14)	8494.7(10)	21.3(4)
C3	5033(2)	6938.0(14)	6940.4(10)	19.2(4)
C11	7905(2)	10575.2(13)	9115.1(10)	18.7(4)
C5	8647(2)	6031.7(14)	7612.9(10)	21.6(4)
C23	944(2)	2126.9(15)	5527.4(10)	23.3(4)
C30	1581(2)	462.1(13)	6804.4(10)	20.0(4)
C14	8680(2)	6653.8(14)	6441.0(10)	22.0(4)
C29	607(2)	714.1(13)	6138.2(10)	20.7(4)
C10	6259(2)	6716.3(14)	8707.7(10)	21.5(4)

Table S31 Fractional Atomic Coordinates (×10^4) and Equivalent Isotropic Displacement Parameters (Å^2×10^3) for [Mn(L9)Cl2]. U_{eq} is defined as 1/3 of of the trace of the orthogonalised U_{IJ} tensor.

Atom	*x*	*y*	*z*	U(eq)
C4	6113(2)	6101.3(13)	6840.6(10)	19.8(4)
C9	4848(2)	7081.8(14)	8327.5(10)	20.6(4)
C8	7206(2)	9056.3(14)	9618.2(10)	20.5(4)
C27	3095(2)	1559.7(15)	8076.9(10)	25.9(4)
C2	4559(2)	8611.6(14)	7873.8(10)	20.0(4)
C22	-577(2)	2318.8(15)	6644.6(10)	21.4(4)
C28	1699(2)	2062.5(14)	8404.1(10)	25.9(4)
C12	7440(2)	11460.4(14)	9749.8(10)	21.9(4)
C21	-489(2)	2248.4(15)	7474.9(10)	23.0(4)
C1	5235.3(19)	9464.2(14)	8677.3(10)	20.6(4)
C26	4440(2)	1092.8(15)	6891.5(12)	27.5(4)
C25	4334(2)	1105.2(15)	6042.2(12)	29.5(5)
C15	8720(2)	5594.7(15)	5835.9(11)	27.6(4)
C16	7949(3)	5288.3(16)	5114.0(11)	32.1(5)
C24	2544(2)	1877.7(15)	5265.4(10)	27.7(4)
C31	869(2)	3810.7(15)	8549.3(11)	29.7(4)
C13	7021(2)	12330.0(15)	9639.9(12)	30.6(4)
C32	2356(3)	4444.3(15)	9071.3(11)	37.6(5)
C34	5125(9)	2749(6)	5703(3)	18.3(8)
C33	2497(4)	4779.5(17)	9851.5(13)	51.2(7)
C35	5696(6)	2253(4)	4928(3)	23.2(10)
C36	7176(5)	2470(3)	4812(2)	26.5(12)
C34A	5353(7)	2918(5)	5978(3)	18.3(8)
C35A	6310(5)	2619(3)	5302(3)	22.4(9)
C36A	6035(5)	1782(4)	4665(2)	27.7(11)

Table S32 Anisotropic Displacement Parameters (Å^2×10^3) for [Mn(L9)Cl2]. The Anisotropic displacement factor exponent takes the form: $-2\pi^2[h^2a^{*2}U_{11}+2hka^*b^*U_{12}+\ldots]$.

Atom	U_{11}	U_{22}	U_{33}	U_{23}	U_{13}	U_{12}
Mn1	11.87(13)	13.23(13)	15.29(12)	5.1(1)	3.9(1)	1.94(10)
Mn2	14.74(13)	13.00(13)	19.05(13)	7.26(10)	-0.38(10)	-0.45(10)
Cl1	13.0(2)	23.7(2)	23.8(2)	9.60(17)	4.68(16)	2.93(17)
Cl2	18.6(2)	18.7(2)	19.72(19)	9.07(16)	1.91(16)	1.02(17)
Cl4	22.4(2)	14.7(2)	25.2(2)	8.81(17)	1.55(17)	2.30(17)
Cl3	22.4(2)	23.8(2)	50.6(3)	17.4(2)	-12.8(2)	-6.7(2)
N4	19.8(8)	15.2(7)	17.4(7)	6.5(6)	5.5(6)	3.4(6)
N8	15.7(7)	16.9(7)	24.5(7)	10.4(6)	0.4(6)	0.7(6)
N1	14.1(7)	14.2(7)	19.6(7)	3.9(6)	3.9(6)	0.4(6)
N3	18.1(8)	16.8(7)	18.6(7)	6.3(6)	6.9(6)	3.5(6)
N6	18.9(8)	14.6(7)	16.9(7)	5.9(6)	1.2(6)	1.0(6)
N2	15.5(7)	14.5(7)	18.5(7)	4.0(6)	4.8(6)	2.2(6)
N5	28.0(9)	16.8(7)	15.4(7)	5.8(6)	3.5(6)	0.6(6)
N7	25.6(8)	17.1(8)	31.1(8)	12.3(6)	13.7(7)	4.9(7)

Table S32 Anisotropic Displacement Parameters (Å^2×10^3) for [Mn(L9)Cl2]. The Anisotropic displacement factor exponent takes the form: $-2\pi^2[h^2a^{*2}U_{11}+2hka^*b^*U_{12}+…]$.

Atom	U_{11}	U_{22}	U_{33}	U_{23}	U_{13}	U_{12}
C7	25.7(10)	21.0(9)	14.9(8)	7.5(7)	4.2(7)	4.6(8)
C6	25.0(10)	21.3(9)	22.9(9)	11.2(7)	6.8(7)	10.2(8)
C3	16.7(9)	18.7(9)	19.4(8)	3.7(7)	2.6(7)	-1.1(7)
C11	17.0(9)	16.5(9)	21.3(8)	4.2(7)	5.4(7)	0.7(7)
C5	26.5(10)	16.9(9)	24.8(9)	7.8(7)	8.7(8)	8.7(8)
C23	32.8(11)	20.5(9)	14.4(8)	5.7(7)	-3.1(7)	1.0(8)
C30	23.0(10)	12.8(8)	23.5(9)	7.0(7)	2.9(7)	-1.0(7)
C14	26.1(10)	22.6(9)	20.9(9)	9.1(7)	10.9(7)	6.4(8)
C29	24.6(10)	14.9(9)	19.0(8)	3.7(7)	1.2(7)	-3.0(7)
C10	27.9(10)	17.6(9)	21.8(9)	9.5(7)	9.8(8)	1.3(8)
C4	23.1(10)	14.0(8)	19.6(8)	2.0(7)	5.6(7)	0.2(7)
C9	20.5(9)	18.8(9)	21.1(9)	4.8(7)	9.6(7)	-2.3(7)
C8	23.4(10)	21.1(9)	17.0(8)	5.4(7)	8.0(7)	1.5(8)
C27	29.3(11)	22.7(10)	25.6(9)	14.9(8)	-8.6(8)	-2.5(8)
C2	11.8(8)	21.8(9)	27.1(9)	7.9(7)	4.0(7)	3.9(7)
C22	13.2(9)	22.3(9)	26.6(9)	7.4(8)	-1.7(7)	0.6(7)
C28	42.2(12)	18.2(9)	17.5(8)	9.7(7)	1.7(8)	-1.5(9)
C12	22.2(10)	17.3(9)	22.6(9)	1.5(7)	6.8(7)	0.2(8)
C21	20.3(10)	21.9(9)	26.8(9)	7.6(8)	8.3(8)	0.3(8)
C1	13.0(9)	17.4(9)	29.5(9)	3.6(7)	6.4(7)	3.0(7)
C26	19.6(10)	21.3(10)	49.3(12)	19.7(9)	8.0(9)	7.7(8)
C25	31.6(11)	19.3(10)	47.3(12)	16.5(9)	22.5(9)	11.6(9)
C15	36.9(12)	24.3(10)	26.7(10)	9.4(8)	15.2(9)	12.7(9)
C16	48.8(13)	25.0(10)	25.4(10)	7.3(8)	15.1(9)	11.1(10)
C24	46.3(13)	20.8(10)	18.2(9)	7.4(7)	11.4(8)	5.5(9)
C31	47.4(13)	21.1(10)	21.1(9)	5.4(8)	12.3(9)	6.2(9)
C13	35.1(12)	20.5(10)	34.3(11)	5.0(8)	9.1(9)	4.3(9)
C32	62.9(16)	18.4(10)	25.5(10)	2.7(8)	2.4(10)	-0.3(10)
C34	22.6(19)	16(2)	15(2)	4(2)	6(2)	-0.1(14)
C33	95(2)	24.9(11)	28.5(11)	5.3(9)	-4.7(12)	11.0(13)
C35	28(3)	24(3)	19(3)	8(2)	7(2)	3(2)
C36	29(3)	30(2)	23(2)	9.1(17)	7.9(17)	9(2)
C34A	22.6(19)	16(2)	15(2)	4(2)	6(2)	-0.1(14)
C35A	26(2)	21(2)	23(2)	9.5(18)	5.5(17)	5.8(17)
C36A	26(2)	34(3)	30(2)	14(2)	9.4(17)	13(2)

Table S33 Bond Lengths for [Mn(L9)Cl2].

Atom	Atom	Length/Å	Atom	Atom	Length/Å
Mn1	Cl1	2.4325(5)	N2	C2	1.469(2)
Mn1	Cl2	2.4444(5)	N5	C28	1.490(2)
Mn1	N4	2.2981(13)	N5	C21	1.500(2)
Mn1	N1	2.3378(13)	N5	C31	1.496(2)

Table S33 Bond Lengths for [Mn(L9)Cl2].

Atom	Atom	Length/Å	Atom	Atom	Length/Å
Mn1	N3	2.3242(14)	N7	C25	1.498(2)
Mn1	N2	2.2706(14)	N7	C24	1.485(2)
Mn2	Cl4	2.4061(5)	N7	C34	1.451(7)
Mn2	Cl3	2.4525(6)	N7	C34A	1.531(6)
Mn2	N8	2.2873(14)	C7	C8	1.523(2)
Mn2	N6	2.3138(14)	C6	C5	1.539(2)
Mn2	N5	2.3510(14)	C3	C4	1.520(2)
Mn2	N7	2.3786(15)	C11	C12	1.498(2)
N4	C7	1.470(2)	C23	C24	1.536(3)
N4	C6	1.470(2)	C30	C29	1.534(2)
N4	C10	1.476(2)	C14	C15	1.500(2)
N8	C30	1.474(2)	C10	C9	1.535(2)
N8	C27	1.471(2)	C27	C28	1.528(3)
N8	C26	1.468(2)	C2	C1	1.537(2)
N1	C11	1.485(2)	C22	C21	1.514(2)
N1	C8	1.492(2)	C12	C13	1.316(3)
N1	C1	1.491(2)	C26	C25	1.521(3)
N3	C5	1.491(2)	C15	C16	1.310(3)
N3	C14	1.492(2)	C31	C32	1.482(3)
N3	C4	1.494(2)	C32	C33	1.321(3)
N6	C23	1.465(2)	C34	C35	1.508(7)
N6	C29	1.479(2)	C35	C36	1.309(6)
N6	C22	1.468(2)	C34A	C35A	1.514(6)
N2	C3	1.470(2)	C35A	C36A	1.305(6)
N2	C9	1.480(2)			

Table S34 Bond Angles for [Mn(L9)Cl2].

Atom	Atom	Atom	Angle/˚	Atom	Atom	Atom	Angle/˚
Cl1	Mn1	Cl2	95.513(16)	C23	N6	C22	114.20(13)
N4	Mn1	Cl1	93.95(4)	C29	N6	Mn2	116.15(10)
N4	Mn1	Cl2	170.54(4)	C22	N6	Mn2	100.54(10)
N4	Mn1	N1	78.18(5)	C22	N6	C29	112.25(13)
N4	Mn1	N3	73.74(5)	C3	N2	Mn1	100.11(9)
N1	Mn1	Cl1	98.11(4)	C3	N2	C9	112.85(13)
N1	Mn1	Cl2	100.46(4)	C9	N2	Mn1	117.03(10)
N3	Mn1	Cl1	104.70(4)	C2	N2	Mn1	101.43(10)
N3	Mn1	Cl2	103.72(4)	C2	N2	C3	113.35(13)
N3	Mn1	N1	144.69(5)	C2	N2	C9	111.23(13)
N2	Mn1	Cl1	167.40(4)	C28	N5	Mn2	106.46(10)
N2	Mn1	Cl2	95.73(4)	C28	N5	C21	111.45(14)
N2	Mn1	N4	74.86(5)	C28	N5	C31	111.15(13)
N2	Mn1	N1	74.23(5)	C21	N5	Mn2	106.76(9)
N2	Mn1	N3	78.08(5)	C31	N5	Mn2	113.47(11)

Table S34 Bond Angles for [Mn(L9)Cl2].

Atom	Atom	Atom	Angle/˚	Atom	Atom	Atom	Angle/˚
Cl4	Mn2	Cl3	98.584(19)	C31	N5	C21	107.52(14)
N8	Mn2	Cl4	166.87(4)	C25	N7	Mn2	105.67(10)
N8	Mn2	Cl3	94.05(4)	C25	N7	C34A	109.8(3)
N8	Mn2	N6	74.15(5)	C24	N7	Mn2	107.65(10)
N8	Mn2	N5	74.50(5)	C24	N7	C25	113.27(15)
N8	Mn2	N7	77.58(5)	C24	N7	C34A	118.6(2)
N6	Mn2	Cl4	93.87(4)	C34	N7	Mn2	115.2(3)
N6	Mn2	Cl3	165.31(4)	C34	N7	C25	112.8(4)
N6	Mn2	N5	77.55(5)	C34	N7	C24	102.3(3)
N6	Mn2	N7	73.09(5)	C34A	N7	Mn2	100.2(2)
N5	Mn2	Cl4	97.99(4)	N4	C7	C8	111.47(14)
N5	Mn2	Cl3	108.19(4)	N4	C6	C5	111.52(14)
N5	Mn2	N7	143.88(5)	N2	C3	C4	110.23(14)
N7	Mn2	Cl4	104.46(4)	N1	C11	C12	116.24(13)
N7	Mn2	Cl3	96.13(4)	N3	C5	C6	113.04(13)
C7	N4	Mn1	99.38(9)	N6	C23	C24	110.99(14)
C7	N4	C10	112.95(13)	N8	C30	C29	112.98(13)
C6	N4	Mn1	101.32(9)	N3	C14	C15	115.33(14)
C6	N4	C7	114.06(14)	N6	C29	C30	113.61(14)
C6	N4	C10	112.00(13)	N4	C10	C9	113.67(14)
C10	N4	Mn1	116.10(10)	N3	C4	C3	112.83(14)
C30	N8	Mn2	117.56(10)	N2	C9	C10	113.21(14)
C27	N8	Mn2	100.05(10)	N1	C8	C7	113.35(13)
C27	N8	C30	111.15(13)	N8	C27	C28	112.00(14)
C26	N8	Mn2	100.31(10)	N2	C2	C1	111.04(14)
C26	N8	C30	112.51(14)	N6	C22	C21	110.78(14)
C26	N8	C27	114.45(14)	N5	C28	C27	114.21(14)
C11	N1	Mn1	106.06(9)	C13	C12	C11	123.91(17)
C11	N1	C8	111.85(13)	N5	C21	C22	113.50(14)
C11	N1	C1	111.04(13)	N1	C1	C2	113.87(13)
C8	N1	Mn1	106.67(9)	N8	C26	C25	111.18(15)
C1	N1	Mn1	107.22(9)	N7	C25	C26	113.99(15)
C1	N1	C8	113.51(13)	C16	C15	C14	123.32(18)
C5	N3	Mn1	108.69(10)	N7	C24	C23	114.10(14)
C5	N3	C14	112.23(13)	C32	C31	N5	113.75(17)
C5	N3	C4	112.61(13)	C33	C32	C31	123.5(2)
C14	N3	Mn1	105.19(10)	N7	C34	C35	117.6(5)
C14	N3	C4	110.88(13)	C36	C35	C34	123.0(5)
C4	N3	Mn1	106.79(9)	C35A	C34A	N7	115.8(4)
C23	N6	Mn2	101.34(10)	C36A	C35A	C34A	129.9(4)
C23	N6	C29	111.58(13)				

Table S35 Torsion Angles for [Mn(L9)Cl2].

A	B	C	D	Angle/˚	A	B	C	D	Angle/˚
Mn1	N4	C7	C8	59.84(14)	C11	N1	C8	C7	-101.50(16)
Mn1	N4	C6	C5	-57.60(15)	C11	N1	C1	C2	124.70(15)
Mn1	N4	C10	C9	-19.62(17)	C5	N3	C14	C15	64.6(2)
Mn1	N1	C11	C12	176.59(12)	C5	N3	C4	C3	134.52(14)
Mn1	N1	C8	C7	14.05(16)	C23	N6	C29	C30	136.15(15)
Mn1	N1	C1	C2	9.25(16)	C23	N6	C22	C21	-167.19(14)
Mn1	N3	C5	C6	9.94(17)	C30	N8	C27	C28	-66.16(18)
Mn1	N3	C14	C15	-177.40(13)	C30	N8	C26	C25	65.20(18)
Mn1	N3	C4	C3	15.29(15)	C14	N3	C5	C6	125.87(16)
Mn1	N2	C3	C4	60.64(14)	C14	N3	C4	C3	-98.79(16)
Mn1	N2	C9	C10	-20.35(17)	C29	N6	C23	C24	-64.72(18)
Mn1	N2	C2	C1	-58.23(14)	C29	N6	C22	C21	64.53(18)
Mn2	N8	C30	C29	20.97(17)	C10	N4	C7	C8	-63.82(18)
Mn2	N8	C27	C28	58.76(15)	C10	N4	C6	C5	66.79(17)
Mn2	N8	C26	C25	-60.56(15)	C4	N3	C5	C6	-108.17(16)
Mn2	N6	C23	C24	59.53(14)	C4	N3	C14	C15	-62.32(19)
Mn2	N6	C29	C30	20.69(18)	C9	N2	C3	C4	-64.54(17)
Mn2	N6	C22	C21	-59.56(14)	C9	N2	C2	C1	66.94(18)
Mn2	N5	C28	C27	-9.23(17)	C8	N1	C11	C12	-67.49(18)
Mn2	N5	C21	C22	-16.85(17)	C8	N1	C1	C2	-108.28(16)
Mn2	N5	C31	C32	-59.92(18)	C27	N8	C30	C29	135.35(15)
Mn2	N7	C25	C26	-13.10(18)	C27	N8	C26	C25	-166.67(15)
Mn2	N7	C24	C23	-7.69(17)	C2	N2	C3	C4	167.87(14)
Mn2	N7	C34	C35	-176.0(4)	C2	N2	C9	C10	-136.26(15)
Mn2	N7	C34A	C35A	179.8(4)	C22	N6	C23	C24	166.66(14)
N4	C7	C8	N1	-53.38(19)	C22	N6	C29	C30	-94.21(17)
N4	C6	C5	N3	33.0(2)	C28	N5	C21	C22	-132.71(15)
N4	C10	C9	N2	25.7(2)	C28	N5	C31	C32	60.0(2)
N8	C30	C29	N6	-26.7(2)	C21	N5	C28	C27	106.82(17)
N8	C27	C28	N5	-34.4(2)	C21	N5	C31	C32	-177.74(15)
N8	C26	C25	N7	52.6(2)	C1	N1	C11	C12	60.44(18)
N1	C11	C12	C13	-122.2(2)	C1	N1	C8	C7	131.90(15)
N3	C14	C15	C16	111.8(2)	C26	N8	C30	C29	-94.80(17)
N6	C23	C24	N7	-35.7(2)	C26	N8	C27	C28	165.03(15)
N6	C22	C21	N5	54.89(19)	C25	N7	C24	C23	108.75(16)
N2	C3	C4	N3	-54.13(18)	C25	N7	C34	C35	62.5(6)
N2	C2	C1	N1	33.46(19)	C25	N7	C34A	C35A	68.9(5)

Table S35 Torsion Angles for [Mn(L9)Cl2].

A	B	C	D	Angle/˚	A	B	C	D	Angle/˚
N5	C31	C32	C33	-124.9(2)	C24	N7	C25	C26	-130.70(17)
N7	C34	C35	C36	-147.1(5)	C24	N7	C34	C35	-59.5(6)
N7	C34A	C35A	C36A	2.3(8)	C24	N7	C34A	C35A	-63.6(6)
C7	N4	C6	C5	-163.35(14)	C31	N5	C28	C27	-133.26(16)
C7	N4	C10	C9	94.24(17)	C31	N5	C21	C22	105.24(16)
C6	N4	C7	C8	166.79(14)	C34	N7	C25	C26	113.6(3)
C6	N4	C10	C9	-135.32(15)	C34	N7	C24	C23	-129.5(3)
C3	N2	C9	C10	95.05(17)	C34A	N7	C25	C26	94.1(2)
C3	N2	C2	C1	-164.63(14)	C34A	N7	C24	C23	-120.3(3)

Table S36 Hydrogen Atom Coordinates (Å×10^4) and Isotropic Displacement Parameters (Å^2×10^3) for [Mn(L9)Cl2].

Atom	*x*	*y*	*z*	U(eq)
H7A	9423.64	8616.64	9496.39	24
H7B	8368.5	7939.58	9932.81	24
H6A	9089.32	6215.1	8816.83	26
H6B	10090.51	7170.78	8594.04	26
H3A	5256.8	7367.86	6584.33	23
H3B	3903.15	6574.2	6793.69	23
H11A	7832.63	10765.83	8620.88	22
H11B	9037.19	10543.6	9266.97	22
H5A	9650.69	5865.73	7412.45	26
H5B	7953.55	5341.66	7557.29	26
H23A	911.49	2897.29	5613.89	28
H23B	60.12	1697.42	5104.01	28
H30A	878.6	364.04	7194.23	24
H30B	1960.99	-223.12	6579.89	24
H14A	9792.71	7027.85	6652.44	26
H14B	8160.22	7100.44	6173.48	26
H29A	978.06	370.5	5631.65	25
H29B	-528.31	393.46	6095.26	25
H10A	6095.55	6746.12	9252.8	26
H10B	6272.5	5958.16	8405.08	26
H4A	5779.31	5610.99	7140.47	24
H4B	5980.27	5667.5	6274.89	24
H9A	4016.92	6444.44	8045.8	25
H9B	4380.54	7563.56	8749.99	25
H8A	6163.3	8709.15	9699.4	25
H8B	7645.54	9663.9	10105.05	25
H27A	4106.91	2061.87	8331.01	31

Table S36 Hydrogen Atom Coordinates (Å×10^4) and Isotropic Displacement Parameters (Å^2×10^3) for [Mn(L9)Cl2].

Atom	*x*	*y*	*z*	U(eq)
H27B	3126.7	889.72	8211.18	31
H2A	3390.63	8406.98	7842.66	24
H2B	4756.7	8917.84	7444.16	24
H22A	-1614.5	1912.9	6331.4	26
H22B	-508.96	3079.42	6666.28	26
H28A	814.46	1478.94	8376.47	31
H28B	2041.25	2487.86	8968.56	31
H12	7447.31	11389.88	10262.19	26
H21A	-1338.1	2594.03	7732.16	28
H21B	-700.85	1481.71	7443.38	28
H1A	5111.12	10181.14	8642.16	25
H1B	4599.78	9326.68	9080.83	25
H26A	4634.72	380.87	6910.07	33
H26B	5350.42	1642.08	7221.48	33
H25A	5384.95	1030.4	5865.04	35
H25B	3548.43	474.6	5700.98	35
H15	9335.02	5122.38	5983.72	33
H16A	7325.51	5745.69	4951.29	38
H16B	8013.8	4608.74	4753.2	38
H24A	2394.37	1109.36	4945.13	33
H24B	2860.19	2309.11	4921.71	33
H31A	437.23	4250.75	8247	36
H31B	68.51	3655.96	8882.05	36
H13A	7001.69	12423.45	9134.03	37
H13B	6737.63	12864.25	10066.41	37
H32	3254.6	4618.56	8832.19	45
H34A	6053.54	2958.29	6127.25	22
H34B	4754.56	3418.1	5685.26	22
H33A	1616.83	4616.44	10105.86	61
H33B	3479.45	5183.68	10158.22	61
H35	4948.07	1762.07	4503.54	28
H36A	7945.44	2958.48	5227.29	32
H36B	7481.01	2137.53	4312.81	32
H34C	5024.47	3614.77	6006.24	22
H34D	6059.26	3026.35	6477.8	22
H35A	7255.01	3118.17	5347.68	27
H36C	5112.69	1246.92	4577.56	33
H36D	6757.43	1706.26	4289.88	33

Table S37 Atomic Occupancy for [Mn(L9)Cl2].

Atom	*Occupancy*	Atom	*Occupancy*	Atom	*Occupancy*
C34	0.471(5)	H34A	0.471(5)	H34B	0.471(5)

Table S37 Atomic Occupancy for [Mn(L9)Cl2].

Atom	*Occupancy*	Atom	*Occupancy*	Atom	*Occupancy*
C35	0.471(5)	H35	0.471(5)	C36	0.471(5)
H36A	0.471(5)	H36B	0.471(5)	C34A	0.529(5)
H34C	0.529(5)	H34D	0.529(5)	C35A	0.529(5)
H35A	0.529(5)	C36A	0.529(5)	H36C	0.529(5)
H36D	0.529(5)				

Experimental

Single crystals of $C_{16}H_{30}Cl_2MnN_4$ **[[Mn(L9)Cl2]]** were **[]**. A suitable crystal was selected and **[]** on a **Bruker PHOTON100 CMOS** diffractometer. The crystal was kept at 120 K during data collection. Using Olex2 [1], the structure was solved with the SHELXT [2] structure solution program using Intrinsic Phasing and refined with the SHELXL [3] refinement package using Least Squares minimisation.

1. Dolomanov, O.V., Bourhis, L.J., Gildea, R.J, Howard, J.A.K. & Puschmann, H. (2009), J. Appl. Cryst. 42, 339-341.
2. Sheldrick, G.M. (2015). Acta Cryst. A71, 3-8.
3. Sheldrick, G.M. (2015). Acta Cryst. C71, 3-8.

Crystal structure determination of [[Mn(L9)Cl2]]

Crystal Data for $C_{16}H_{30}Cl_2MnN_4$ (M =404.28 g/mol): triclinic, space group P-1 (no. 2), a = 8.5540(11) Å, b = 13.2482(17) Å, c = 17.920(2) Å, α = 107.492(2)°, β = 95.600(2)°, γ = 97.994(2)°, V = 1897.0(4) Å^3, Z = 4, T = 120 K, μ(synchrotron) = 0.982 mm^{-1}, $Dcalc$ = 1.416 g/cm^3, 29491 reflections measured (2.41° ≤ 2Θ ≤ 56.584°), 9415 unique (R_{int} = 0.0370, R_{sigma} = 0.0433) which were used in all calculations. The final R_1 was 0.0307 (I > 2σ(I)) and wR_2 was 0.0645 (all data).

Refinement model description

Number of restraints - 41, number of constraints - unknown.

Details:

```
1. Fixed Uiso
 At 1.2 times of:
  All C(H) groups, All C(H,H) groups
2. Restrained distances
 N7-C34 ≈ N7-C34A
 with sigma of 0.02
 C34-C35 ≈ C34A-C35A
 with sigma of 0.02
 C35-C36 ≈ C35A-C36A
 with sigma of 0.02
 N7-C35 ≈ N7-C35A
 with sigma of 0.04
 C34-C36 ≈ C34A-C36A
 with sigma of 0.04
3. Uiso/Uaniso restraints and constraints
C35A ≈ C35: within 2A with sigma of 0.04 and sigma for terminal atoms of
0.08 within 2A
C36A ≈ C36: within 2A with sigma of 0.04 and sigma for terminal atoms of
0.08 within 2A
C34 ≈ C34A: within 2A with sigma of 0.04 and sigma for terminal atoms of
0.08 within 2A
Uanis(C34) = Uanis(C34A)
4. Rigid body (RIGU) restrains
 N7, C34A, C34, C35A, C35
 with sigma for 1-2 distances of 0.004 and sigma for 1-3 distances of 0.004
5. Others
 Sof(C34A)=Sof(H34C)=Sof(H34D)=Sof(C35A)=Sof(H35A)=Sof(C36A)=Sof(H36C)=
 Sof(H36D)=1-FVAR(1)
 Sof(C34)=Sof(H34A)=Sof(H34B)=Sof(C35)=Sof(H35)=Sof(C36)=Sof(H36A)=Sof(H36B)=
 FVAR(1)
6.a Secondary CH2 refined with riding coordinates:
 C7(H7A,H7B), C6(H6A,H6B), C3(H3A,H3B), C11(H11A,H11B), C5(H5A,H5B), C23(H23A,
 H23B), C30(H30A,H30B), C14(H14A,H14B), C29(H29A,H29B), C10(H10A,H10B), C4(H4A,
 H4B), C9(H9A,H9B), C8(H8A,H8B), C27(H27A,H27B), C2(H2A,H2B), C22(H22A,H22B),
 C28(H28A,H28B), C21(H21A,H21B), C1(H1A,H1B), C26(H26A,H26B), C25(H25A,H25B),
 C24(H24A,H24B), C31(H31A,H31B), C34(H34A,H34B), C34A(H34C,H34D)
6.b Aromatic/amide H refined with riding coordinates:
 C12(H12), C15(H15), C32(H32), C35(H35), C35A(H35A)
```

```
6.c X=CH2 refined with riding coordinates:
 C16(H16A,H16B), C13(H13A,H13B), C33(H33A,H33B), C36(H36A,H36B), C36A(H36C,H36D)
```

This report has been created with Olex2, compiled on 2020.11.12 svn.r5f609507 for OlexSys. Please let us know if there are any errors or if you would like to have additional features.

[Mn(L11)Cl_2]

Table S38 Crystal data and structure refinement for [Mn(L11)Cl2].

Identification code	[Mn(L11)Cl2]
Empirical formula	$C_{20}H_{32}Cl_2MnN_4$
Formula weight	454.33
Temperature/K	120
Crystal system	monoclinic
Space group	$P2_1/c$
a/Å	8.4238(4)
b/Å	13.5803(6)
c/Å	18.6065(8)
α/°	90
β/°	98.507(2)
γ/°	90
Volume/Å^3	2105.12(16)
Z	4
ρ_{calc}g/cm^3	1.434
μ/mm^{-1}	0.894
F(000)	956.0
Crystal size/mm^3	0.253 × 0.169 × 0.078
Radiation	MoKα (λ = 0.71073)
2Θ range for data collection/°	3.728 to 56.632
Index ranges	-11 ≤ h ≤ 11, -18 ≤ k ≤ 18, -24 ≤ l ≤ 24
Reflections collected	49812
Independent reflections	5246 [R_{int} = 0.0340, R_{sigma} = 0.0172]
Data/restraints/parameters	5246/0/244
Goodness-of-fit on F^2	1.089
Final R indexes [I>=2σ (I)]	R_1 = 0.0264, wR_2 = 0.0603
Final R indexes [all data]	R_1 = 0.0294, wR_2 = 0.0615
Largest diff. peak/hole / e Å^{-3}	0.39/-0.22

Table S39 Fractional Atomic Coordinates (×10^4) and Equivalent Isotropic Displacement Parameters (Å^2×10^3) for [Mn(L11)Cl2]. U_{eq} is defined as 1/3 of of the trace of the orthogonalised U_{IJ} tensor.

Atom	*x*	*y*	*z*	U(eq)
Mn1	7108.9(2)	6394.4(2)	2445.8(2)	10.13(5)
Cl1	8299.3(4)	4838.3(2)	2883.6(2)	18.20(7)
Cl2	4613.5(4)	5716.2(2)	1763.8(2)	16.51(7)
N4	6614.9(12)	7986.9(8)	2086.8(6)	12.7(2)
N1	5879.5(12)	7055.5(8)	3426.7(6)	12.7(2)

Table S39 Fractional Atomic Coordinates (×10^4) and Equivalent Isotropic Displacement Parameters (Å^2×10^3) for [Mn(L11)Cl2]. U_{eq} is defined as 1/3 of of the trace of the orthogonalised U_{IJ} tensor.

Atom	*x*	*y*	*z*	U(eq)
N2	9036.8(13)	7331.2(8)	3147.0(6)	13.5(2)
N3	8947.1(13)	6761.0(8)	1617.2(6)	13.9(2)
C6	6535.5(16)	7865.7(10)	1294.3(7)	15.7(2)
C12	3848.6(15)	6467.6(10)	4248.9(7)	15.1(2)
C11	4682.6(15)	6316.8(9)	3582.1(7)	14.3(2)
C10	7787.3(16)	8750.1(9)	2383.2(7)	15.8(2)
C7	5028.6(15)	8136.8(10)	2315.9(7)	15.2(2)
C2	8881.0(15)	6989.8(11)	3884.0(7)	17.1(3)
C9	8865.3(16)	8414.0(10)	3083.3(7)	16.3(3)
C5	8144.4(16)	7509.3(10)	1095.9(7)	17.3(3)
C8	5113.4(15)	8006.5(9)	3136.5(7)	15.5(2)
C4	10423.8(15)	7170.8(10)	2072.0(7)	16.9(3)
C13	3741.1(16)	7358.6(11)	4610.7(7)	19.1(3)
C18	9427.2(17)	5879.7(10)	1218.1(8)	19.3(3)
C3	10499.8(15)	6973.4(10)	2885.6(7)	16.9(3)
C14	2935.5(17)	7416.1(12)	5215.0(8)	22.3(3)
C1	7176.2(16)	7186.7(10)	4060.6(7)	16.5(3)
C15	2251.3(17)	6588.5(12)	5468.5(8)	23.7(3)
C17	3117.8(19)	5645.3(11)	4504.6(9)	26.6(3)
C16	2331(2)	5700.2(12)	5110.6(9)	31.5(4)
C19	8016(2)	5347.4(11)	812.0(9)	26.9(3)
C20	7787(2)	5221.1(13)	97.0(10)	36.1(4)

Table S40 Anisotropic Displacement Parameters (Å^2×10^3) for [Mn(L11)Cl2]. The Anisotropic displacement factor exponent takes the form: $-2\pi^2[h^2a^{*2}U_{11}+2hka^*b^*U_{12}+…]$.

Atom	U_{11}	U_{22}	U_{33}	U_{23}	U_{13}	U_{12}
Mn1	9.93(9)	8.33(9)	12.41(9)	0.82(7)	2.52(6)	-0.02(6)
Cl1	16.20(15)	11.66(14)	27.72(17)	5.75(12)	6.54(12)	2.90(11)
Cl2	16.66(14)	14.88(14)	17.39(15)	-0.23(11)	0.55(11)	-4.73(11)
N4	11.4(5)	11.4(5)	15.6(5)	1.1(4)	3.0(4)	-0.4(4)
N1	11.1(5)	12.6(5)	14.7(5)	-0.5(4)	2.9(4)	-0.9(4)
N2	10.9(5)	14.1(5)	15.4(5)	0.2(4)	2.3(4)	-0.3(4)
N3	16.2(5)	10.7(5)	15.5(5)	1.0(4)	5.1(4)	1.5(4)
C6	18.1(6)	13.4(6)	15.6(6)	5.1(5)	2.1(5)	1.1(5)
C12	13.1(6)	18.3(6)	14.4(6)	1.1(5)	4.1(5)	2.0(5)
C11	14.4(6)	13.6(6)	16.0(6)	-1.3(5)	5.5(5)	-1.6(5)
C10	15.8(6)	8.9(5)	23.3(6)	-0.4(5)	5.0(5)	-1.9(5)
C7	10.9(5)	13.3(6)	21.9(6)	3.3(5)	3.8(5)	3.4(4)
C2	12.8(6)	23.2(7)	14.6(6)	0.6(5)	-0.2(5)	-1.0(5)
C9	14.4(6)	13.7(6)	21.1(6)	-3.5(5)	3.6(5)	-4.3(5)
C5	22.1(6)	15.1(6)	16.1(6)	4.2(5)	7.2(5)	1.1(5)
C8	13.5(6)	11.7(6)	22.1(6)	-1.0(5)	5.6(5)	1.8(5)
C4	12.3(6)	17.1(6)	22.7(7)	0.5(5)	7.1(5)	-1.3(5)

Table S40 Anisotropic Displacement Parameters (Å^2×10^3) for [Mn(L11)Cl2]. The Anisotropic displacement factor exponent takes the form: $-2\pi^2[h^2a^{*2}U_{11}+2hka^*b^*U_{12}+\ldots]$.

Atom	U_{11}	U_{22}	U_{33}	U_{23}	U_{13}	U_{12}
C13	18.3(6)	21.0(7)	19.0(6)	-3.3(5)	5.8(5)	-2.6(5)
C18	24.3(7)	15.0(6)	21.2(7)	-2.1(5)	11.7(5)	2.9(5)
C3	9.5(5)	19.0(6)	22.1(6)	1.3(5)	2.3(5)	-0.1(5)
C14	18.2(6)	30.1(8)	18.6(6)	-6.6(6)	3.0(5)	2.8(6)
C1	16.2(6)	20.6(6)	12.6(6)	-2.4(5)	1.9(5)	-2.5(5)
C15	18.8(7)	36.9(8)	17.1(6)	4.9(6)	7.9(5)	7.9(6)
C17	33.7(8)	17.2(7)	33.0(8)	2.6(6)	18.9(7)	2.8(6)
C16	38.0(9)	25.5(8)	36.0(9)	12.3(7)	22.4(7)	4.4(7)
C19	32.4(8)	19.9(7)	30.8(8)	-8.4(6)	12.2(6)	-2.1(6)
C20	45.7(10)	27.6(9)	34.1(9)	-10.7(7)	3.6(8)	5.3(7)

Table S41 Bond Lengths for [Mn(L11)Cl2].

Atom	Atom	Length/Å	Atom	Atom	Length/Å
Mn1	Cl1	2.4276(4)	N3	C4	1.5038(17)
Mn1	Cl2	2.4674(4)	N3	C18	1.4954(16)
Mn1	N4	2.2834(11)	C6	C5	1.5347(18)
Mn1	N1	2.4019(11)	C12	C11	1.5273(17)
Mn1	N2	2.3090(11)	C12	C13	1.3941(19)
Mn1	N3	2.3935(11)	C12	C17	1.3924(19)
N4	C6	1.4754(16)	C10	C9	1.5427(19)
N4	C10	1.4807(16)	C7	C8	1.5283(18)
N4	C7	1.4755(16)	C2	C1	1.5432(18)
N1	C11	1.4808(16)	C4	C3	1.5295(19)
N1	C8	1.5076(16)	C13	C14	1.3988(19)
N1	C1	1.4950(16)	C18	C19	1.497(2)
N2	C2	1.4717(17)	C14	C15	1.378(2)
N2	C9	1.4806(17)	C15	C16	1.384(2)
N2	C3	1.4726(16)	C17	C16	1.392(2)
N3	C5	1.4956(16)	C19	C20	1.327(2)

Table S42 Bond Angles for [Mn(L11)Cl2].

Atom	Atom	Atom	Angle/˚	Atom	Atom	Atom	Angle/˚
Cl1	Mn1	Cl2	97.230(13)	C9	N2	Mn1	116.74(8)
N4	Mn1	Cl1	166.16(3)	C3	N2	Mn1	100.64(8)
N4	Mn1	Cl2	95.66(3)	C3	N2	C9	112.20(10)
N4	Mn1	N1	77.75(4)	C5	N3	Mn1	106.59(7)
N4	Mn1	N2	74.09(4)	C5	N3	C4	111.75(10)
N4	Mn1	N3	73.85(4)	C4	N3	Mn1	105.94(7)
N1	Mn1	Cl1	105.87(3)	C18	N3	Mn1	113.68(8)
N1	Mn1	Cl2	95.74(3)	C18	N3	C5	110.56(10)
N2	Mn1	Cl1	93.95(3)	C18	N3	C4	108.28(10)
N2	Mn1	Cl2	166.11(3)	N4	C6	C5	111.38(10)

Table S42 Bond Angles for [Mn(L11)Cl2].

Atom	Atom	Atom	Angle/˚	Atom	Atom	Atom	Angle/˚
N2	Mn1	N1	73.20(4)	C13	C12	C11	125.39(12)
N2	Mn1	N3	77.37(4)	C17	C12	C11	116.73(12)
N3	Mn1	Cl1	97.05(3)	C17	C12	C13	117.87(12)
N3	Mn1	Cl2	109.24(3)	N1	C11	C12	118.43(11)
N3	Mn1	N1	143.56(4)	N4	C10	C9	112.60(10)
C6	N4	Mn1	99.56(7)	N4	C7	C8	111.23(10)
C6	N4	C10	112.29(10)	N2	C2	C1	110.97(10)
C10	N4	Mn1	118.12(8)	N2	C9	C10	113.55(10)
C7	N4	Mn1	100.36(7)	N3	C5	C6	112.89(10)
C7	N4	C6	113.26(10)	N1	C8	C7	114.29(10)
C7	N4	C10	112.24(10)	N3	C4	C3	113.71(10)
C11	N1	Mn1	106.12(7)	C12	C13	C14	120.69(13)
C11	N1	C8	112.49(10)	N3	C18	C19	112.59(11)
C11	N1	C1	111.46(10)	N2	C3	C4	110.76(10)
C8	N1	Mn1	104.95(7)	C15	C14	C13	120.46(14)
C1	N1	Mn1	107.29(7)	N1	C1	C2	113.93(10)
C1	N1	C8	113.87(10)	C14	C15	C16	119.58(13)
C2	N2	Mn1	101.58(8)	C16	C17	C12	121.43(14)
C2	N2	C9	111.49(10)	C15	C16	C17	119.94(14)
C2	N2	C3	113.46(10)	C20	C19	C18	123.73(16)

Table S43 Torsion Angles for [Mn(L11)Cl2].

A	B	C	D	Angle/˚	A	B	C	D	Angle/˚
Mn1	N4	C6	C5	-62.91(10)	C10	N4	C6	C5	62.94(13)
Mn1	N4	C10	C9	-19.55(13)	C10	N4	C7	C8	-65.48(13)
Mn1	N4	C7	C8	60.85(11)	C7	N4	C6	C5	-168.63(10)
Mn1	N1	C11	C12	172.22(9)	C7	N4	C10	C9	96.49(12)
Mn1	N1	C8	C7	12.34(12)	C2	N2	C9	C10	-135.77(11)
Mn1	N1	C1	C2	6.78(13)	C2	N2	C3	C4	168.30(11)
Mn1	N2	C2	C1	-60.18(11)	C9	N2	C2	C1	64.86(14)
Mn1	N2	C9	C10	-19.68(13)	C9	N2	C3	C4	-64.23(14)
Mn1	N2	C3	C4	60.60(11)	C5	N3	C4	C3	131.26(11)
Mn1	N3	C5	C6	5.49(12)	C5	N3	C18	C19	-63.67(15)
Mn1	N3	C4	C3	15.55(12)	C8	N1	C11	C12	-73.56(14)
Mn1	N3	C18	C19	56.16(13)	C8	N1	C1	C2	-108.88(12)
N4	C6	C5	N3	39.33(15)	C4	N3	C5	C6	-109.82(12)
N4	C10	C9	N2	24.98(15)	C4	N3	C18	C19	173.59(12)
N4	C7	C8	N1	-52.12(14)	C13	C12	C11	N1	19.76(19)
N2	C2	C1	N1	36.35(15)	C13	C12	C17	C16	-1.3(2)
N3	C4	C3	N2	-54.46(14)	C13	C14	C15	C16	-1.6(2)

Table S43 Torsion Angles for [Mn(L11)Cl2].

A	B	C	D	Angle/˚	A	B	C	D	Angle/˚
N3	C18	C19	C20	119.51(16)	C18	N3	C5	C6	129.50(12)
C6	N4	C10	C9	−134.55(11)	C18	N3	C4	C3	−106.74(12)
C6	N4	C7	C8	166.06(10)	C3	N2	C2	C1	−167.29(11)
C12	C13	C14	C15	0.8(2)	C3	N2	C9	C10	95.72(13)
C12	C17	C16	C15	0.6(3)	C14	C15	C16	C17	0.9(2)
C11	N1	C8	C7	−102.60(12)	C1	N1	C11	C12	55.73(15)
C11	N1	C1	C2	122.56(12)	C1	N1	C8	C7	129.37(11)
C11	C12	C13	C14	179.50(13)	C17	C12	C11	N1	−161.32(13)
C11	C12	C17	C16	179.71(15)	C17	C12	C13	C14	0.6(2)

Table S44 Hydrogen Atom Coordinates (Å×10[4]) and Isotropic Displacement Parameters (Å[2]×10[3]) for [Mn(L11)Cl2].

Atom	*x*	*y*	*z*	U(eq)
H6A	6247.1	8502.86	1050.99	19
H6B	5686.04	7383.64	1116.51	19
H11A	5223.27	5667.91	3631.73	17
H11B	3840.51	6278.93	3151.29	17
H10A	7200.53	9352.46	2487.95	19
H10B	8471.81	8918.21	2011.77	19
H7A	4254.52	7658.45	2061.39	18
H7B	4637.97	8807.76	2176.99	18
H2A	9676.05	7335.14	4241.92	21
H2B	9111.3	6275.32	3922.61	21
H9A	9943.01	8710.12	3096.05	20
H9B	8408.83	8665.21	3508.51	20
H5A	7960.18	7219.75	602.22	21
H5B	8868.32	8081.62	1085.13	21
H8A	5727.77	8562.74	3384.02	19
H8B	4011.12	8038.09	3260.13	19
H4A	10459.47	7890.85	1992.82	20
H4B	11384.03	6879.7	1905.84	20
H13	4220.09	7932.78	4445.05	23
H18A	10038.36	5421.87	1569.77	23
H18B	10144.06	6091.88	871.03	23
H3A	10617.9	6257.47	2978.71	20
H3B	11449.24	7308.61	3155.41	20
H14	2859.56	8030.1	5452.21	27
H1A	6969.1	6735.46	4454.24	20
H1B	7129.02	7868.67	4244.21	20
H15	1728.32	6627.21	5886.33	28

Table S44 Hydrogen Atom Coordinates (Å×10[4]) and Isotropic Displacement Parameters (Å[2]×10[3]) for [Mn(L11)Cl2].

Atom	*x*	*y*	*z*	U(eq)
H17	3157.28	5033.6	4260.18	32
H16	1848.47	5128.58	5278.33	38
H19	7239.87	5085.14	1081.58	32
H20A	8542.56	5475.27	-186.62	43
H20B	6866.88	4876.41	-131.3	43

Experimental

Single crystals of $C_{20}H_{32}Cl_2MnN_4$ **[[Mn(L11)Cl2]]** were []. A suitable crystal was selected and [] on a **Bruker PHOTON-II** diffractometer. The crystal was kept at 120 K during data collection. Using Olex2 [1], the structure was solved with the SHELXT [2] structure solution program using Intrinsic Phasing and refined with the SHELXL [3] refinement package using Least Squares minimisation.

1. Dolomanov, O.V., Bourhis, L.J., Gildea, R.J, Howard, J.A.K. & Puschmann, H. (2009), J. Appl. Cryst. 42, 339-341.
2. Sheldrick, G.M. (2015). Acta Cryst. A71, 3-8.
3. Sheldrick, G.M. (2015). Acta Cryst. C71, 3-8.

Crystal structure determination of [[Mn(L11)Cl2]]

Crystal Data for $C_{20}H_{32}Cl_2MnN_4$ (M =454.33 g/mol): monoclinic, space group P2$_1$/c (no. 14), a = 8.4238(4) Å, b = 13.5803(6) Å, c = 18.6065(8) Å, β = 98.507(2)°, V = 2105.12(16) Å^3, Z = 4, T = 120 K, μ(MoKα) = 0.894 mm^{-1}, $Dcalc$ = 1.434 g/cm^3, 49812 reflections measured (3.728° ≤ 2Θ ≤ 56.632°), 5246 unique (R_{int} = 0.0340, R_{sigma} = 0.0172) which were used in all calculations. The final R_1 was 0.0264 (I > 2σ(I)) and wR_2 was 0.0615 (all data).

Refinement model description

Number of restraints - 0, number of constraints - unknown.

Details:

```
1. Fixed Uiso
 At 1.2 times of:
  All C(H) groups, All C(H,H) groups
2.a Secondary CH2 refined with riding coordinates:
 C6(H6A,H6B), C11(H11A,H11B), C10(H10A,H10B), C7(H7A,H7B), C2(H2A,H2B), C9(H9A,
 H9B), C5(H5A,H5B), C8(H8A,H8B), C4(H4A,H4B), C18(H18A,H18B), C3(H3A,H3B),
 C1(H1A,H1B)
2.b Aromatic/amide H refined with riding coordinates:
 C13(H13), C14(H14), C15(H15), C17(H17), C16(H16), C19(H19)
2.c X=CH2 refined with riding coordinates:
 C20(H20A,H20B)
```

This report has been created with Olex2, compiled on 2020.11.12 svn.r5f609507 for OlexSys. Please let us know if there are any errors or if you would like to have additional features.

[Fe(L11)Cl$_2$].2MeCN

Table S45 Crystal data and structure refinement for [Fe(L11)Cl2].2MeCN.

Identification code	[Fe(L11)Cl2].2MeCN
Empirical formula	$C_{22}H_{35}Cl_2FeN_5$
Formula weight	496.30
Temperature/K	120.01
Crystal system	orthorhombic
Space group	Pnma
a/Å	14.4316(7)
b/Å	8.9132(5)
c/Å	19.5405(10)
α/°	90
β/°	90

γ/°	90
Volume/Å^3	2513.5(2)
Z	4
ρ_{calc}g/cm^3	1.311
μ/mm^{-1}	0.831
F(000)	1048.0
Crystal size/mm^3	0.216 × 0.169 × 0.088
Radiation	MoKα (λ = 0.71073)
2Θ range for data collection/°	3.508 to 56.726
Index ranges	-19 ≤ h ≤ 19, -11 ≤ k ≤ 11, -26 ≤ l ≤ 26
Reflections collected	46814
Independent reflections	3344 [R_{int} = 0.0444, R_{sigma} = 0.0174]
Data/restraints/parameters	3344/235/201
Goodness-of-fit on F^2	1.031
Final R indexes [I>=2σ (I)]	R_1 = 0.0284, wR_2 = 0.0652
Final R indexes [all data]	R_1 = 0.0372, wR_2 = 0.0693
Largest diff. peak/hole / e Å^{-3}	0.30/-0.23

Table S46 Fractional Atomic Coordinates (×10^4) and Equivalent Isotropic Displacement Parameters (Å^2×10^3) for [Fe(L11)Cl2].2MeCN. U_{eq} is defined as 1/3 of of the trace of the orthogonalised U_{IJ} tensor.

Atom	*x*	*y*	*z*	U(eq)
Fe1	4188.6(2)	2500	2918.3(2)	21.00(8)
Cl1	3138.8(2)	4465.7(4)	2608.2(2)	31.39(9)
N1	4006.5(10)	2500	4072.4(7)	20.4(3)
N2	5249.3(19)	4253(3)	3243.7(13)	20.3(7)
N3	5138.3(15)	2717(9)	2002.5(10)	28.8(14)
N4	5342.8(19)	1137(3)	3262.4(14)	20.7(7)
C1	4454.9(11)	3882.0(19)	4340.3(7)	35.3(4)
C2	4828.3(11)	4922.1(18)	3789.3(8)	32.3(3)
C3	5347(3)	5192(5)	2632(2)	32.1(9)
C4	5524(2)	4273(4)	1978.2(15)	34.3(7)
C5	5871(2)	1570(4)	2095.1(15)	33.2(7)
C6	5597(3)	375(5)	2617(2)	32.3(9)
C7	6152(4)	3568(7)	3437(3)	31.3(11)
C8	6132(4)	1898(7)	3591(3)	31.0(11)
C9	2984.7(13)	2500	4185.6(9)	26.8(4)
C10	2642.3(12)	2500	4915.7(9)	20.1(4)
C11	2463.9(11)	3822.4(18)	5261.6(8)	38.0(4)
C12	2138.3(12)	3819(2)	5930.4(9)	46.0(4)
C13	1984.8(16)	2500	6264.0(11)	39.7(5)
C14	4567.2(18)	2722(9)	1385.8(11)	26.7(16)
C15	5067(2)	2590(40)	711.1(13)	42(2)
C16	4910(4)	1654(7)	230(2)	97(2)

Table S46 Fractional Atomic Coordinates (×10^4^) and Equivalent Isotropic Displacement Parameters (Å²×10³) for [Fe(L11)Cl2].2MeCN. U_{eq} is defined as 1/3 of of the trace of the orthogonalised U_{IJ} tensor.

Atom	*x*	*y*	*z*	U(eq)
N5	3303.8(16)	7500	4620.4(11)	44.5(5)
C17	2785.2(17)	7500	4183.5(13)	35.7(5)
C18	2128(2)	7500	3615.8(15)	49.8(6)

Table S47 Anisotropic Displacement Parameters (Å²×10³) for [Fe(L11)Cl2].2MeCN. The Anisotropic displacement factor exponent takes the form: $-2\pi^2[h^2a^{*2}U_{11}+2hka^*b^*U_{12}+\ldots]$.

Atom	U_{11}	U_{22}	U_{33}	U_{23}	U_{13}	U_{12}
Fe1	17.71(13)	34.11(15)	11.19(12)	0	-0.95(9)	0
Cl1	29.77(17)	38.17(19)	26.23(17)	9.55(14)	-2.46(13)	3.06(14)
N1	20.5(7)	27.3(8)	13.5(7)	0	-0.3(6)	0
N3	23.0(9)	48(4)	15.5(8)	-0.1(12)	-0.2(7)	0.1(13)
C1	41.4(8)	45.9(9)	18.7(6)	-9.9(6)	6.5(6)	-18.5(7)
C2	36.5(8)	33.4(8)	26.9(7)	-4.5(6)	-3.5(6)	-8.6(7)
C3	35(2)	38(2)	22.9(16)	8.3(17)	2.1(15)	-10.6(17)
C4	32.5(16)	49.0(19)	21.6(14)	7.5(13)	3.3(12)	-7.8(15)
C5	28.4(15)	51(2)	19.9(13)	-3.1(14)	6.3(12)	5.3(14)
C6	28(2)	41(2)	28.1(17)	-8.1(18)	3.5(15)	8.1(16)
C7	22.1(16)	50(3)	22(2)	-2.1(18)	-3.2(16)	-6(2)
C8	19.3(15)	51(3)	23(2)	-1.6(16)	-4.1(16)	2.8(19)
C9	20.8(9)	46.5(12)	13.0(8)	0	-0.6(7)	0
C10	19.1(8)	26.5(9)	14.7(8)	0	0.4(6)	0
C11	46.6(9)	25.5(7)	42.0(9)	-0.8(6)	23.3(7)	-1.3(7)
C12	49.5(10)	45.8(10)	42.5(9)	-23.3(8)	22.2(8)	-12.8(8)
C13	32.8(11)	67.8(16)	18.7(9)	0	5.5(9)	0
C14	28.2(11)	36(5)	15.9(9)	1.6(12)	0.0(8)	0.5(14)
C15	36.0(12)	73(7)	17.3(10)	-3(4)	2.1(9)	-2(5)
C16	114(4)	142(5)	34(2)	-30(3)	31(2)	-53(4)
N5	55.6(13)	36.7(11)	41.1(11)	0	-4.8(10)	0
C17	43.2(13)	21.8(10)	42.0(13)	0	4.6(11)	0
C18	52.6(15)	44.5(14)	52.5(16)	0	-10.3(13)	0

Table S48 Bond Lengths for [Fe(L11)Cl2].2MeCN.

Atom	Atom	Length/Å	Atom	Atom	Length/Å
Fe1	Cl1	2.3942(4)	N4	C6	1.479(5)
Fe1	Cl1[1]	2.3942(4)	N4	C8	1.473(6)
Fe1	N1	2.2705(15)	C1	C2	1.520(2)
Fe1	N2	2.278(3)	C3	C4	1.538(5)
Fe1	N3	2.262(2)	C5	C6	1.527(5)
Fe1	N4	2.169(3)	C7	C8	1.519(6)
Fe1	N4[1]	2.169(3)	C9	C10	1.510(2)
N1	C1	1.4867(17)	C10	C11	1.3829(18)

Table S48 Bond Lengths for [Fe(L11)Cl2].2MeCN.

Atom	Atom	Length/Å	Atom	Atom	Length/Å
N1	C1[1]	1.4866(17)	C10	C11[1]	1.3830(18)
N1	C9	1.491(2)	C11	C12	1.389(2)
N2	C2	1.364(3)	C12	C13	1.362(2)
N2	C3	1.467(5)	C14	C15	1.507(4)
N2	C7	1.487(7)	C15	C16	1.28(2)
N3	C4	1.495(8)	N5	C17	1.135(3)
N3	C5	1.482(7)	C17	C18	1.460(4)
N3	C14	1.460(3)			

[1]+X,1/2-Y,+Z

Table S49 Bond Angles for [Fe(L11)Cl2].2MeCN.

Atom	Atom	Atom	Angle/˚	Atom	Atom	Atom	Angle/˚
Cl1	Fe1	Cl1[1]	94.08(2)	C3	N2	C7	110.9(3)
N1	Fe1	Cl1	100.26(3)	C7	N2	Fe1	112.2(3)
N1	Fe1	Cl1[1]	100.26(3)	C4	N3	Fe1	109.3(3)
N1	Fe1	N2	78.49(7)	C5	N3	Fe1	106.1(3)
N2	Fe1	Cl1[1]	176.23(8)	C5	N3	C4	112.2(3)
N2	Fe1	Cl1	89.65(8)	C14	N3	Fe1	108.13(15)
N3	Fe1	Cl1	96.93(16)	C14	N3	C4	100.4(5)
N3	Fe1	Cl1[1]	104.22(16)	C14	N3	C5	120.4(5)
N3	Fe1	N1	148.85(7)	C6	N4	Fe1	100.6(2)
N3	Fe1	N2	75.82(16)	C8	N4	Fe1	118.1(3)
N4[1]	Fe1	Cl1[1]	167.03(8)	C8	N4	C6	113.1(3)
N4	Fe1	Cl1	167.03(8)	N1	C1	C2	114.22(12)
N4[1]	Fe1	Cl1	98.89(8)	N2	C2	N4[1]	11.91(16)
N4	Fe1	Cl1[1]	98.89(8)	N2	C2	C1	116.41(18)
N4[1]	Fe1	N1	77.34(8)	C1	C2	N4[1]	105.29(15)
N4	Fe1	N1	77.34(8)	N2	C3	C4	112.9(3)
N4	Fe1	N2	77.38(12)	N3	C4	C3	114.0(3)
N4[1]	Fe1	N2	9.24(10)	N3	C5	C6	112.2(3)
N4[1]	Fe1	N3	74.46(14)	N4	C6	C5	108.3(3)
N4	Fe1	N3	80.08(15)	N2	C7	C8	115.8(6)
N4[1]	Fe1	N4	68.15(16)	N4	C8	C7	112.4(6)
C1[1]	N1	Fe1	107.42(8)	N1	C9	C10	117.64(15)
C1	N1	Fe1	107.42(8)	C11	C10	C9	121.52(9)
C1[1]	N1	C1	111.91(17)	C11[1]	C10	C9	121.52(9)
C1	N1	C9	112.22(10)	C11	C10	C11[1]	116.93(18)
C1[1]	N1	C9	112.22(10)	C10	C11	C12	121.41(15)
C9	N1	Fe1	105.18(10)	C13	C12	C11	120.47(16)
C2	N2	Fe1	102.64(16)	C12	C13	C12[1]	119.3(2)
C2	N2	C3	115.5(3)	N3	C14	C15	116.9(2)
C2	N2	C7	111.7(3)	C16	C15	C14	127.6(18)
C3	N2	Fe1	103.2(2)	N5	C17	C18	179.3(3)

[1]+X,1/2-Y,+Z

Table S50 Torsion Angles for [Fe(L11)Cl2].2MeCN.

A	B	C	D	Angle/˚	A	B	C	D	Angle/˚
Fe1	N1	C1	C2	4.26(16)	C2	N2	C3	C4	-161.4(3)
Fe1	N1	C9	C10	180.000(0)	C2	N2	C7	C8	97.2(6)
Fe1	N2	C2	N4[1]	72.3(9)	C3	N2	C2	N4[1]	-176.2(11)
Fe1	N2	C2	C1	50.5(2)	C3	N2	C2	C1	162.0(3)
Fe1	N2	C3	C4	-50.2(3)	C3	N2	C7	C8	-132.4(5)
Fe1	N2	C7	C8	-17.5(6)	C4	N3	C5	C6	136.5(3)
Fe1	N3	C4	C3	11.5(4)	C4	N3	C14	C15	75.8(17)
Fe1	N3	C5	C6	17.3(4)	C5	N3	C4	C3	-105.9(3)
Fe1	N3	C14	C15	-169.8(16)	C5	N3	C14	C15	-47.8(17)
Fe1	N4	C6	C5	60.7(3)	C6	N4	C8	C7	98.7(5)
Fe1	N4	C8	C7	-18.3(7)	C7	N2	C2	N4[1]	-48.2(9)
N1	C1	C2	N2	-39.9(2)	C7	N2	C2	C1	-70.0(3)
N1	C1	C2	N4[1]	-44.49(19)	C7	N2	C3	C4	70.2(5)
N1	C9	C10	C11	91.07(16)	C8	N4	C6	C5	-66.1(5)
N1	C9	C10	C11[1]	-91.07(16)	C9	N1	C1	C2	-110.87(16)
N2	C3	C4	N3	27.2(5)	C9	C10	C11	C12	179.22(17)
N2	C7	C8	N4	23.3(8)	C10	C11	C12	C13	-0.1(3)
N3	C5	C6	N4	-54.7(4)	C11[1]	C10	C11	C12	1.3(3)
N3	C14	C15	C16	127.4(14)	C11	C12	C13	C12[1]	-1.2(4)
C1[1]	N1	C1	C2	121.94(14)	C14	N3	C4	C3	125.0(3)
C1[1]	N1	C9	C10	63.51(10)	C14	N3	C5	C6	-105.7(4)
C1	N1	C9	C10	-63.51(10)					

[1]+X,1/2-Y,+Z

Table S51 Hydrogen Atom Coordinates (Å×10^4) and Isotropic Displacement Parameters (Å^2×10^3) for [Fe(L11)Cl2].2MeCN.

Atom	*x*	*y*	*z*	U(eq)
H1A	4971.75	3588.06	4645.25	42
H1B	3998.05	4439.9	4619.82	42
H2A	5254(11)	5646(18)	4001(8)	38(5)
H2B	4328(11)	5450(20)	3570(9)	45(5)
H3A	5868.01	5899.56	2700.34	39
H3B	4775.57	5791.49	2570.43	39
H4A	5248.05	4808.18	1584.09	41
H4B	6201.08	4211.79	1899.75	41
H5A	6448.4	2067.12	2248.3	40
H5B	5998.23	1081.19	1649.81	40
H6A	5065.03	-214.81	2443.99	39
H6B	6121.82	-319.31	2696.84	39
H7A	6390.18	4098.03	3846.37	38

Table S51 Hydrogen Atom Coordinates (Å×10^4) and Isotropic Displacement Parameters (Å^2×10^3) for [Fe(L11)Cl2].2MeCN.

Atom	*x*	*y*	*z*	U(eq)
H7B	6597.06	3744.23	3060.46	38
H8A	6715.89	1436.96	3429.07	37
H8B	6094.4	1749.57	4092.24	37
H9A	2724.57	1606.05	3954.34	32
H9B	2724.57	3393.95	3954.34	32
H11	2566.2	4752.99	5036.93	46
H12	2021.9	4743.13	6156.98	55
H13	1772.95	2500.01	6724.28	48
H14A	4203.73	3664.13	1380.57	32
H14B	4120.71	1882.19	1419.36	32
H15	5554.82	3285.67	634.66	51
H16A	4430.59	931.88	277.68	116
H16B	5273.89	1679.81	-174.86	116
H18A	2320.44	6757.83	3274.18	75
H18B	2112.6	8497.83	3405.47	75
H18C	1509.24	7244.34	3786.58	75

Table S52 Atomic Occupancy for [Fe(L11)Cl2].2MeCN.

Atom	*Occupancy*	Atom	*Occupancy*	Atom	*Occupancy*
N2	0.5	N3	0.5	N4	0.5
C3	0.5	H3A	0.5	H3B	0.5
C4	0.5	H4A	0.5	H4B	0.5
C5	0.5	H5A	0.5	H5B	0.5
C6	0.5	H6A	0.5	H6B	0.5
C7	0.5	H7A	0.5	H7B	0.5
C8	0.5	H8A	0.5	H8B	0.5
H9A	0.5	H9B	0.5	C14	0.5
H14A	0.5	H14B	0.5	C15	0.5
H15	0.5	C16	0.5	H16A	0.5
H16B	0.5	H18A	0.5	H18B	0.5
H18C	0.5				

Experimental

Single crystals of $C_{22}H_{35}Cl_2FeN_5$ **[[Fe(L11)Cl2].2MeCN]** were **[]**. A suitable crystal was selected and **[]** on a **Bruker APEX-II** diffractometer. The crystal was kept at 120.01 K during data collection. Using Olex2 [1], the structure was solved with the SHELXT [2] structure solution program using Intrinsic Phasing and refined with the SHELXL [3] refinement package using Least Squares minimisation.

1. Dolomanov, O.V., Bourhis, L.J., Gildea, R.J, Howard, J.A.K. & Puschmann, H. (2009), J. Appl. Cryst. 42, 339-341.
2. Sheldrick, G.M. (2015). Acta Cryst. A71, 3-8.
3. Sheldrick, G.M. (2015). Acta Cryst. C71, 3-8.

Crystal structure determination of [[Fe(L11)Cl2].2MeCN]

Crystal Data for $C_{22}H_{35}Cl_2FeN_5$ (M =496.30 g/mol): orthorhombic, space group Pnma (no. 62), a = 14.4316(7) Å, b = 8.9132(5) Å, c = 19.5405(10) Å, V = 2513.5(2) Å^3, Z = 4, T = 120.01 K, μ(MoKα) = 0.831 mm^{-1}, $Dcalc$ = 1.311 g/cm^3, 46814 reflections measured (3.508° ≤ 2Θ ≤ 56.726°), 3344 unique (R_{int} = 0.0444, R_{sigma} = 0.0174) which were used in all calculations. The final R_1 was 0.0284 (I > 2σ(I)) and wR_2 was 0.0693 (all data).

Refinement model description

Number of restraints - 235, number of constraints - unknown.

Details:

```
1. Fixed Uiso
 At 1.2 times of:
  All C(H) groups, All C(H,H) groups
 At 1.5 times of:
  All C(H,H,H) groups
2. Restrained distances
 C2-H2A = C2-H2B
 0.99 with sigma of 0.02
 N2-C2 ≈ N4_$1-C2 ≈ N4_$1-C6_$1 ≈ N2-C3
 with sigma of 0.02
3. Uiso/Uaniso restraints and constraints
Fe1 ≈ Cl1 ≈ N1 ≈ N2 ≈ N3 ≈ N4 ≈ C1 ≈ C2 ≈ C3
≈ C4 ≈ C5 ≈ C6 ≈ C7 ≈ C8 ≈ C9 ≈ C10 ≈ C11 ≈
C12 ≈ C13 ≈ C14 ≈ C15 ≈ C16: within 2A with sigma of 0.04 and
sigma for terminal atoms of 0.08 within 2A
4. Rigid body (RIGU) restrains
 Fe1, Cl1, N1, N2, N3, N4, C1, C2, C3, C4, C5, C6, C7, C8, C9, C10, C11, C12,
 C13, C14, C15, C16
 with sigma for 1-2 distances of 0.004 and sigma for 1-3 distances of 0.004
5. Others
 Fixed Sof: N2(0.5) N3(0.5) N4(0.5) C3(0.5) H3A(0.5) H3B(0.5) C4(0.5) H4A(0.5)
 H4B(0.5) C5(0.5) H5A(0.5) H5B(0.5) C6(0.5) H6A(0.5) H6B(0.5) C7(0.5) H7A(0.5)
 H7B(0.5) C8(0.5) H8A(0.5) H8B(0.5) H9A(0.5) H9B(0.5) C14(0.5) H14A(0.5)
 H14B(0.5) C15(0.5) H15(0.5) C16(0.5) H16A(0.5) H16B(0.5) H18A(0.5) H18B(0.5)
 H18C(0.5)
6.a Secondary CH2 refined with riding coordinates:
 C1(H1A,H1B), C3(H3A,H3B), C4(H4A,H4B), C5(H5A,H5B), C6(H6A,H6B), C7(H7A,H7B),
 C8(H8A,H8B), C9(H9A,H9B), C14(H14A,H14B)
6.b Aromatic/amide H refined with riding coordinates:
 C11(H11), C12(H12), C13(H13), C15(H15)
6.c X=CH2 refined with riding coordinates:
 C16(H16A,H16B)
6.d Idealised Me refined as rotating group:
 C18(H18A,H18B,H18C)
```

This report has been created with Olex2, compiled on 2020.11.12 svn.r5f609507 for OlexSys. Please let us know if there are any errors or if you would like to have additional features.
